# PA200 modulates immunoproteasome structure and activity

**DOI:** 10.1038/s41467-026-75325-w

**Published:** 2026-08-11

**Authors:** Dušan Živković, Amélie Bosc-Rosati, Angelique Sanchez Dafun, Fatme Mourtada, Przemysław Grygier, Ayse Seda Yazgili, Marijke Jansma, Michał Rawski, Anna Czarna, Stefan Bohn, Krzysztof M. Zak, André Santos Dias Mourão, Norbert Reiling, Linda Zemke, Kai Guo, Jürgen Behr, Odile Burlet-Schiltz, Grzegorz M. Popowicz, Julien Marcoux, Silke Meiners, Marie-Pierre Bousquet

**Affiliations:** 1 Université de Toulouse, CNRS, IPBS, Toulouse, France; 2 Infrastructure Nationale de Protéomique, ProFI, Toulouse, France; 3 Research Center Borstel/Leibniz Lung Center, RG Immunology and Cell Biology, Airway Research Center North (ARCN), Member of the German Center for Lung Research (DZL), Borstel, Germany; 4 Malopolska Centre of Biotechnology, Jagiellonian University, Gronostajowa 7a, Krakow, Poland; 5 Doctoral School of Exact and Natural Sciences, Jagiellonian University, Krakow, Poland; 6 Comprehensive Pneumology Center (CPC), University Hospital of the Ludwig -Maximilians-University (LMU) and Helmholtz Center Munich, Member of the German Center for Lung Research (DZL), Munich, Germany; 7 Molecular Targets and Therapeutics Center, Institute of Structural Biology, Helmholtz Munich, Neuherberg, Germany; 8 TUM School of Natural Sciences, Department of Bioscience, Bayerisches NMR Zentrum, Technical University of Munich, Garching, Germany; 9 National Synchrotron Radiation Centre SOLARIS, Jagiellonian University, Kraków, Poland; 10 Cryo-Electron Microscopy Platform and Institute of Structural Biology, Helmholtz Munich, Ingolstädter Landstraße 1, Neuherberg, Germany; 11 Research Center Borstel/Leibniz Lung Center, RG Microbial Interface Biology, German Center for Infection Research (DZIF), Partner Site Hamburg-Lübeck-Borstel-Riems, Borstel, Germany; 12 Department of Medicine V, LMU University Hospital, LMU Munich, Member of the German Center for Lung Research (DZL), Munich, Germany; 13 Institute of Experimental Medicine, Christian-Albrechts University Kiel, Kiel, Germany; 14 Present Address: University of Oxford, Department of Biology, Oxford, UK; 15 Present Address: The CRUK Scotland Institute, Garscube Estate, Glasgow, UK

**Keywords:** Cryoelectron microscopy, Proteasome, Protein-protein interaction networks

## Abstract

The proteasome activator PA200 binds to the catalytic core of both standard proteasome (s20S) and the immunoproteasome (i20S); however, whether PA200 uses the same mechanisms to activate i20S remains unknown. In this work, the cryo-EM structures of singly- and doubly-capped i20S–PA200 complexes, combined with complementary in vitro biochemical assays, show that binding of the first PA200 induces allosteric bending of the i20S and widens the opposite α-ring, promoting higher PA200 occupancy and stronger activation compared to the s20S. PA200 also selectively modulates i20S proteolytic activity by enhancing peptide production and shifting cleavage specificity toward caspase-like activity. In cells and tissues co-expressing PA200, s20S, and i20S, PA200 preferentially associates with the i20S. Moreover, PA200 and i20S catalytic subunits are differentially regulated, with PA200 playing a potential role in regulating the i20S subunits’ expression. Overall, these findings suggest that PA200 contributes to the regulation of i20S function and dynamics.

## Abstract (web summary)

Proteasomes help cells recycle proteins and shape immune responses. Here, the authors show that PA200 binds strongly to the immunoproteasome, reshaping its structure and activity to alter generation of immune-related protein fragments.

## Introduction

The proteasome is a large macromolecular machine that facilitates regulated proteolysis of protein substrates in the cell. It plays an essential role in maintaining protein homeostasis by degrading proteins that are either damaged or no longer needed by the cell. Additionally, the peptides generated by the proteolytic activity of the proteasome are also used as antigens for presentation on major histocompatibility complex (MHC) class I molecules to CD8 T cells of the adaptive immune system^1,2^. The proteasome is composed of the catalytic core particle, i.e., the 20S proteasome, and a large number of associated proteasome regulators^3^. As such, the proteasome represents a family of distinct complexes that rapidly assemble and re-assemble to fine-tune protein degradation according to cellular needs^4^.

The 20S proteasome is a barrel-shaped protease composed of two outer α and two inner β rings of seven subunits each^5^. Three of the seven β subunits form the active sites containing N-terminal threonine residues as active centers. These β1, β2, and β5 subunits of the standard 20S (s20S) proteasome possess caspase-like (C-L), trypsin-like (T-L), and chymotrypsin-like (CT-L) activities, cleaving proteins after acidic, basic, and hydrophobic amino acids, respectively^5^. In addition to the β1, β2, and β5 subunits, proteasomes can also contain an alternative set of catalytic subunits (β1i, β2i, β5i), resulting in the formation of a distinct form of the proteasome called the immunoproteasome (i20S)^6^. In general, expression of β1i, β2i, β5i is induced upon viral infection or inflammatory signaling, and the i20S plays an important role in anti-viral and anti-cancer immune responses^6^. In immune cells, the i20S is the predominant form of the proteasome^7,8^.

The 20S core particle binds to proteasome regulators, including the predominant 19S regulatory particle^9,10^, PA28αβ, PA28γ, and PA200 (also known as PSME4)^6^. While the cellular functions of 19S, PA28αβ, and PA28γ are well studied^4^, the function of PA200 is still not fully understood. Accumulating evidence indicates that the cellular function of PA200 appears to be cell type and differentiation-specific, playing a role in DNA damage repair, chromatin remodeling^11–13^, aging^14^, differentiation^15^, and mitochondrial and protein stress responses^16–19^. PA200 exerts these effects by binding the 20S catalytic core either on one side or on both sides, forming singly- or doubly-capped 20S proteasome complexes, respectively^20,21^, as well as by binding to the free end of the 26S proteasome complex^17^. In all cases, PA200 binding results in activation of proteasome activity, although the mechanism of activation is not fully solved.

Recently, we and others have demonstrated that PA200 binds the i20S. This binding appears to play an important role in abrogating antitumor immunity in lung cancer by modulating i20S activity and the antigen diversity generated through i20S proteolysis^22^. However, the mechanisms that govern these effects, as well as the molecular determinants of PA200 interactions with i20S, remain unknown.

Here, we investigate the interplay of PA200 and the immunoproteasome by combining structural analysis of i20S-PA200 complexes with biochemical and cellular analyses. We solve cryoEM structures of the i20S, singly or doubly-capped by PA200, and observe that binding of the first PA200 molecule induces structural rearrangements in the i20S - an effect not seen with the s20S. This likely explains the higher occupancy of the i20S–PA200 complex at equilibrium, as well as the stronger activation of i20S by PA200 relative to s20S. It also represents a unique feature of i20S-PA200 complexes, as such large conformational changes have not been observed in previous s20S-PA200 studies^20,21^. Consistently, in vitro unstructured protein digestion assays further reveal a stronger impact of PA200 on i20S peptide output and cleavage specificity, with a shift towards an enhanced caspase-like activity relative to s20S. By analyzing cellular and tissue data, our analyses indicate that the expression of PA200 and i20S catalytic subunits is influenced by the cellular context.

## Results

### Structural analysis of immunoproteasome-PA200 complexes

**Fig. 1:**
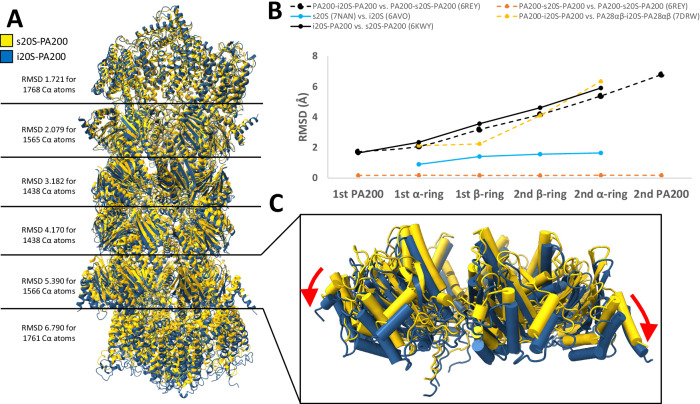
Cryo-EM analysis of the i20S-PA200 proteasome complex. The structure of doubly-capped i20S shares overall similarity to the s20S-PA200 structures reported before. **A** Overlay of the i20S-PA200 (blue) and s20S-PA200 (yellow, PDB: 6REY). The structures were aligned by the PA200 molecule at the top. The overall axis of the complex is bent. Root Mean Square Deviations (RMSD, in Å) between both structures are indicated for the two PA200 molecules and for each one of the α and β ring. **B** Comparisons of the i20S-PA200 and s20S-PA200 structures (black lines) show a progressive increase of the RMSD from the top (first binding event) to the bottom of the barrel, reflecting the long-range allosteric bending of the barrel. Comparison of PA200-i20S-PA200 vs. PA28αβ-i20S-PA28αβ (7DRW.pdb) shows similar RMDS increase (dashed yellow line), confirming that such bending is not occurring upon PA28αβ binding. As a control, we solved the structure of the PA200-s20S-PA200 complex (see Supplementary Fig. 2) and did not observe such bending when compared to the published one (6REY.pdb) (orange line). Similarly, comparison of the s20S (7NAN.pdb) vs. i20S (6AVO.pdb) structures (blue line) show only marginal deviations (see Supplementary Fig. 3). Solid and dashed lines correspond to singly- and doubly-capped complexes, respectively. Source data are provided as a Source Data file. **C** The α subunits furthest away from the aligned PA200 molecule show a substantial displacement (up to 5.4 Å).

**Fig. 2:**
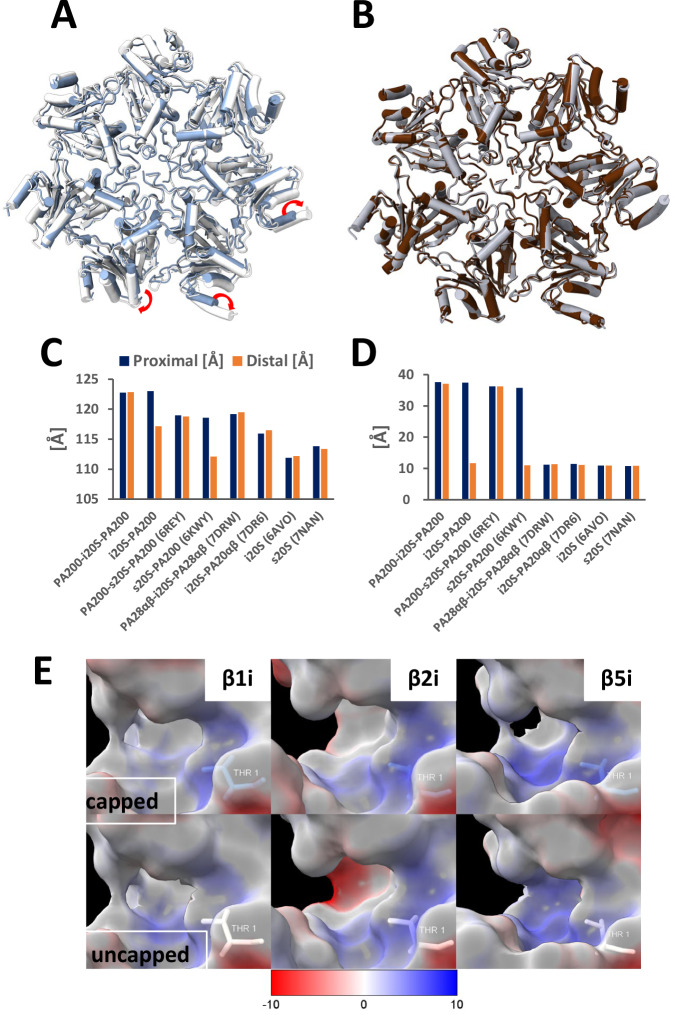
Binding of PA200 induces channel opening of i20S, allosterically alters the catalytic site and widens the opposite unbound α-ring. Superimposition of the **A** unbound side of the singly-capped i20S-PA200 (white) with uncapped i20S (blue, PDB ID: 6AVO) and **B** unbound side of the singly-capped s20S-PA200 (gray, PDB ID: 6KWY) with the uncapped s20S (brown, PDB ID: 6RGQ), **C** Measure of the proximal (blue) and distal (orange) α-ring outer diameters in different complexes show that PA200 binding to the i20S induces the largest α-ring widening. **D** Measure of the proximal (blue) and distal (orange) α-ring gate diameters shows that its apparent size is larger upon binding of PA200 compared to the apo and PA28αβ-bound form. Source data are provided as a Source Data file. **E** Comparison of active sites of the capped (top) and uncapped (bottom) i20S colored by electrostatics (positive in blue and negative in red).

Here, we obtained high-resolution structures for both singly- and doubly-capped i20S-PA200 complexes (2.85 Å and 2.89 Å resolution, respectively). Although the doubly-capped structure of the i20S-PA200 complex seems similar to s20S-PA200^20–23^ (Fig. 1A, see Supplementary Fig. 1 for details on 3D classification and Supplementary Table 1 summarizing structural and refinement statistics), the structure shows a pronounced bending of the entire complex (Fig. 1B, C) which, to our knowledge, has not been reported in previously described proteasome structures. The bend propagates from the proximal PA200 molecule, which was superimposed with the corresponding PA200 in the recently solved s20S-PA200 structure (6REY) with minimal Root Mean Square Deviation (RMSD ~ 1.7 Å; Fig. 1B, black dashed line). From that region, the structure of i20S-PA200 begins to “bend”, as indicated by the gradual increase in calculated RMSDs to its maximal value in the distal PA200 (RMSD ~ 6.8 Å; Fig. 1B, black dashed line). A morph animation from the s20S-PA200 structure (yellow) to the i20S-PA200 structure (blue, see supplementary Movie 1) shows that the bending of the i20S-PA200 results in a slight but significant decompaction of the whole complex. To rule out the possibility that the unusual bending behavior upon binding to PA200 to the i20S is an artifact, we solved the cryo-EM structure of the s20S-PA200 complex. When comparing it to the deposited 6REY.pdb structure, we found an RMSD of ~0.2 Å for both the distal and proximal PA200 molecules (Fig. 1B, orange dashed line and Supplementary Fig. 2). Moreover, when aligning the proximal α-rings of the s20S (7NAN) and i20S (6AVO), we observed an RMSD of ~1.6 Å between the two distal α-rings (Fig. 1B, blue line and Supplementary Fig. 3) showing an only minor deviation of the two apo 20S complexes regarding α-rings. Thus, in the i20S-PA200 complex the entire axis of the complex bends when compared to s20S-PA200, resulting in a major shift of the opposite unbound α-ring (Fig. 1C). Importantly, the same structural shifting/tilting behavior was evident in singly-capped i20S-PA200 structures (Fig. 1B, black solid line, Fig. 2A and Supplementary Figs. 4A–C), implying that binding of the first PA200 molecule is sufficient to induce this long-range allosteric change. Of note, we observed a similar increase in RMSD along the catalytic core when comparing our PA200-i20S-PA200 structure with the PA28αβ-i20S-PA28αβ (7DRW) (Fig. 1B, dashed yellow line), confirming that this structural bending is specific to the i20S-PA200 complexes.

To quantify these allosteric changes further, we measured the outer diameters of the proximal and distal α-rings in different complexes, including the singly and doubly-capped i20S-PA200 and s20S-PA200, the two i20S-PA28αβ and the apo i20S and s20S. Our data confirm that PA200 binding to the i20S induces the largest α-ring widening (Fig. 2A–C). Interestingly, this widening was allosterically initiated by the first binding event and further completed by the second PA200 binding event. In contrast, the same comparison of the apo s20S and s20S bound to a single PA200 regulator shows almost perfect overlay and no widening of the distal α-ring (Fig. 2B, C), indicating that this bending behavior was observed only in the i20S structures analyzed here. Of note, we identified extra density in the two positively charged grooves of PA200 (Supplementary Fig. 5) that were previously identified as inositol hexakisphosphates (IP6, phytic acid) and (5,6)-bisdiphosphoinositol tetrakisphosphate (5,6[PP]2-IP4) in the s20S-PA200 complexes^20–23^. Based on these previous results, we assigned these densities to two molecules of IP6. The exact role of this binding remains unclear.

Proteasome activators typically bind to the α subunits of the 20S core to open the inner channel, enabling substrate access. The i20S-PA200 complex shows a degree of channel opening comparable to that of the s20S-PA200 (35–37 Å; Fig. 2D), which, as expected, is significantly higher than that of the apo s20S and i20S complexes, but remarkably larger than that of the only partially open s20S-PA28αβ^23^ and i20S-PA28αβ^24^ complexes (9–11 Å) (Fig. 2D). This suggests that PA200 opens the gate of the s20S and i20S particles to a wider extent than PA28αβ. However, since the N-termini of the α-ring are missing in the i20S-PA200 and s20S-PA200 structures, this apparent wider opening of the gate is probably rather reflecting a more dynamic state of the gate, which precludes its proper density assignment. Importantly, no differences were observed in that regard when comparing the i20S-PA200 vs. s20S-PA200 structures.

Detailed analysis of the catalytic sites also revealed significant differences when comparing the structures of the i20S and i20S-PA200 complexes (Fig. 2E). The subtle structural rearrangements within active sites of all three catalytic subunits (β1i, β2i, β5i) are in line with those observed upon binding of PA200 to the s20S^20^ (Supplementary Table 2). We also observed clear changes in electrostatics (Fig. 2E) suggesting that PA200 binding induces long-distance structural changes that ultimately result in changes in catalytic activity.

Taken together, our high-resolution cryo-EM structures of singly- and doubly-capped i20S-PA200 complexes showed that binding of a single PA200 induces long-range conformational changes that are transmitted across the entire i20S and are not observed in s20S complexes or upon binding of PA28αβ to the i20S, suggesting that this behavior may be specific to PA200-bound i20S under the conditions examined. Lastly, we hypothesize that the long-distance conformational changes triggered by the first PA200 molecule binding facilitate engagement of the second PA200 molecule, leading to enhanced occupancy. In parallel, PA200 binding introduces subtle changes in structure and electrostatics within the three active sites. Together, these effects—modified binding interface and active site modulation—are likely to synergize and modify the occupancy and/or the catalytic activity.

### PA200 binds and activates the i20S more efficiently than the s20S

**Fig. 3:**
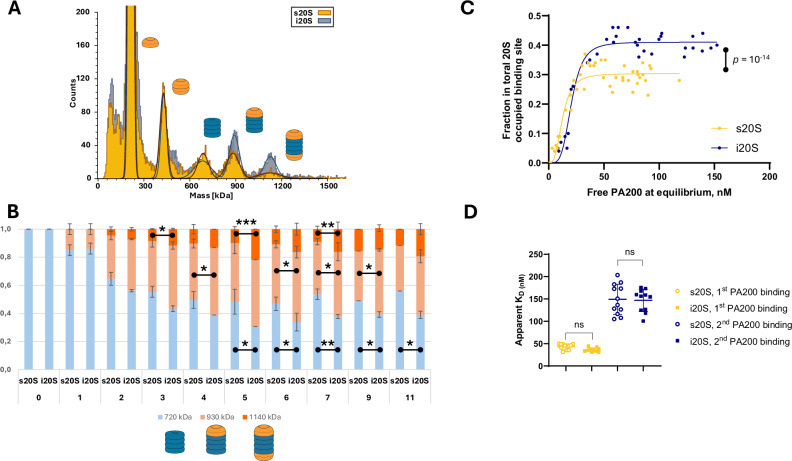
i20S particles show a higher PA200-occupancy compared to s20S complexes. **A**,**B** Mass photometry allowed us to semi-quantify the relative abundances of free 20S (720 kDa, blue), singly-capped 20S (930 kDa, light orange), and doubly-capped 20S (1140 kDa, dark orange). The PA200:20S molar ratio used in the graph shown in (**A**) was 8 but the range of molar ratios tested extends from 0 to 11 (**B**). Data are shown as mean values and standard deviations. A two-sided t-test was used, *n* = 3. **C** Titrations of PA200 to either s20S (yellow dots) and i20S (blue dots) showed a higher total occupancy of the i20S vs. s20S. The total 20S occupied binding sites were calculated by summing the fractions of singly- and doubly-capped 20S, as detailed in the Methods section. A two-sided t-test was applied for values corresponding to [PA200] above 50 nM, *p*-value = 10^-14^. **D** Apparent K_D_ values obtained from mass photometry titration points for the 1st and 2nd PA200 binding events show no difference in PA200 affinity for s20S (empty and filled yellow symbols, respectively) and i20S (empty and filled blue symbols, respectively). Data are shown as mean values, the standard deviations, and the individual values of each replicate. A two-sided t-test was applied with *: *p*-value < 0.05, **: *p*-value < 0.01, ***: *p*-value < 0.001, ****: *p*-value < 0.0001. Source data are provided as a Source Data file.

**Fig. 4:**
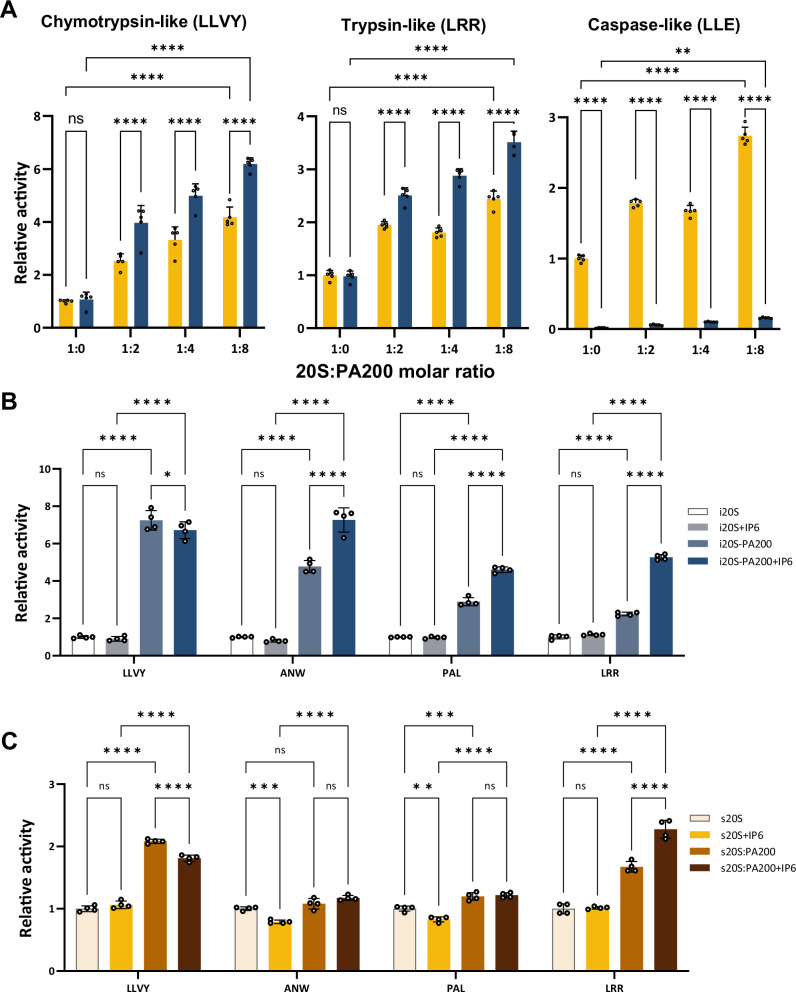
PA200 activates better the i20S and is further activated by phytic acid. **A** The three proteasome activities were assayed using isolated i20S (blue bars) and s20S (yellow bars) incubated with increasing amounts of recombinantly expressed PA200 (molar ratios 0 to 8). The LLVY-AMC, LRR-AMC, and LLE-AMC substrates were used to quantify the CT-L, T-L, and C-L activities, respectively. The activity measured with the non-activated s20S was used as the reference activity. *n* = 5. **B**,**C** Effect of phytic acid (IP6) on PA200-activated i20S (**B**, dark blue bars) and s20S (**C**, dark brown bars) catalytic activities using fluorogenic substrates specific for the distinct active sites of the i20S (LLVY, ANW, PAL, LRR substrates for β5i, β5i, β1i, β2i, respectively). PA200 was incubated first with IP6 and then with 20S at final concentrations of 21.5 nM, 70 μM and 1.5 nM for PA200, IP6, and 20S, respectively. The effect of phytic acid (IP6) on the activity measured with the free i20S (**B**, gray bars) or s20S (**C**, yellow bars) was used as the reference activity. The activity assays were performed in at least *n* = 4 replicates. Controls include free i20S (**B**, white bars) and s20S (**C**, white bars) as well as PA200-incubated i20S (light blue bars) and s20S (light brown bars) in the absence of IP6. **A–C** Data are shown as mean values, the standard deviations, and the individual values for each replicate. Two-way ANOVA with Tukey’s multiple comparison test at 95% confidence interval was used with ns: no significance, *: *p*-value < 0.05, **: *p*-value < 0.01, ***: *p*-value < 0.001, and ****: *p*-value < 0.0001. Source data are provided as a Source Data file.

To examine PA200 binding to i20S further and compare it to s20S, we used mass photometry, a method that measures molecular weights of single molecules (>40 kDa) in solution at nM concentrations^25^. For that, purified human i20S and s20S were incubated for 2 h with increasing amounts of recombinantly expressed and purified human PA200 at increasing PA200:20S molar ratios from 0 to 11. We detected free 20S, singly-capped and doubly-capped proteasome complexes (Fig. 3A). As expected, the total occupancy of the 20S increased with the PA200 to 20S ratio (Fig. 3B). We observed a significantly higher occupancy of the i20S by PA200, compared to the s20S, both in singly- and doublycapped complexes (Fig. 3B, C). Using the relative proportions of free, singly- and doubly-capped 20S measured at equilibrium by mass photometry upon titration with increasing amounts of PA200, we estimated apparent K_D_s for PA200 binding to the i20S and s20S. These were equivalent (40 ± 7 nM) for the first binding event, and 150 ± 30 nM for the second binding event (Fig. 3D). We next determined the activation of i20S vs. s20S by PA200 using fluorogenic peptides for the three active sites. Activities were determined for in vitro complexes at increasing PA200:20S molar ratios (ranging from 0 to 8). As expected from our mass photometry data, binding of PA200 to the i20S resulted in the activation of the three main types of 20S proteolytic activities, namely the CT-L, T-L, and C-L. In particular, the PA200-bound i20S was significantly more active towards CT-L and T-L sites than the PA200-capped s20S (Fig. 4). Moreover, while the baseline CT-L and T-L activities of the i20S and s20S were similar, the i20S showed very low C-L activity compared to the s20S (Fig. 4A, Supplementary Table 3), in line with published data^26,27^.

The two teams who solved the s20S-PA200 structures and identified IP6 and 5,6[PP]2-IP4 in the positively charged pockets hypothesized that they could be involved in regulating the activity of PA200, and more precisely in the degradation of acetylated histones^20,21^. In order to test this hypothesis, we assessed the effect of phytic acid on the activity of the i20S and s20S in the presence and absence of PA200 using different fluorogenic substrates specific for the distinct active sites in an in vitro substrate assay^10,28^. Despite a slight but significant decrease of the CT-L activity (LLVY substrate) for both 20S subtypes, our results show a strong increase of the three activities that are specific for the i20S, namely β1i (PAL), β2i (LRR), and β5i (ANW), in the presence of IP6 (Fig. 4B, C).

Our in vitro binding studies and activity assays further support our conclusion that binding of the first PA200 induces an allosteric widening of the opposite unbound α-ring, resulting in a higher binding occupancy of the i20S compared to the s20S. This is associated with higher occupancy and greater activation of all three active sites compared to the s20S, and more specifically of the CT-L and T-L activities that are known to favor the generation of MHC-I antigenic peptides^29,30^.

### PA200 alters peptide generation by the immunoproteasome

**Fig. 5:**
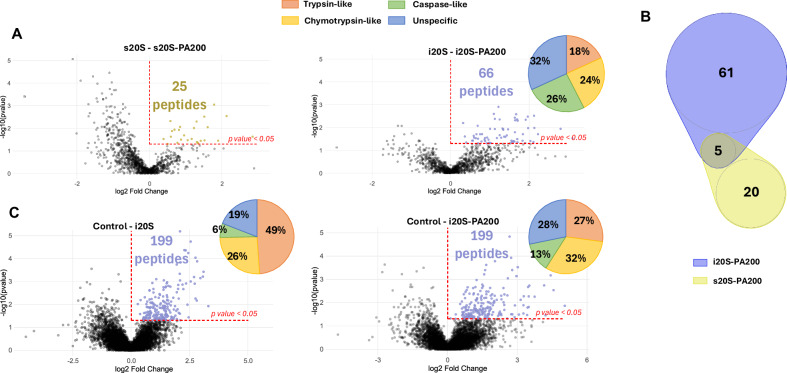
PA200 remodels peptide cleavage by the immunoproteasome. **A** Volcano plots highlighting the impact of PA200 on the C-terminal cleavage specificity and number of proteasome-derived peptides generated by the s20S (left panel) and i20S (right panel). For this analysis, we used the 1,090 proteasome-derived peptides out of the 17,615 peptides identified in total (all conditions and replicates). **B** Venn diagram showing that 61 peptides are specific for PA200-activated i20S proteasomes (blue) compared to 20 peptides generated by PA200-activated s20S (yellow). C Black dots on the volcano plots represent under-represented or non-variant peptides, i.e., unspecific peptides, and blue dots represent over-represented peptides, i.e., proteasome-derived peptides. Inserted panels: C-terminal residue analysis of proteasome-derived peptides generated by free i20S (left) and PA200-activated i20S (right), showing the shift in cleavage specificity upon PA200 activation (orange: trypsin-like; yellow: chymotrypsin-like; green: caspase-like; blue: unspecific). For all the volcano plots displayed in (**A**) and (**C**), the dashed red lines indicate the thresholds applied (FC and *p*-value; a t-test (*p*-value Benjamini-Hochberg correction) was applied, *n* = 4. Peptidomics source data were deposited with the dataset identifier PXD075459 and C-terminal residue analysis source data are provided as a Source Data file.

To assess the impact of PA200 on proteasomal peptide generation, heat-denatured HEK cell lysates were digested with purified s20S or i20S proteasomes, in the presence or absence of recombinant PA200 at a defined PA200:20S molar ratio of 8:1. Heat-denatured lysates without added proteasomes served as the respective controls to exclude non-proteasomal peptides (Supplementary Fig. 6). Heating efficiently abolished endogenous proteasome activity (99.6 ± 0.2% of the activity lost after 10 min of heating – Supplementary Fig. 7) and reactions were analyzed by LC–MS/MS after peptide isolation. Quantitative analysis using the control condition for unspecific peptide background subtraction allowed the identification of a total of 1,090 proteasome-derived peptides out of 17,615 peptides identified in total (all conditions and replicates) (Supplementary Fig. 6A). A large overlap of at least 1000 proteasome-derived peptides was identified in at least 3 replicates across the different replicates (*n* = 4 or 5, Supplementary Fig. 6B) and among these, 18 to 81% were significantly enriched in each condition (i.e. i20S, s20S, i20S-PA200, s20S-PA200) when compared to the control (i.e., without proteasome) (Supplementary Fig. 6A). Despite the relatively low number of peptides included in this analysis, the peptide length distribution is consistent with previously published results, showing that proteasome-derived peptides have a median length of 8-10 amino acids. This pattern was observed regardless of the proteasome subtype (s20S or i20S) and its activation state by PA200 (Supplementary Fig. 6C). These proteasomal peptides exhibited a significantly shorter length than unspecific under-represented peptides (i.e., peptides processed by other endogenous proteases). C-terminal residue analysis of these proteasome-derived peptides also confirmed the expected cleavage specificity of the immunoproteasome compared with s20S, characterized by reduced D/E and increased K/R and F/Y/W/L residues, corresponding to caspase-, trypsin-, and chymotrypsin-like activities, respectively (Supplementary Fig. 6A). Moreover, proteasome-derived peptides exhibited a very different C-terminal residue profile compared to unspecific peptides (Supplementary Fig. 6A). All these observations validate our experimental approach. Notably, PA200 activation had a stronger effect on i20S than on s20S, leading to the generation of a larger number of additional peptides (66 vs 25), most of which were specific to PA200-activated i20S (Fig. 5A, B) and overlapping across replicates (54 of the 66 peptides and 21 of the 25 peptides were reproducibly detected in at least three replicates for the PA200-activated i20S and s20S, respectively, Supplementary Fig. 6D). Moreover, PA200 activation of i20S was associated with a shift in cleavage specificity, indicative of decreased trypsin-like and increased caspase-like activities (Fig. 5C). In particular, 26% of peptides uniquely generated by PA200-activated i20S ended with D or E, whereas only 18% terminated with K or R (Fig. 5A, right panel).

These results are consistent with previous reports^12,22^ linking PA200 abundance to increased caspase-like and reduced trypsin-like proteasomal activity, and further support a role for PA200 in limiting the production of MHC-I–binding peptides.

### Enriched binding of i20S to PA200 in tissues

**Fig. 6:**
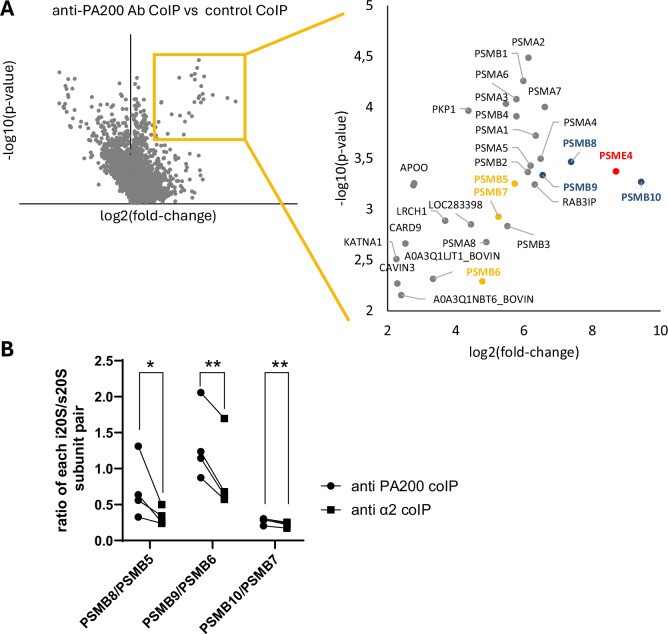
Enrichment of i20S in PA200-containing proteasome complexes in testis. **A** Immunoprecipitation of PA200 in bovine testis and subsequent MS-based interactome analysis revealed enriched binding of the i20S catalytic subunits (β1i, β2i and β5i, i.e., PSMB8-10 respectively, labeled in yellow) compared to their s20S counterparts (β1, β2 and β5, i.e. PSMB5-7 respectively). The control co-IP was performed using the OX8 IgG1 unrelated monoclonal antibody directed against rat CD8 alpha, as published earlier (PXD027436)^28^. A two-sided t-test (p-value Benjamini-Hochberg correction) was used, *n* = 4. **B** Quantification of the ratios of the corresponding catalytic i20S/s20S subunits upon PA200 pulldown (black dots) of testis tissue and compared to total 20S pulldown (black rectangles) of the same testes’ samples using an anti-α2 antibody. i20S subunits are significantly enriched compared to their s20S counterparts. A paired, two-sided t-test was used with *: *p*-value < 0.05, **: *p*-value < 0.01, *n* = 4 biological replicates. Source data were deposited with the dataset identifier PXD027436 or are provided as a Source Data file.

To examine whether PA200 engages i20S more efficiently than s20S in cells and tissues, we examined our previously generated dataset from bovine testes (PXD027436)^28^ that displays high levels of PA200 expression. Our interactome analysis of immunoprecipitated PA200 from bovine testes demonstrated enriched incorporation of the i20S subunits PSMB8-10 (Fig. 6A, in blue) compared to their s20S counterpart subunits PSMB5-7 (Fig. 6A, in yellow) into PA200-containing proteasome complexes. We confirmed enriched assembly of i20S with PA200 by comparing our PA200-pulldown data with an interactome obtained upon immunoprecipitation with an anti-α2 antibody that binds to an α subunit of the 20S proteasome and thereby pulls down all proteasome complexes within the tissue^10,31^. As shown in Fig. 6B, the ratios of the i20S/s20S catalytic subunits were significantly higher in the anti-PA200-coIP compared to the anti-α2-coIP samples. Given the fact that the anti-α2 antibody captures all 20S-containing proteasome complexes^31^, these data confirm that PA200 binds more efficiently to the i20S compared to the s20S, which is fully in line with our in vitro data on structural interactions and activation presented above.

### Regulation of i20S-PA200 interaction on the cellular level

**Fig. 7:**
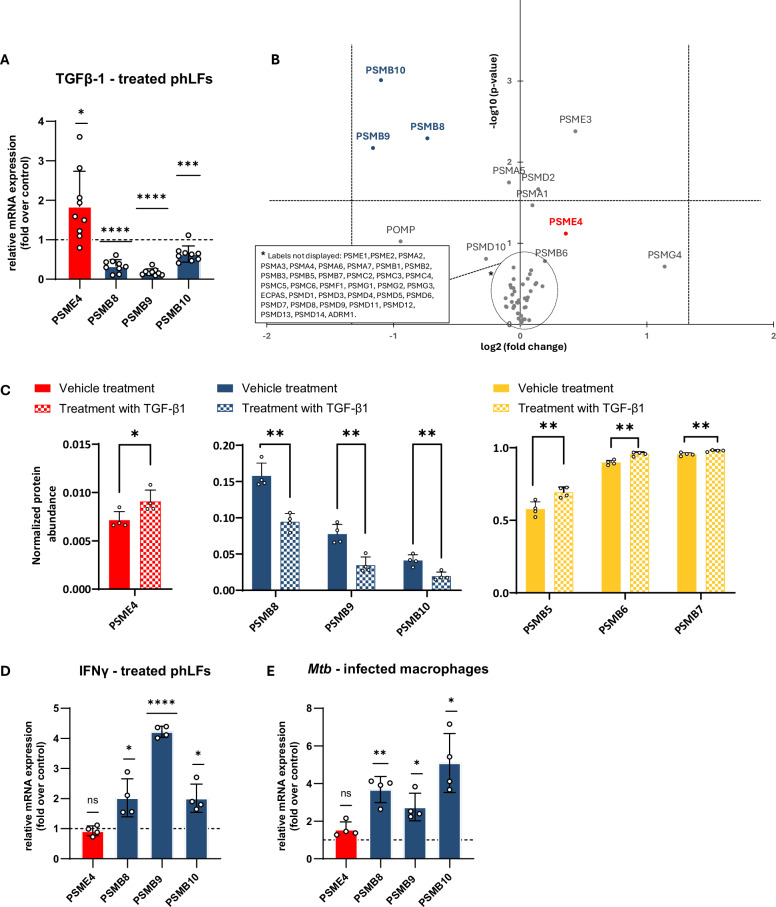
Interplay between PA200 and i20S expression in physiological contexts. RTqPCR-mediated quantification of PSME4 (PA200) and PSMB8-10 (β1i, β2i and β5i) in primary human lung fibroblasts (phLF) treated with (**A–C**) TGF-β1 or (**D**) IFNγ or in (**E**) mouse macrophages infected by *Mycobacterium tuberculosis (Mtb)*. phLFs were treated with TGF-β1 or IFNγ for 24 hours using several phLF cell lines derived from different donors and different passages (*n* = 9 independent experiments). Murine bone-marrow-derived macrophages were infected with *Mtb* for 24 h at a ratio of 3:1 (*n* = 4 independent biological experiments), and RTqPCR analysis was performed. Expression of the target gene was normalized to Ribosomal protein L19 (RPL19) (**A**,**E**) or Hypoxanthine phosphoribosyltransferase (HPRT)(**D**) and normalized to the respective control, which was set to 1, as indicated with the dashed line. Data are shown as mean values, the standard deviations, and the individual values of each replicate. One-sided sample t-test was applied with *: *p* < 0.05, **: *p* < 0.01, ***: *p* = 0.001, ****: *p* < 0.0001. **B** Volcano *p*lot comparison of proteomic analyses of the proteasome co-immunoprecipitations (CoIPs) of phLFs treated with TGF-β1 (right) vs. vehicle-treated respective controls (left). Anti-α2 proteasome subunit antibody was used for the CoIPs. A paired two-sided t-test was used. Proteomic data were filtered to display only the proteasome complex subunits and proteasome-regulating proteins. Gene names PSMB8, PSMB9, PSMB10, and PSME4 are used instead of unique protein identifiers for ease of readability. **C** Variations of abundances of CoIPed PA200, immunosubunits, and standard subunits upon TGF-β1 treatment (hatched bars) or vehicle treatment (plain filled bars), as determined by MS quantification (see Methods section, *n* = 4 independent biological replicates). The red, blue, and yellow colored bars correspond to PSME4/PA200, standard 20S catalytic subunits (PSMB5-7), and immuno 20S catalytic subunits (PSMB8-10), respectively. Data are shown as mean values, the standard deviations, and the individual values of each replicate. Two- sided t-test was applied with *: p-value < 0.05, **: *p*-value < 0.01, ***: *p*-value < 0.001, ****: *p*-value < 0.0001. Source data were de*p*osited with the dataset identifier PXD027436 or are provided as a Source Data file.

Under physiological conditions, the expression patterns of PA200 and the i20S seem to be distinct. More specifically, PA200 (PSME4) is highly expressed in adult and fetal reproductive organs, such as ovaries and testes^32^, while the i20S subunits are abundantly expressed in immune cells, such as monocytes, CD4, CD8, and natural killer (NK) cells (Supplementary Fig. 8). Therefore, we examined relationships between PA200 and i20S subunits’ expression and their regulation. To analyze the regulation of the i20S under conditions of PA200 induction, we used primary human lung fibroblasts (phLF) that were treated with TGF-β1 to upregulate PA200^15^. Vice versa, we investigated PA200 regulation under conditions of i20S activation in interferon gamma (IFNγ) stimulated phLF and upon infection of murine bone-marrow-derived macrophages with *Mycobacterium tuberculosis* (*Mtb*)^33^. On the RNA level, TGF-β1 treatment upregulated PA200 in several primary lung fibroblast (phLF) lines but uniformly downregulated the three i20S catalytic subunits PSMB8-10 (Fig. 7A). Elevated incorporation of PA200 reduced assembly of i20S subunits which was further confirmed by an interactome analysis of TGF-β1-treated phLFs compared to untreated controls (Fig. 7B, C), using the anti-α2 antibody for pulldown of all crosslinked proteasome complexes. phLFs were also stimulated with IFNγ, resulting in the upregulation of the i20S, as indicated by a significant increase in PSMB8-10 expression, but without any impact on PA200 mRNA levels as determined by RTqPCR (Fig. 7D). Similarly, *Mtb* infected mouse macrophages strongly upregulated the i20S subunits Psmb8-10 but did not alter PA200 RNA expression (Fig. 7E). These data indicate that PA200 and the i20S are differentially regulated under conditions of differentiation, cytokine treatment and infection.

### Regulation of PA200 by the immunoproteasome and vice versa

**Fig. 8:**
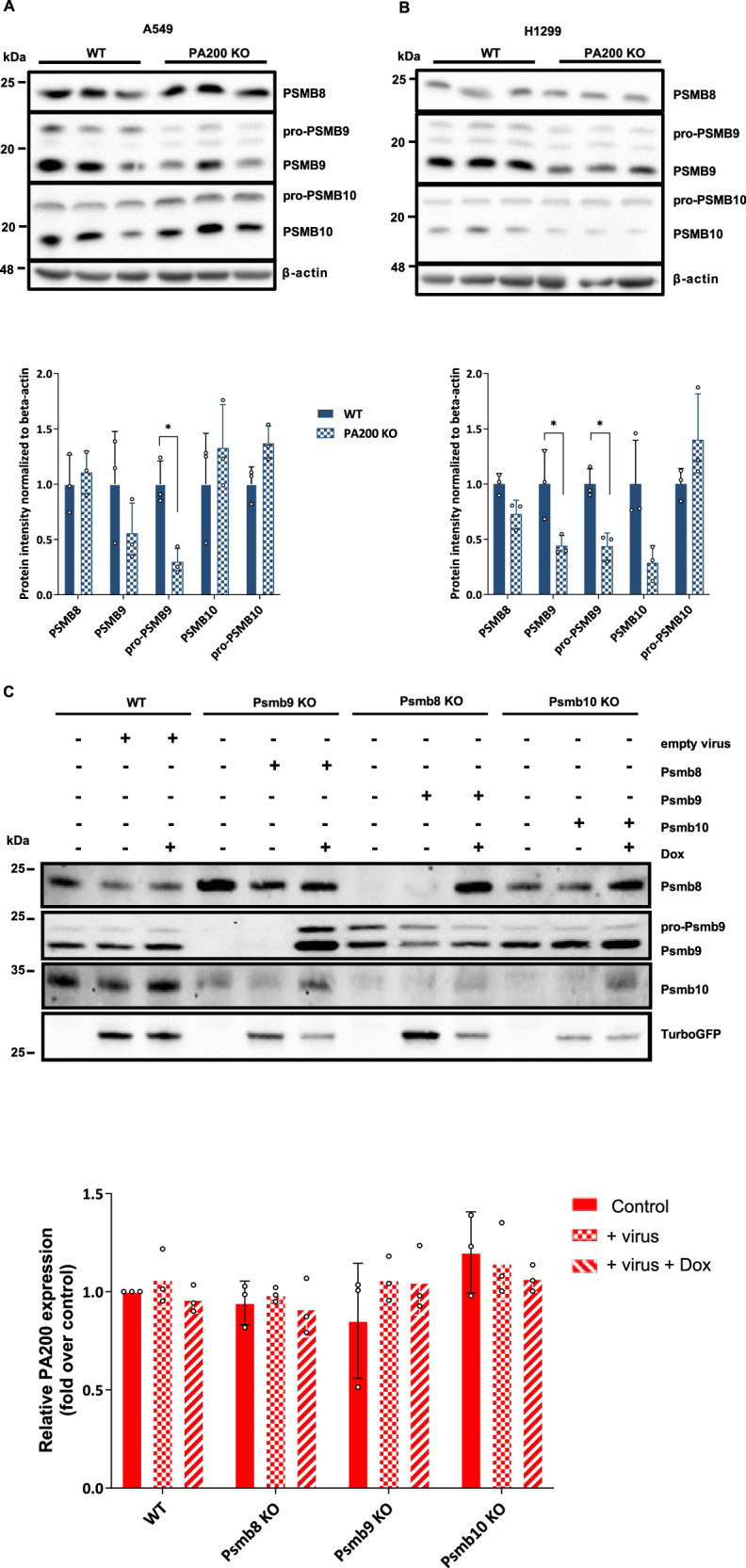
PA200 depletion reduces immunoproteasome expression but immunoproteasome deficiency does not affect PA200 expression. Expression of PA200 and i20S subunits in the human lung cancer cell lines A549 (**A**) and H1299 (**B**) upon CrispRCas9-mediated depletion of PA200. **A**,**B** Three different WT (plain filled bars) and PA200 KO (hatched bars) clones were analyzed for expression of PA200 and PSMB8-10 as well as for the proteasomal α subunits 1–7. Blots were normalized to β-actin and densitometric analysis shows quantification of expression relative to average WT levels set to 1 (*n* = 3 different clones). Unpaired- t-test was applied with *: *p*-value < 0.05. **C** Primary mouse skin fibroblasts were prepared from Psmb8, Psmb9, and Psmb10 KO mice and compared to cells isolated from WT mice. Single subunits were reconstituted into the respective KO cells using a Doxocyclin (Dox)-inducible lentivirus expressing the single mouse i20S cDNA together with green-fluorescent protein (GFP). WT cells were infected with an empty GFP-expressing lentivirus. Expression of the i20S subunits was analyzed by immunoblotting for the respective subunits. Detection of GFP served as a control for effective lentiviral transduction of cells. PA200 (Psme4) RNA expression was analyzed by RTqPCR under each condition (plain filled bars: control; hatched bars: virus infected samples; striped bars: Dox-induced samples) and expression was normalized to the house-keeping gene Ribosomal protein L19 (Rpl19) and to the WT control (unpaired t-test with Welch’s correction, *n* = 3 independent experiments, data are shown as mean values, the standard deviations, and the individual values for each replicate). Source data are provided as a Source Data file.

Given the differences in expression patterns, we wondered whether PA200 itself is involved in the regulation of the i20S and vice versa. To probe these questions, we used PA200 knockout (KO) human lung cancer cell lines A549 and H1299^26^. Genetic deletion of PA200 in A549 and H1299 reduced expression of the β1i catalytic subunit (PSMB9 gene) in both its pre-mature and mature forms but did not significantly affect expression of PSMB8 and PSMB10 (Fig. 8A, B). Pulldown of 20S complexes using the anti-α2 antibody and subsequent interactome analysis confirmed that the absence of PA200 impairs the assembly of β1i containing i20S complexes (Supplementary Fig. 9). This resulted in an altered cellular composition of 20S complexes with reduced formation of intermediate proteasomes containing the two immunocatalytic subunits β1i and β5i (β1iβ5i i20S) and an increase in complexes harboring only the immunocatalytic subunit β5i (β5i i20S) in the lung two cancer cell lines (Supplementary Fig. 9)^34,35^. We did not observe consistent regulation of the i20S subunits on the RNA level^26^. To analyze whether the i20S regulates PA200, we made use of primary mouse skin fibroblasts isolated from single i20S subunit KO mice^36^. In these cells we reconstituted the respective i20S subunits with a doxocycline-inducible lentiviral expression system (Fig. 8C). Analysis of PA200 RNA expression in i20S single KO and reconstituted cells (virus+Dox), however, did not reveal any regulation of PA200 by single i20S subunits (Fig. 8C). Our results suggest that PA200 regulates the relative amounts of i20S catalytic subunits and, thus, composition of i20S complexes, while the i20S single subunits do not affect PA200 expression.

## Discussion

In this work, we characterized the interaction of PA200 with the i20S and compared it to s20S-PA200 interactions using structural, proteomic, and cell biological approaches. We observed that the structure of the singly-capped i20S-PA200 complex differs significantly from that of the s20S-PA200. Despite an overall very similar fold and opening of the gate, our structural analysis revealed several differences. Firstly, we observed a general bend of the singly-capped i20S-PA200, not seen in the three recent s20S-PA200 structures^20–23^ or in any other proteasome activator-bound 20S complexes^23,24,37^. Second, this bend is accompanied by an increase in the outer diameter of the unbound side of the i20S compared to the s20S, displacing atoms up to 5.4 Å. Using mass photometry, we obtained evidence that the abundance of the singly- and doubly-capped complexes relative to the total proteasome particles was higher for the i20S compared to the s20S. Our data thus suggest that this allosteric bending increases the occupancy of i20S-PA200 complexes. Such increased abundances of singly- and doubly-capped i20S-PA200 complexes would thereby indirectly shift the composition of activated proteasome complexes^37^, in favor of PA200-containing particles. Third, we detected differential allosteric effects on the catalytic active sites upon binding of PA200 to i20S. These structural shifts and higher occupancy levels were associated with an increased activation of the i20S compared to the s20S upon PA200 binding, as shown by in vitro activity assays. More precisely, we observed that PA200 induced a four- to ten-fold increase in trypsin- and chymotrypsin-like activities and a significant activation of the caspase-like activity, although this activity is generally low in the i20S. This is a significantly higher level of activation compared to s20S. These divergent data for PA200-mediated activation of i20S and s20S might partly explain the conflicting reports of previous cellular studies on the activation of all three proteolytic activities by PA200 (discussed in detail in ref. ^38^). Lastly, we detected the presence of two phytic acid molecules in i20S-PA200 complexes, whereas s20S-PA200 complexes associated with both IP6 and 5,6[PP]2-IP4. We show here that IP6 further enhances the activation of the i20S by PA200, suggesting that it probably acts as a cofactor. Although the mechanism by which IP6 affects proteasome activity remains to be determined, we propose that this negatively charged molecule alters substrate entry channels in PA200, thereby affecting substrate specificity.

We confirmed that PA200 and i20S engage in cells and tissues by analyzing i20S-PA200 and s20S-PA200 complexes in testis, an organ that highly expresses PA200 and the i20S^28^. In that tissue, we discovered that PA200 binds i20S more efficiently than s20S. However, the majority of tissues do not co-express both PA200 and the i20S subunits. Along these lines, we showed that cellular conditions that upregulated PA200 (e.g., the profibrotic cytokine TGF-β1) or the i20S (e.g., IFNγ, bacterial infection) did not result in concerted transcriptional regulation, suggesting that expression of PA200 and the catalytic subunits of the i20S is differentially regulated.

Previous studies have shown that bacterial or viral infections result in downregulation of PA200^38^. These observations suggest that i20S function is fine-tuned by differential expression of proteasomal activators. For example, the PA28αβ activator, which is constitutively co-expressed with the i20S in immune cells^39–42^, has been shown to preferentially bind to the i20S^10^ and activate its proteolytic activity to generate MHC-I antigenic peptides that enhance CD8 T cell responses, *e.g*. upon viral infections^43–45^. The other member of the PA28 family of activators, *i.e*. PA28γ, is also able to bind and activate the i20S^10,46^. PA28γ, however, is exclusively expressed in the nucleus and thereby only binds to nuclear i20S. PA28γ has been shown to destroy antigenic peptides which are derived from nuclear pioneer translation products serving as an important source for tumor-derived antigenic peptides^47^. As PA28γ is upregulated in many types of tumors, it may thereby help tumors to escape from immune surveillance^47^. This type of function was also recently proposed for PA200 based on the observation that addition of recombinant PA200 to cell extracts reduced inflammatory activation of the i20S^22^. In the same cells, PA200 bound strongly to cytokine-induced i20S in pulldown experiments, which is in line with our data. The discrepancies between our data on the activation of PA200 versus the observed inhibition of inflammation-induced activation of the i20S as observed by Javitt et al. can be explained by the different experimental conditions. In our study, we performed in vitro experiments using isolated i20S and s20S and recombinantly expressed PA200, where we carefully controlled the assembly of singly and doubly capped complexes by mass-photometry. Under these controlled conditions, we observed preferential activation of the i20S activities by PA200. In contrast, Javitt et al. stimulated A549 lung cancer cells plus/minus an inflammatory TNFα/IFNγ cocktail and obtained native protein lysates, which were then supplemented with recombinant PA200. Different proteasomal activities were determined in the protein lysate using fluorogenic substrates. The addition of recombinant PA200 to cell lysates might cause a shift in the overall composition of proteasome complexes, as for example observed for the increased binding of PSME1 (Extended Data Fig. 7h^22^), thereby adding to a complex and not well-controlled effect of PA200 on proteasome activity in the cell extracts.

Our data indicate that PA200 binding to the i20S results in prominent activation of β1i, β2i and β5i active sites compared to the s20S. While an increase of the CT-L and T-L activities would favor generation of MHC-I antigenic peptides, activation of the intrinsically low C-L activity in the i20S is detrimental for the production of proper MHC-I ligands and efficient antigen presentation^22,48^. We further examined the effect of PA200 on both the quantity and C-terminal composition of proteasome-generated peptides using in vitro digestion of heat-denatured proteins. These analyses not only confirmed a greater impact of PA200 on the i20S compared with the s20S, but also revealed a pronounced shift in cleavage specificity toward enhanced caspase-like activity. Notably, PA200-activated i20S generated a higher number of additional peptides, many of which were uniquely associated with PA200 activation and exhibited a strong enrichment in acidic C-terminal residues. Together with previous reports linking PA200 abundance to increased caspase-like and reduced trypsin-like activity^22^, these findings support a model in which PA200 limits the production of optimal MHC-I–binding peptides. Thus, the ability of PA200 to significantly increase the C-L activity of the i20S could at least partly explain the reduced diversity of presented antigenic peptides and the lack of response to immunotherapy observed in lung carcinoma^22^.

In addition to the direct effect described above, whereby PA200 binding triggers long-range conformational changes resulting in i20S activation, our Western blot and proteomics analyses in two different cell lines indicate that PA200 also influences β1i protein expression. These observations suggest a potential association between PA200 and the cellular composition of the proteasome. Regulation of the i20S by PA200 may influence antigenic repertoire generation.

As such, reduced protein levels of β1i in PA200-deficient cells might contribute to an altered MHC-I antigenic diversity^22^. Indeed, the replacement of a constitutive subunit by its immunosubunit counterpart, and vice versa, can result both in generation and destruction of specific antigenic epitopes^35,49^. Transcriptional regulation of i20S catalytic subunit expression and alteration of MHCI antigen presentation has also been demonstrated for PA28γ^50^.

In conclusion, here we describe a “bent” conformation of the immunoproteasome observed upon PA200 binding. This proteasome activator shows stronger binding efficiency for immunoproteasome over the standard proteasome, and the binding results in increased proteolytic activity in all three active sites (β1i, β2i and β5i). Beyond this global activation, PA200 preferentially remodels immunoproteasome cleavage output by increasing the number of generated peptides and shifting their C-terminal composition toward enhanced caspase-like specificity. Together, our findings provide additional information on PA200 function and suggest an additional mechanism contributing to i20S regulation. Our study provides a basis for future investigations exploring the biological and pathological contexts in which PA200-mediated activation of the immunoproteasome may play a functional role.

## Methods

*Purification of s20S and i20S from HEK293-EBNA Cell Lines.* The procedure was done as described in ref. ^51^. Proteasome purification was done using MCP21, an anti-α2 proteasome subunit antibody produced in-house from a hybridoma, grafted onto cyanogen bromide-activated Sepharose beads. HEK293-EBNA cells, expressing either i20S or s20S, were lysed in a pH 7.6 lysis buffer containing 20 mM Tris-HCl, 100 mM NaCl, 10 mM EDTA, 0.25% Triton X-100, and one tablet of protease and phosphatase inhibitors per 50 mL (cOmplete™ ULTRA Tablets Mini EDTA-free and PhosSTOP, Roche, Basel, Switzerland). Lysate was sonicated with a Vibracell sonicator (10 cycles of 30 s on and 1 min off, at 50% active cycle) and the lysate clarified by centrifugation at 16,000× g for 30 min, and the supernatant was filtered through a 0.22 µm membrane. The filtered lysate was incubated overnight with MCP21 antibody-grafted beads. The following day, the beads were washed with equilibration buffer (20 mM Tris-HCl, 1 mM EDTA, 10% glycerol, 100 mM NaCl, pH 7.6) and eluted using the same buffer supplemented with 3 M NaCl. The eluate was concentrated to 0.5 mL using 100 kDa MWCO centrifugal filters and applied to size-exclusion chromatography on a Superose 6 10/300 GL column with TSDG buffer (10 mM Tris-HCl pH 7.0, 1 M KCl, 10 mM NaCl, 5.5 mM MgCl2, 0.1 mM EDTA, 1 mM DTT, 10% glycerol). Proteasome enzymatic activity was assessed, and active fractions (eluted between 10–14 mL) were pooled. Buffer exchange with equilibration buffer and concentration were performed using 100 kDa MWCO centrifugal filters. Glycerol was added to a final concentration of 20%, and aliquots were snap-frozen in liquid nitrogen and stored at −80 °C.

### Recombinant human PA200

Purification of recombinantly expressed human PA200 was done, as recently described^26^. The isolated PA200 was stored at a final concentration of 1 mg/ml to be used for cryo-EM analysis and other in vitro assays.

### Cryo EM analysis of i20S-PA200 complexes

i20S was obtained commercially from R&D Systems (Catalog #: E-370, 10 microliter, 25 microgram: Human 20S Immunoproteasome Protein, CF E-370-025: R&D Systems) and complexes were formed upon addition of 8x molar excess of PA200 upon overnight incubation. Before plunge freezing, QUANTIFOIL R2/1 copper grids (200 mesh) were cleaned using a glow-discharger (Leica EM ACE 200) at 8 mA for 60 s. 3 uL of i20S-PA200 sample (0.3 g/l) was added to the grids and plunge-frozen using a Vitrobot Mark IV (Thermo Fisher) set to 95% humidity and 4 °C with 2 s for wait time, blot force 5, and 1 s for blotting time. Micrographs were acquired at 300 kV using a Titan Krios G4i (Thermo Fisher; HZ Munich, Germany) equipped with a Selectris energy filter and a Falcon4i direct electron detector. A dataset of 23355 micrographs was collected with 0.95 A pixel size and 1.5–2.5 µm under-focus in 60 frames, accumulating 60 e/A^2 total dose.

### Image processing and volume reconstruction

Cryo-EM datasets were processed using Cryo-EM Single Particle Ab-Initio Reconstruction and Classification (CryoSPARC 4.5.3) software^52^. Imported movies were subjected to motion correction, CTF estimation, and manual exposure curation. Around 3% of micrographs were discarded because of unsatisfying CTF resolution estimation, astigmatism, or frame motion. Next, a small subset of 200 micrographs was used for preliminary particle picking with a blob picker. After picking, nearly 40,000 particles were 4x binned, extracted with a box size of 100 pixels, and 2D classified into 50 classes. Seven classes containing images of supposedly full complex (the one with two caps, i.e., PA200 complexes) comprising 4627 particles were used in ab initio reconstruction and preliminary Homo refine jobs. The obtained volume was then used to generate 50 templates for template picking on the full dataset. Picked particles were inspected, and 4768272 were again 4x binned and extracted with a box size of 100 pixels. Next, a couple of rounds of subsequent 2D classifications were made in four parallel groups containing similar numbers of particles. As most of the obtained sequential classes contained just the cup particles, only 532,792 particles were used in further processing steps. Sequential 3-class Ab-initio, Hetero-refinement, and 3D classification resulted in three subpopulations for uncapped, 1-cap, and 2-cap complexes holding 87,822, 175,565, and 156,772 particles, respectively. From the groups, preliminary binned volumes were refined, and 3% of the best particles giving the highest CTF resolution estimation were used to train the neural network Topaz picker^53^. Trained model picking resulted in 607937 picked particles, after removal of duplicates. After 2D classification, 486195 particles were selected as believed to be images of the complex. The next steps were a couple of rounds of 3D classification and Hetero refinement to separate the particles of two different complexes, i.e., 1-cap with 225565 particles and 2-cap with 187565 particles. Both densities were NU-refined and Hetero-refined on two identical volumes from NU-refine iteratively. This procedure increased the resolution and was terminated when the resolution of NU-refinement was worse than the step before. The best resolution density was again locally refined and post-processed with DeepEMhancer^54^. Finally, 2.85 Å and 2.89 Å volumes were obtained for 1-cap and 2-cap complexes, respectively.

### Model building and refinement

The PA200 model from PDB 6REY was extracted and fitted into the electron density map using the Dock in map tool in PHENIX suite^55^. The backbone geometries and orientation of the side-chains were then refined and the model was manually rebuilt using Coot^56^. We iteratively corrected steric clashes, Ramachandran and rotamer outliers manually in Coot, followed by further refinement using phenix.real_space_refine. Detailed model evaluation was done using Molprobity^57^.

### Proteasome activity assays

The proteasome substrate-based activity assays were performed in a 384-well black plate (Greiner Bio-One, UK). Purified 20S proteasome sample at 0.28 μM (either s20S or i20S) was mixed with 0.56 μM recombinant PA200 regulator in different ratios and left to interact for 30 min at room temperature (22 °C). After they were allowed to interact, the complexes were diluted with 50 mM Tris HCl pH 7.6 to a concentration of 3 nM 20S. As a control, both s20S and i20S, was diluted to a concentration of 3 nM. All diluted samples were distributed into wells in aliquots of 20 μL, to which 20 μL of 400 μM fluorogenic peptide substrate was added (prepared in 50 mM Tris-HCl pH7.6): Suc-LLVY-AMC, Boc-LRR-AMC or z-LLE-AMC, to probe for chymotrypsin-, trypsin- or caspase-like activities, respectively. The final proteasome concentration in the reaction was 1.5 nM. The kinetic assays were performed at 37 °C in a CLARIOstar Plus spectrofluorometer (BMG Labtech) over 60 min with one reading every 5 min, at 360 nm for excitation and 460 nm for emission. The gain of the fluorimeter was adjusted for each series of experiments to obtain the highest signal while avoiding saturation. The slope of the kinetic assay (increase in fluorescence intensity over time) was used to measure proteasome activity, after a 15-minute temperature equilibration time. For each series of experiments, slope calculations were performed using a minimum of five time points, and always the same kinetic times; a threshold coefficient of determination (r2) corresponding to data linearization was set at 0.98. All the slope-derived rates corresponding to Fig. 4 are shown in Supplementary Table 3. Activation effects were expressed relative to the appropriate unbound control for each proteasome type: s20S in Fig. 4A, C, and i20S in Fig. 4B.

For analysis of the effects of phytic acid on proteasome function, 0.5 μM PA200 was incubated with 1.5 mM Inositol hexakisphosphate (or IP6, Sigma-Aldrich ref P8810) at room temperature for an hour. i20S (purified from HEK293-EBNA cells) was then incubated with PA200 (with and without IP6) at 28 nM and 400 nM, respectively, at room temperature for another hour. The complexes were diluted with 50 mM Tris HCl pH 7.6 to a concentration of 2 nM i20S and assayed for activity in 384-well black plates, as described above using the following substrates: Suc-LLVY-AMC (Bachem), Ac-ANW-AMC (UBPBio), Ac-PAL-AMC (UBPBio), Boc-LRR-AMC (Enzo), z-LLE-AMC (UBPBio), Ac-nLPnLD-AMC (Biosynth), or Ac-GPLD-AMC (Biosynth) to probe for chymotrypsin-, β5i-, β1i-, trypsin- or caspase-like activities, respectively.

### Mass photometry

Proteasome (i20S purified from HEK293-EBNA, as described in Fabre et al. ^10^ or s20S purchased from Enzo) was titrated with PA200 for mass photometry as follows: Tris 50 mM pH 7.6 was added to the empty tubes on ice to compensate for different volumes caused by different theoretical protein ratios (1:0 to 1:12). 20S proteasomes were diluted to 0.2 g/L (0.28 µM) and added to each tube. Recombinantly expressed human PA200 was then added to the tubes to reach PA200:20S molar ratios ranging from 0 to 15, with a fixed 20S concentration of 120 nM. Tubes were incubated for 2 h on ice. The complexes were diluted six times immediately before measurement on the Mass photometer (Refeyn Ltd) to reach a final 20S proteasome concentration of 20 nM. Acquisition was done using a large field of view, with 60 s recording and default video settings. The acquisition was calibrated using a standard mix of IgG (150 kDa) and thyroglobulin (660 kDa) in Tris-HCl buffer. The data were analyzed in DiscoverMP 1.2 using default settings. The histograms were manually fitted with a Gaussian distribution, and the counts for each population (PA200 approximately at 200 kDa, PA200 dimer at 400 kDa, 20S at 700 kDa, 20S-PA200 at 900 kDa, and PA200-20S-PA200 at 1100 kDa) were recorded.

Tables of counts vs nominal molecular weight were then processed in Excel (Microsoft Office), and graph visualization was done in GraphPad Prism.

The experimental molar ratios were obtained based on the actual counts. The fractions of singly- and doubly-capped 20S in total binding sites were calculated by obtaining the counts for the singly-capped 20S or 2 x doubly-capped 20S divided by the total possible 20S binding sites, which was twice (i.e., two binding sites per 20S) the total 20S population (i.e., 20S alone, singly-, and doubly- capped 20S). These two fractions were then summed up to obtain the total 20S occupied binding site. The free PA200 monomer concentration at equilibrium was calculated by using the initial PA200 concentration multiplied by the fraction of free PA200 monomer. Affinity calculations were performed using the method described in Kofinova et al. 2024^58^ and, assuming a rapid re-equilibration after dilution, apparent K_D_ values were obtained using final concentrations after dilution of the different species involved.

### Co-IP of proteasome complexes and proteomics - interactomics

Co-immunoprecipitation was done using anti-PA200 antibodies (NBP1-22236, Novus Biologicals, lot: A1) or anti-α2 proteasome subunit antibodies for hPLF samples and for bovine testis samples (Gentaur)^28^. The proteasome complex enrichment procedure for cultured cells is described in detail in ref. ^28^.

Briefly, the material is homogenized using a Potter device (testis) or a cell scraper (hPLFs) in native buffer, then centrifuged to remove cell debris. Magnetic Protein G beads were incubated with anti-PA200 or anti-α2 proteasome subunit antibodies, and the antibodies were then cross-linked to the beads via 20 mM dimethyl pimelimidate treatment in 0.2 M triethanolamine (pH 8.2). Clarified lysate was then incubated with the magnetic beads coated in antibodies overnight at 4 °C. The beads were washed 4 times in lysis buffer, and enriched proteasome complexes were eluted from the beads using 5% SDS. The samples were prepared for proteomics using the S-Trap protocol, as described by the supplier (Protifi).

LC-MS method with a bioinformatic approach for interactomics is also described in ref. ^28^. Briefly, a standard nano-flow reverse phase LC-MS proteomic method was used, in DDA mode, on an Ultimate 3000 LC system and an Orbitrap Fusion MS instrument. Bottom-up LC-MSMS and Bottom-Up bioinformatic searches were done as described in Appendix 1 of Zivkovic et al. 2022^28^, with the exception that the FASTA-derived in silico library was human, from the Uniprot Proteome database. The proteomic search was done using the Proline suite, an easy-to-use, open-source bioinformatic tool developed in-house^59^.

For the analysis of the PA200 KO cell cultures, the same lysis conditions, co-immunoprecipitation, and protein digestion procedure were followed as described in detail in ref. ^28^, however, a different instrumental setup was used for the proteomic analysis.

Digested peptide extracts were desalted on an Evotip C18 EV2001 tip (Evosep) and were analyzed by online nanoLC using an UltiMate 3000 RS nano-LC system (Thermo Scientific) coupled with a TimsTOF SCP mass spectrometer (Bruker). Peptides were separated on a C18 Aurora column (25 cm × 75 µm ID, IonOpticks) using a gradient ramping from 2 to 20% of B in 30 min, then to 37% of B in 3 min and to 85% of B in 2 min (solvent A: 0.1% Formic Acid (FA) in H2O; solvent B: 0.1% FA in Acetonitrile (ACN)), with a flow rate of 150 nL/min. MS acquisition was performed in DIA-PASEF mode on the precursor mass range [400–1000] m/z and ion mobility 1/K0 [0.64-1.37]. The acquisition scheme was composed of eight consecutive TIMS ramps using an accumulation time of 100 ms, with 3 MS/MS acquisition windows of 25 Th each. The resulting cycle time was 0.96 s. The collision energy was ramped linearly as a function of the ion mobility from 59 eV at 1/K0 = 1.6 Vs.cm−2 to 20 eV at 1/K0 = 0.6Vs.cm−2. The raw data were searched and quantified with DIA-NN 1.9, using a predicted library from the UniProt human reference proteome. The result files were then imported into Proline^59^ for validation and label-free quantitation with a False Discovery Rate (FDR) of ≤ 1.0 %, peptide length range set at 7–30, and precursor charge states of 2+ and 3 + . For statistics, a t-test p-value threshold of 0.01 was applied at both peptide and protein levels. Only specific peptides were used for quantification, and the median ratio fitting was chosen as the abundance summarizer method. Normalization, missing values inference (if strictly less than three values, 5% centile was applied), t-test and z-test (p-value Benjamini-Hochberg correction) were also applied.

### Ex-cellulo degradation of proteins by proteasomes and peptidomics

HEK293-EBNA cells were lysed with 1 mL lysis buffer at pH 7.6 containing 20 mM Tris HCl, 0.02% DDM, 10 mM KCl, 10 mM EDTA, 5 mM MgCl_2_, and 1 tablet of protease in 50 mL (cOmplete™ ULTRA Tablets Mini EDTA-free, Roche), sonicated (Bioruptor Plus, Diagenode; 20 cycles, 30 sec on and 30 sec off) and then centrifuged at 14,000 g for 30 min at 4 °C. The supernatant was then heated in a thermomixer for 10 min at 95 °C to denature proteins. A further centrifugation at 14,000 g and 15 min at room temperature was performed to collect the heat-soluble proteins. Proteasome substrate-based activity assays with the LLVY fluorogenic peptide were used to verify that the endogenous proteasome activity was completely abolished by the heating step. The buffer was exchanged to digestion buffer (50 mM Tris, pH 7.5, 150 mM NaCl) on a 3 kDa MWCO Vivaspin 500 centrifugal concentrator. Purified s20S or i20S and PA200 were incubated for 1 h at 4 °C at a controlled PA200:20S molar ratio of 8:1 and then 8 µg of this mixture were added to 100 ug of protein lysate and incubated 3 h at 37 °C until the reaction was stopped by adding 5 µM Bortezomib. Proteasomal peptides were subsequently separated from proteins by ultrafiltration on a 3 kDa MWCO Vivaspin.

2 µg of peptides were desalted on Evotip C18 tips and 50 ng were analyzed by nanoLC MS/MS using the TimsTOF SCP mass spectrometer as described above.

The raw data were searched and quantified with Spectronaut (Biognosis) V 20.4 using targeted peak picking strategy (DirectDIA workflow) with a predicted library from the UniProt human reference proteome, including Frankenfield contaminants library. The following processing parameters were employed: the digest type was set as unspecific; a maximum of 3 missed cleavages was permitted; peptide length range was set at 5-52 and precursor charge states at 1-7 + ; N-terminal acetylation and methionine oxidation were searched as variable modifications; FDR was equal to 1% at peptide and protein level. Normalization across samples was made using the sum of the values from each sample and a t-test (p-value Benjamini-Hochberg correction) was also applied.

### Cell isolation, culture, and treatment

A549 and H1299 non-small cell lung cancer cell lines were grown at 37 °C and 5% CO_2_ in a humidified incubator in DMEM/F12 or RPMI-1640 medium, respectively, with supplementation of 10% FBS and 100 U/mL penicillin/streptomycin. CRISPRCas9-mediated genomic depletion of PSME4 (gene name for PA200) has been described previously by us in detail^26^.

Primary human lung fibroblasts (phLF) obtained from organ donors were used as described previously^15^. Cells were provided by the UGMLC Giessen Biobank (member of the DZL Platform Biobanking) complying with the Declaration of Helsinki. The study protocol was approved by the Ethics Committee of the Justus-Liebig-University Giessen (No. 111/08 and 58/15), and informed consent was obtained in written form from each subject. Cells were cultured in MCDB medium supplemented with 10% (v/v) FBS (Biochrome), 100 U/mL penicillin/streptomycin (Gibco, Thermo Fisher Scientific), 2 mM L-glutamine (Thermo Fisher Scientific), 5 µg/mL insulin (Thermo Fisher Scientific), 2 ng/mL basic-FGF (Thermo Fisher Scientific), and 0.5 ng/mL human EGF (Sigma-Aldrich). Cells were allowed to grow to 90% confluency before splitting. For subculturing, cells were rinsed with PBS, treated with 0.25% Trypsin-EDTA (Sigma) for 4–5 min at 37 °C, re-suspended in fresh culture medium, and transferred to new dishes. All experiments were conducted using phLF between passages 3 and 5. Prior to treatment of phLF, 0.3 ×10^6^ cells were seeded into 10 cm plates for 24 h. The cells were then synchronized by culturing them in modified MCDB medium (see above) containing 1% FBS for 24 h. Following synchronization, the medium was refreshed, 5 ng/ml TGF-β1 or 75 U/ml IFNγ were added into the culture medium for 48 h. Cell harvest was achieved by washing the cells with PBS and then detaching the cells by adding 0.25% Trypsin-EDTA and incubating the plates for 5 min at 37 °C. The process was stopped by adding culture media with 10% FBS, and the collected cells were then centrifuged in a 15 ml falcon tube at 204 g for 5 minutes. Those cell pellets were washed once more with PBS in 1.5 ml Eppendorf tubes before their storage at -80° until further use.

The method used for *primary mouse skin fibroblast* isolation was reported previously^60^ with minor modifications regarding the culture medium where DMEM was supplied with 10% FBS (Capricorn) and the antibiotic 1×Antimycotic (Gibco, 15240062) was used. WT and Psmb8, Psmb9, and Psmb10 KO mice were housed under specific pathogen-free conditions in the animal facility of the RCB in accordance with relevant national guidelines and regulations. Euthanasia of the mice and the subsequent organ harvesting for the generation of BMDMs were authorized by the Ministry for Energy Transition, Climate Protection, Environment and Nature (Kiel, Germany; V313-72241.123-3).

Bone-marrow-derived mouse macrophages (BMDM) were generated from C3HeB/FeJ mice as previously described^61^. Animal breeding for isolation of primary cells was done according to national and international standards. BMDMs were then cultured at 5 × 10^5^ cells/well and incubated overnight in 24-well plates (Nunc-surface). Subsequently, cells were infected with *Mycobacterium tuberculosis* H37Rv at a multiplicity of infection (MOI) of 3:1 for 24 h. Total RNA of cells lysed in TRIzol (peqGOLD TriFast, VWR International) was extracted by use of DirectZol RNA MiniPrep (Zymo Research) according to the manufacturer’s instructions. For reverse transcription of RNA, the Maxima First Strand cDNA Synthesis Kit for RT-qPCR (Thermo Fisher Scientific) was used.

### RNA isolation of cells

Total RNA from cells was extracted using phenol-chloroform extraction with TRIzol Reagent invitrogen). Cells were resuspended in TRIzol Reagent, followed by vigorous mixing with chloroform in Phasemaker tubes. The samples were then incubated for 5 min and centrifuged at 15,000 g for 5 min at 4 °C to achieve phase separation. The upper aqueous phase was carefully transferred to a new tube, and 500 µL of isopropanol was added. The samples were incubated for 10 min on ice. RNA was pelleted by centrifugation at 15,000 g for 10 min at 4 °C. The supernatant was discarded, and the RNA pellets were washed with 70% (v/v) ethanol. After drying on ice, the RNA pellet was dissolved in 30 µL of nuclease-free water and incubated in a heat block at 55 °C for 10 min, and the RNA concentration was measured at 260 nm using the NanoDrop 1000 (ThermoFisher).

### Reverse transcription (RT) of mRNA and RT-qPCR

For reverse transcription, 0.5 to 1 µg RNA was diluted to 14 µL with nuclease-free water and combined with a 6 µL mastermix of Maxima First Strand cDNA Synthesis Kit for RT-qPCR according to the manufacturer’s instructions (ThermoFisher). The reaction was terminated by heating at 85 °C for 5 min. The samples were then diluted 1:5 with nuclease-free water.

Quantitative real-time PCR was carried out using a SYBR Green LC480 system (Roche). Each well in the 96-well plate contained a mixture of 2.5 µL cDNA and 5 µL LC480 SYBR Green I Master mix (Roche), along with 2.5 µL of forward and reverse primers (Table 1), resulting in a final concentration of 0.5 µM. All samples were measured in duplicate, and plates were centrifuged at 91 g for 2 min before beginning the measurement, following the standard protocol of the Light Cycler 480II (Roche). Gene expression levels were normalized to the housekeeping gene Hypoxanthine-guanine Phosphoribosyltransferase (HPRT) or Ribosomal Protein L19 (RPL19).

**Table 1:** Human Primer sequence table

Target	Forward primer sequence	Reverse primer sequence
PSME4 (PA200)	CCA ACA GGA AAA GAA TGC CGA	CCA GGG CAG GTT TCT TTG CT
PSMB8	GCTATTCTGGAGGCGTTGTC	AGGCCTCTTCTTCTCCTTGG
PSMB9	ATG CTG ACT CGA CAG CCT TT	GCA ATA GCG TCT GTG GTG AA
PSMB10	AGC CCG TGA AGA GGT CTG G	CAT AGC CTG CAC AGT TTC CTC C
RPL19	TGT ACC TGA AGG TGA AGG GG	GCG TGC TTC CTT GGT CTT AG
HPRT	TGA AGG AGA TGG GAG GCC A	AAT CCA GCA GGT CAG CAA AGA A

### Protein extraction and quantification, SDS-PAGE, and Western blot

In order to evaluate intact and active proteasome complexes, cell pellets were lysed under non-denaturing conditions. Cell pellets were resuspended in OK40 buffer (50 mM Tris-HCl, 5 mM MgCl_2_ 10% Glycerol, 0.05% NP-40, 2 mM ATP) containing a 1x cOmplete™ protease inhibitor cocktail (Roche) and 1x phosphoSTOP (Roche) and lysed through vigorous pipetting followed by a 20 min incubation on ice with additional vigorous pipetting and vortexing. The lysates were then centrifuged at 15,000 g for 20 min at 4 °C. The supernatant was transferred to new tubes for immediate protein concentration determination using a BCA assay. A bovine serum albumin (BSA) calibration curve, with concentrations ranging from 0 to 2 µg/µL in PBS, was used as a standard for protein quantification. To perform the assay, 20 µL of BSA standard, 2 µL of protein lysate or pure lysis buffer diluted 18 µL in PBS were combined with 200 µL of BCA reagent, following the manufacturer’s instructions (Thermo Fisher Scientific). After a 30-minute incubation at 37 °C, absorbance was measured at 562 nm using a plate reader for subsequent protein concentration calculation.

15 µg of protein were used per sample for western blotting, and 25 µg for ABP assay. Protein extracts were diluted to equal volumes with Milli-Q® water, then mixed with 6x Laemmli sample buffer. The protein mixture was heated to 95 °C for 10 min to denature the proteins. For protein electrophoresis, samples were loaded onto 12% SDS polyacrylamide gels. Protein samples, along with a Protein Marker (#26616, ThermoFisher) were loaded onto SDS polyacrylamide gels. Electrophoresis was carried out using Bio-Rad gel running chambers. Following electrophoresis, proteins were transferred to a polyvinylidene fluoride (PVDF) membrane (Bio-Rad) using the tank immunoblotting method. The membrane was first activated in pure methanol, and the transfer was carried out at a constant current of 250 mA for 90 min or an overnight transfer of 40 mA for 960 min. To block nonspecific binding, the PVDF membrane was incubated in Roti®-Block solution (Carl Roth) for 1 h. The membrane was then incubated with the primary antibody, diluted in Roti-Block solution, either overnight at 4 °C or for 1 h at room temperature (RT). Afterwards, the membrane was washed three times with PBST (PBS, 1% Tween-20) for 5 min each and incubated with a horseradish peroxidase-conjugated secondary antibody, diluted 1:20,000 in PBST, for 60 min at RT on a shaker. The membrane was then washed three more times with PBST for approximately 20 min in total, and proteins were detected using a chemiluminescent substrate according to the manufacturer’s instructions. Protein signals were detected with the iBright CL750 (ThermoFisher). The following antibodies were used: anti-LMP2 (ab242061, Abcam, lot: 1028548-15, 1:1000 dilution), anti-LMP7 (ab3329, Abcam, lot: 1043692-3, 1:1000 dilution), anti-MECL1 (ab183506, Abcam, lot: 1006225-9, 1:1000 dilution), anti-TurboGFP (TA150041, OriGene, 1:1500 dilution), anti-GAPDH (14C10, Cell Signaling, lot: W004, 1:8000 dilution), anti-PA200 (NBP1-22236, Novus Biologicals, lot: A1, 1:5000 dilution).

### Lentivirus design and production

Lentiviruses were constructed using the pCW57-MCS1-P2A-MCS2 (GFP) transfer vector (#80924, Addgene). The cDNAs for the three different immunoproteasome subunits LMP7, LMP2, and MECL-1 were amplified from mouse embryonic fibroblasts using primers containing restriction sites for NheI-mediated cloning into the vector. The vector pCW57-MCS1-P2A-MCS2 (GFP) was linearized with the restriction enzyme NheI (NEB, #R3131), whose restriction site is located upstream of the P2A region. The following oligonucleotides (obtained from Eurofins, Germany) were used for cloning:

5’-3’, homologous arm in italic):

pCW57-LMP2-GFP_fwd: *CAGATCGCCTGGAGAATTGG*ATGCTGCGGGCAGGAGCA

pCW57-LMP2-GFP_rev: *AACCGGTGTCGACGAATTCG*TCACTCATCGTAGAATTTTGGCAGCTCATC

pCW57-LMP7-GFP_fwd: *CAGATCGCCTGGAGAATTGG*ATGGCGTTACTGGATCTGTGC

pCW57-LMP7-GFP_rev: *AACCGGTGTCGACGAATTCG*TCACAGAGCGGCCTCTCC

pCW57-MECL1-GFP_fwd: *CAGATCGCCTGGAGAATTGG*ATGCTGAAGCAGGCAGTG

pCW57-MECL1-GFP_rev: *AACCGGTGTCGACGAATTCG*TCATTCCACCTCCATGGC

The cDNAs of the individual immunoproteasome subunits were then integrated into the linearized vector after digestion with NheI using the NEBuilder® HiFi DNA Assembly Master Mix (NEB, #E2621). The construct allows inducible expression of the gene of interest by addition of Doxycycline (Dox). The activity of the tetracycline response element (TRE) promoter is inhibited by a reverse Tetracycline repressor (rTetR), which is expressed by a downstream hPGK promoter, when Dox is absent. In contrast, addition of Dox allows the release of the rTetR from the TRE promoter, thereby allowing expression of the gene of interest. The turboGFP gene is driven independently of Dox by the hPGK promoter and allows sorting of lentivirus - infected cells.

5×10^6^ 293 HEK-T cells were seeded into 10-cm cell culture plates 24 h before the plasmid transfection. Before plasmid transfection, fresh and pre-warmed DMEM supplied with 10% FBS, 25 mM HEPES and 1% L-glutamine was added to the culture plate. 8 μg of transfer plasmid (pCW57-MCS1-P2A-MCS2 (GFP) was used as control; pCW57-LMP2-P2A-GFP, pCW57-LMP7-P2A-GFP or pCW57-MECL1-P2A-GFP together with 6 μg pSPAX2 (Addgene) and 4 μg pMD2.G (Addgene) were transfected using PEI (Merck, 919012). Cell culture medium was replaced after 8 h of transfection, and lentivirus-containing medium was collected 48 h after medium replacement. Collected lentivirus-containing medium was filtered with 0.45 μm sterile filters prior to use and stored at −80 °C.

### Lentivirus infection and Dox induction

Animal breeding for isolation of KO cells was done according to national and international standards. For Lentivirus infection, 3×10^5^ WT or immunoproteasome-deficient skin fibroblasts were seeded into 6-cm plates 24 h before infection. On the day of infection, either empty lentivirus or lentivirus containing the cDNAs of β1i (Psmb9), β5i (Psmb8), or β2i (Psmb10) was added to the plates with a multiplicity of infection (MOI) of 3 for 48 h. Simultaneously, polybrene (Merck, TR-1003) with a final concentration of 8 μg/ml was added to the medium to enhance infection efficiency. After 48 h, fibroblasts were washed with PBS to remove residual polybrene and lentivirus. Afterwards, 1 μg/ml Doxycycline (Dox) (Merck, D5207) was applied to infected cells for 96 h to induce expression of the target gene and turbo GFP. Dox was added to the medium every 48 h.

### Reporting summary

Further information on research design is available in the Nature Portfolio Reporting Summary linked to this article.

## Supplementary information

Supplementary Information

Description of Additional Supplementary Files

Supplementary Movie 1

Reporting Summary

Transparent Peer Review file

## Source data

Source data

## Data Availability

The mass spectrometry proteomics data concerning A549/H1299 PA200 KO cells and TGF-β treated phLF have been deposited to the ProteomeXchange Consortium via the PRIDE^62^ partner repository with the dataset identifier PXD061729. The anti-PA200 and anti-α2 CoIP proteomics data in testes were previously deposited with the dataset identifier PXD027436^28^. [https://proteomecentral.proteomexchange.org/cgi/GetDataset?ID=PXD027436]. The peptidomics data were deposited with the dataset identifier PXD075459 [http://proteomecentral.proteomexchange.org/cgi/GetDataset?ID=PXD075459]. The cryo-EM dataset is accessible through the figshare repository^63^. The structure coordinates have been deposited in the Protein Data Bank (PDB) under the IDs 31HA (20S immunoproteasome in complex with two PA200 activators) and 31HB (20S immunoproteasome in complex with PA200 activator). The cryo-EM density maps have been deposited in the Electron Microscopy Data Bank (EMDB) under the accession codes EMD-58410 and EMD-58411 for the corresponding complexes, respectively. Source data are provided with this paper.
