# Supplementary material for: PA200 modulates immunoproteasome structure and activity
Source: Nat Commun. 2026 Aug 11;17:8175. doi: 10.1038/s41467-026-75325-w (PMC13463089; doi:10.1038/s41467-026-75325-w)
Supplement: Supplementary file 1 [file 41467_2026_75325_MOESM1_ESM.pdf]

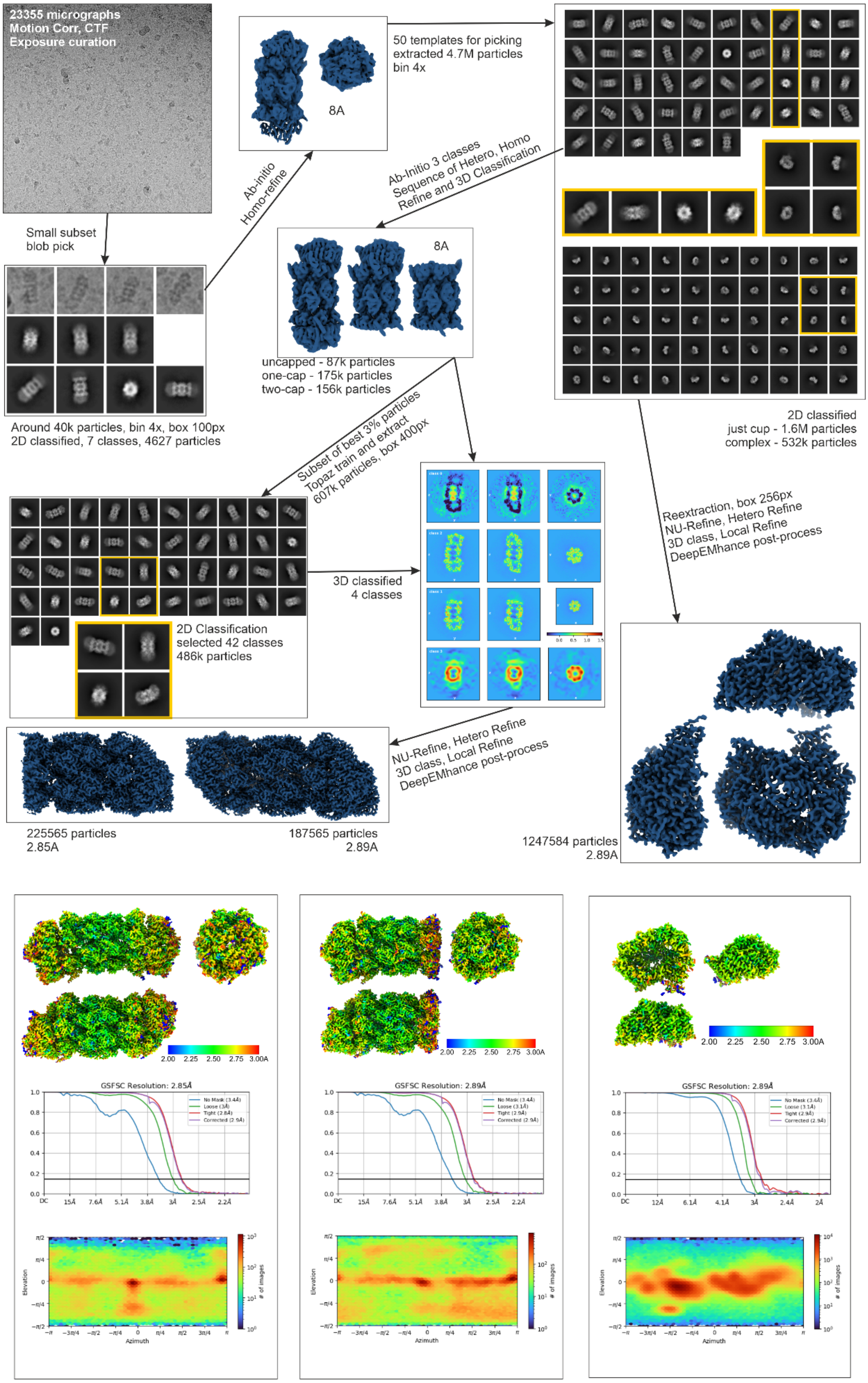

Supplementary Figure 1. Cryo-EM data processing and 3D classification pipeline with local resolution colouring maps, global FSC cutoffs and viewing direction distribution of particles.

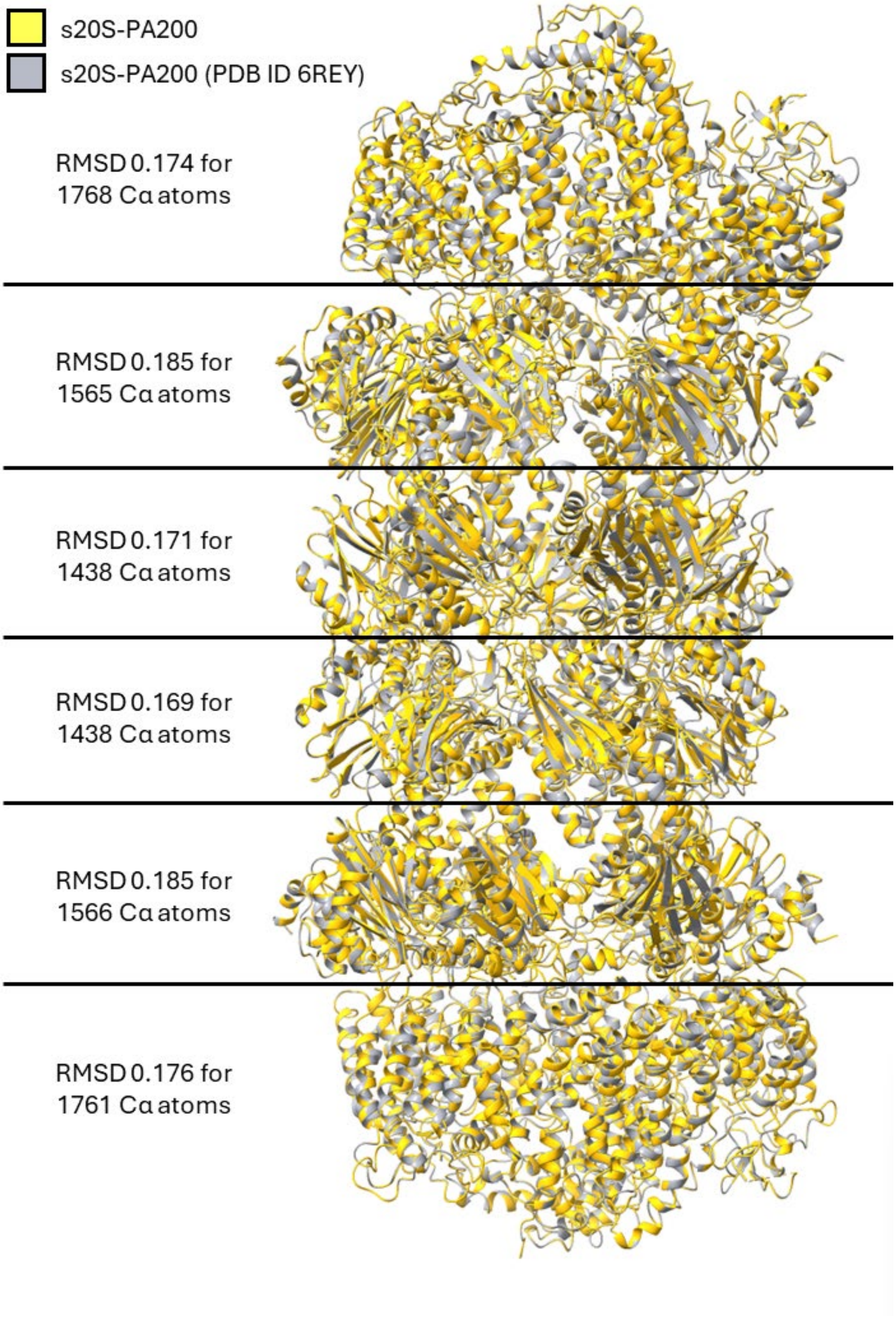

Supplementary Figure 2: Cryo-EM analysis of the 20S-PA200 proteasome complex. Overlay of the s20S-PA200 (yellow) solved in this work, with a previous structure of s20S-PA200 (grey, PDB: 6REY). The structures were aligned by the PA200 molecule at the top. Root Mean Square Deviations (RMSD, in Å) between both structures are indicated for the two PA200 molecules and for each one of the α and β ring and show almost no difference.

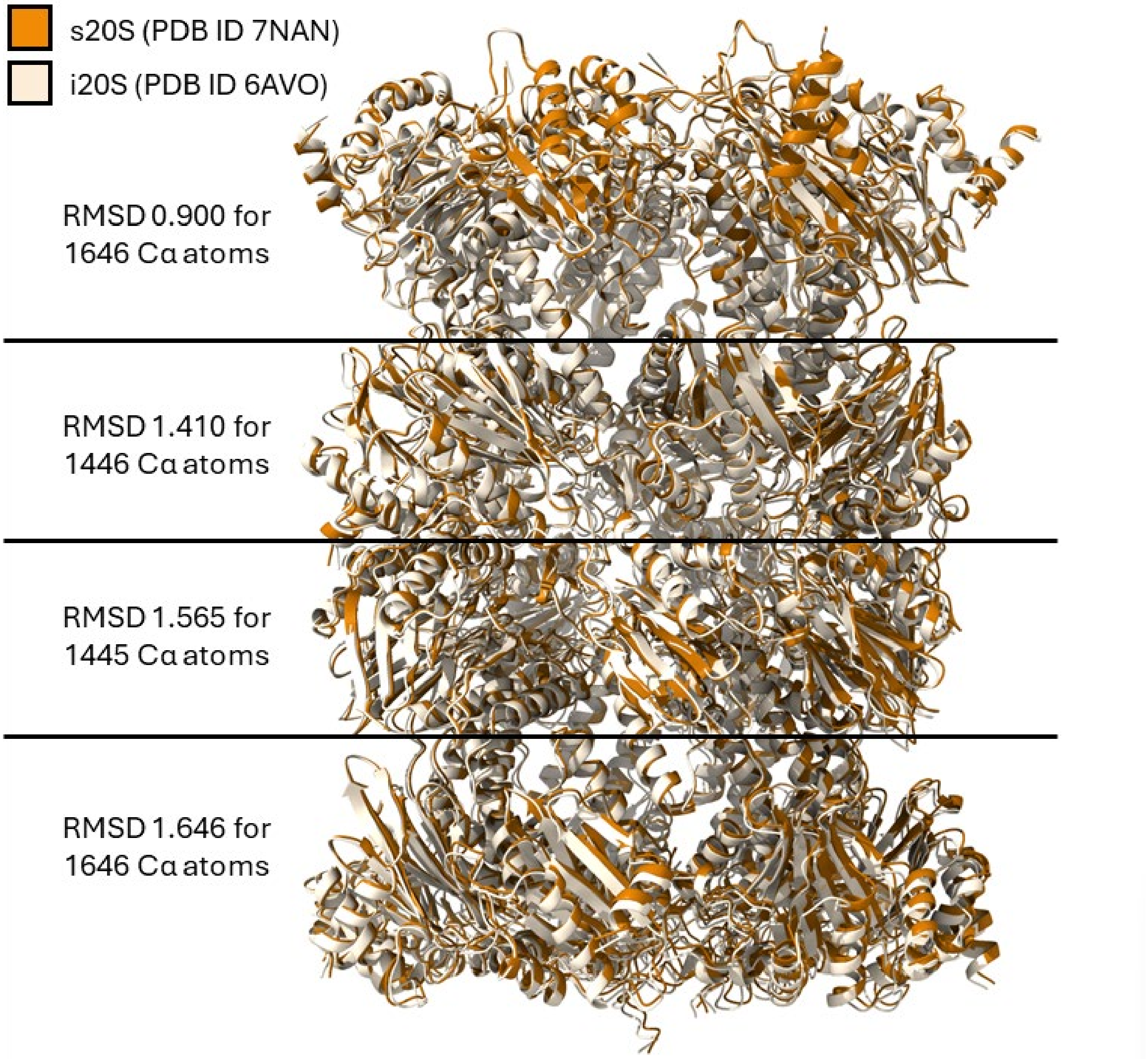

Supplementary Figure 3: Comparison of the s20S and i20S complexes. Overlay of the s20S (dark orange, PDB 7NAN) and i20S (light orange, PDB 6AVO) complexes. The structures were aligned by the α-ring at the top. Root Mean Square Deviations (RMSD, in Å) between both structures are indicated for each one of the α and β ring and show almost no difference.

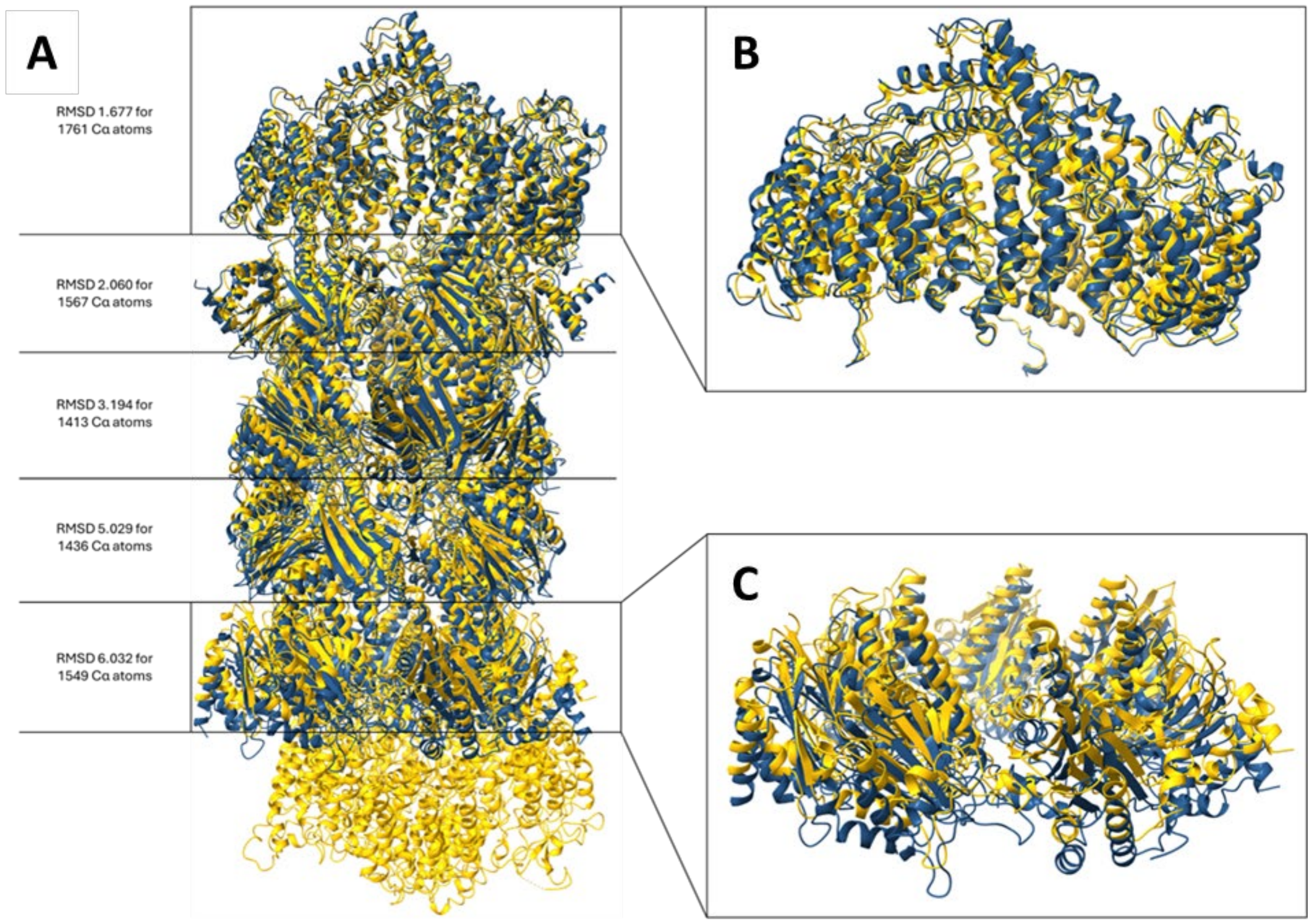

Supplementary Figure 4: (**A**) Structure of the singly-capped i20S-PA200 complex (in blue) overlaid onto the doubly-capped s20S-PA200 complex (in yellow). The misalignment of the domains far away from the aligned PA200 molecule (**B**) is evident in (**C**).

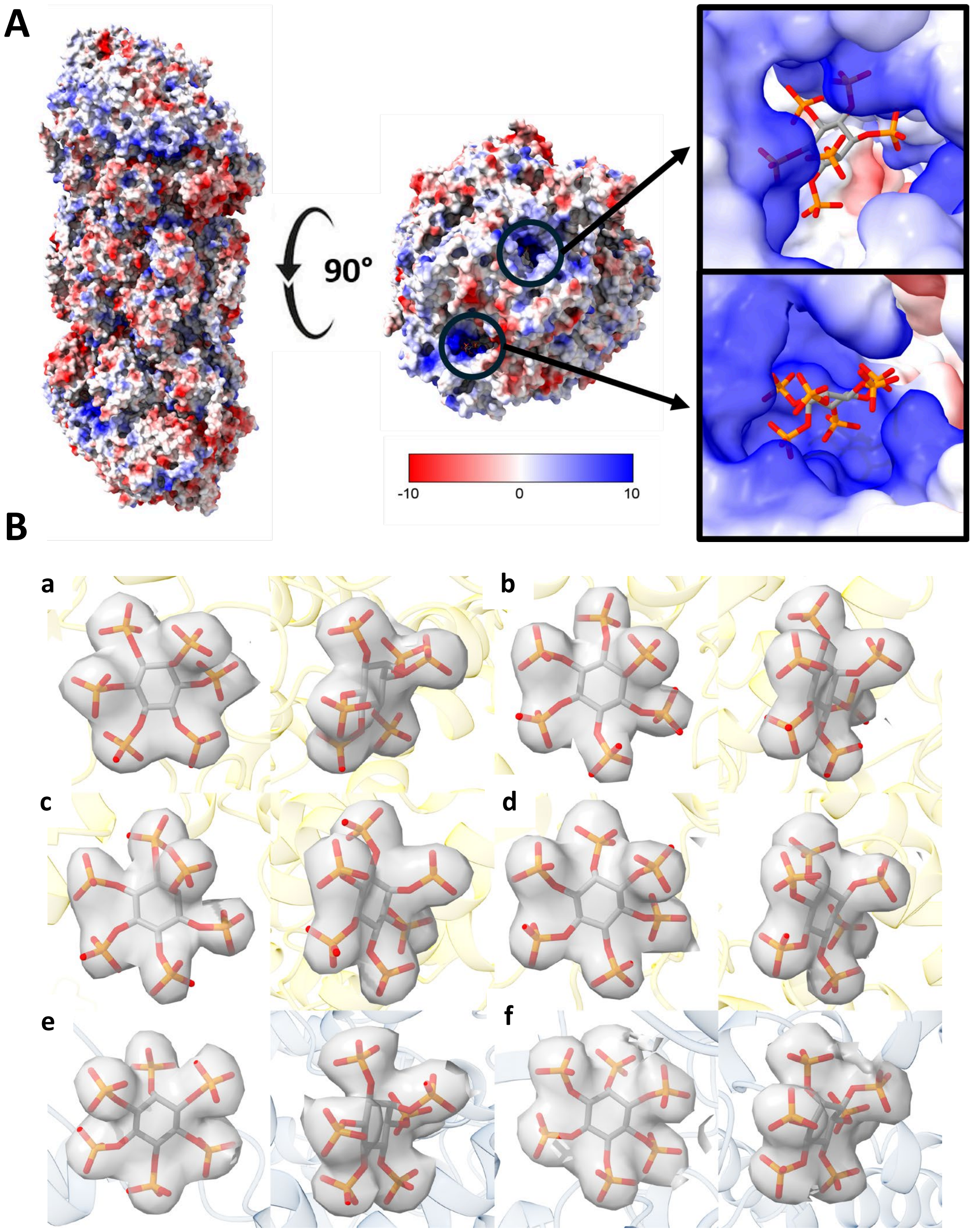

Supplementary Figure 5: **Phytic acid binds to the i20S-PA200 complex.** (**A**) Front and top views of the singly-capped i20S-PA200 complex showing the binding of two IP6 molecules in the positively charged grooves. (**B**) Inositol hexakisphosphate displayed as map fit with a stick model for doubly-capped i20S-PA200 complex (a-d) and singly-capped i20S-PA200 complex (e-f).

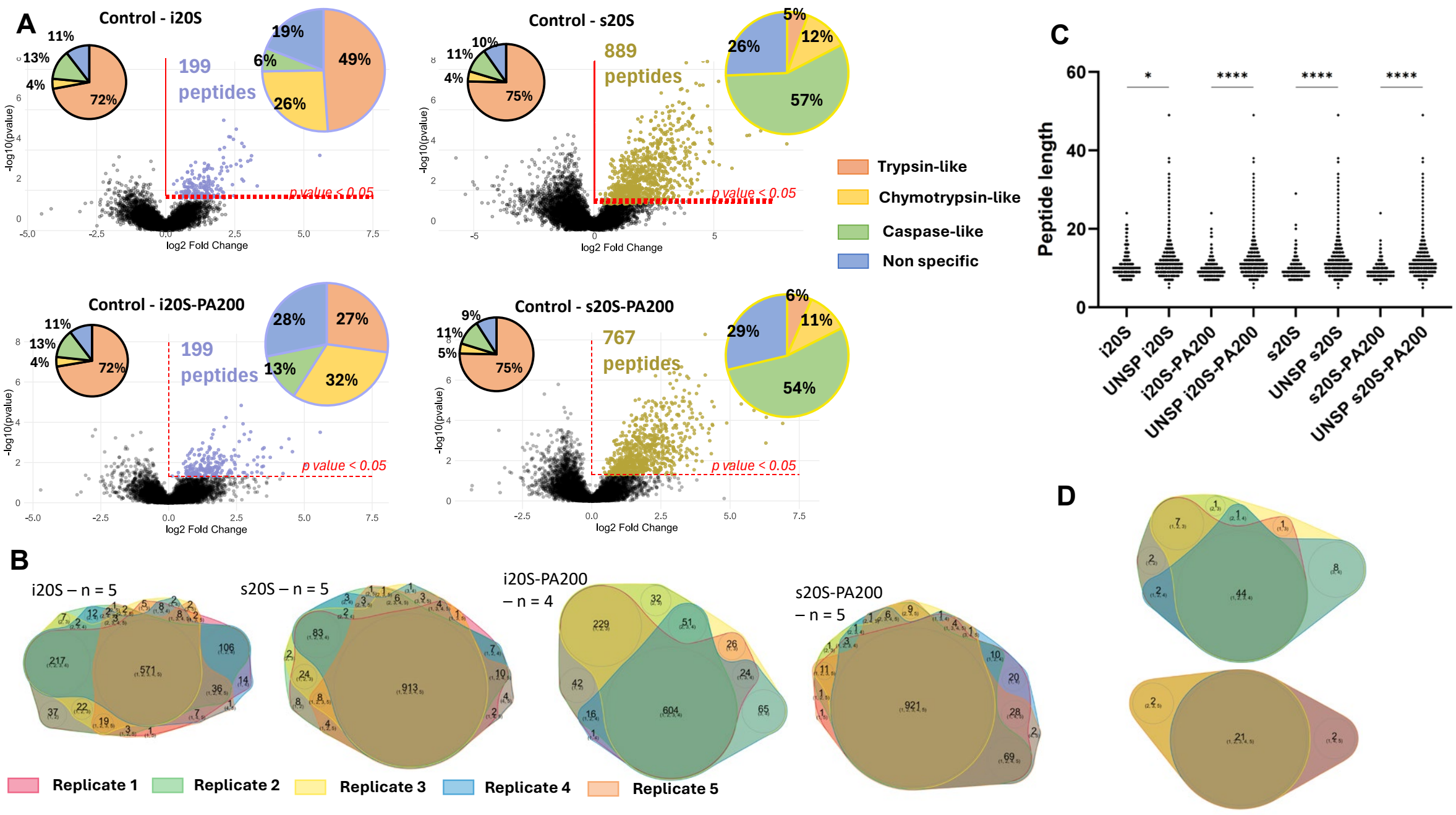

Supplementary Figure 6: **(A)** Volcano plots highlighting the peptides specifically generated by the different forms of proteasome (i20S and i20S-PA200 displayed as blue dots, s20S and s20S-PA200 displayed as yellow dots) compared to non-proteasomal peptides under control conditions (non-variant or under-represented peptides as black dots), and pie charts showing the C-terminal residue analyses of these specific proteasomal peptides (pie charts on the right of each volcano plot) compared to unspecific peptides (pie charts on the left of each volcano plot). The dashed red lines indicate the thresholds applied (FC and *p* value; a t-test (p-value Benjamini-Hochberg correction) was applied a two-sided t-test was used, n = 4. Peptidomics source data were deposited with the dataset identifier PXD075459 and C-terminal residue analysis source data are provided as a Source Data file. **(B)** Venn diagram highlighting the identification overlap across replicates of proteasomal peptides derived from free i20S (n = 5), s20S (n = 5), PA200-activated i20S (n = 4), and PA200-activated s20S (n = 5). **(C)** Analysis of the length distributions of the peptides identified as proteasome-derived peptides of i20S, i20S-PA200, s20S, s20S-PA200, or as unspecific peptides (detected as under-represented or non-variant in the different pairwise comparisons with the control in panel A). A one-way ANOVA test was used for the pair-wise comparisons. ns: no significance, *: *p*-value<0.05, **: *p*-value<0.01, ***: *p*-value<0.001, and ****: *p*-value<0.0001. Source data are provided as a Source Data file. **(D)** Venn diagrams showing the identification overlap across replicates of the peptides specifically generated by the PA200-mediated activation of i20S (top) and s20S (bottom).

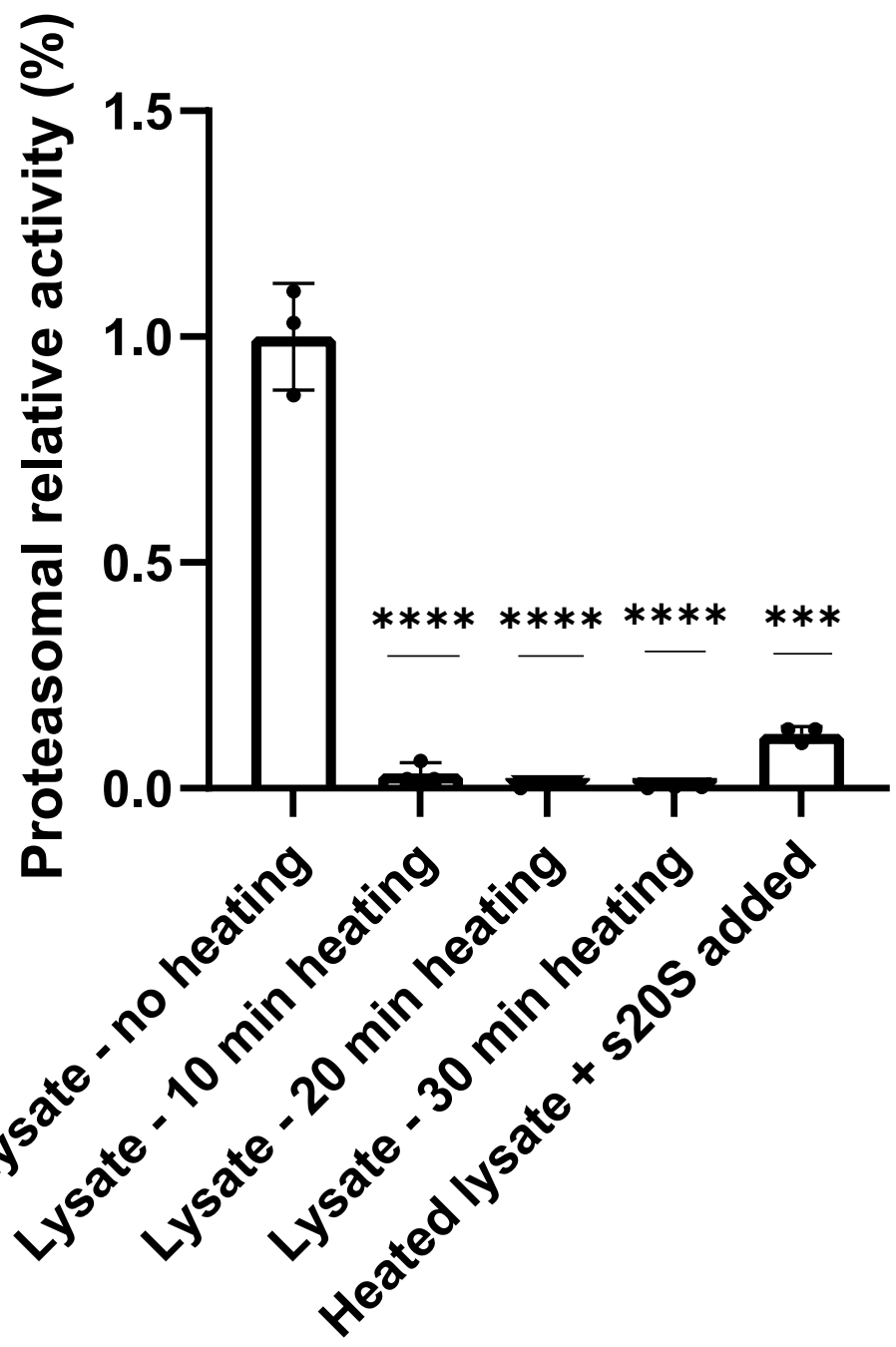

Supplementary Figure 7: Chymotrypsin-like proteasome activity was measured using the LLVY fluorogenic peptide in HEK-EBNA cell lysates, either untreated or subjected to heat treatment at 95 °C for 10, 20, or 30 minutes. Three independent replicates were analyzed (n=3). As a positive control, 5 µg of purified proteasome was added to the 5 minutes-heated lysate. The untreated lysate was used as a reference to calculate the percentage of residual activity under each condition. Statistical significance was assessed using a one-way ANOVA, with pairwise comparisons between each condition and the reference (***: *p*-value < 0.001; ****: *p*-value < 0.0001). Data are shown as mean values, the standard deviations, and the individual values of each replicate (n=3 independent replicates). Source data are provided as a Source Data file.

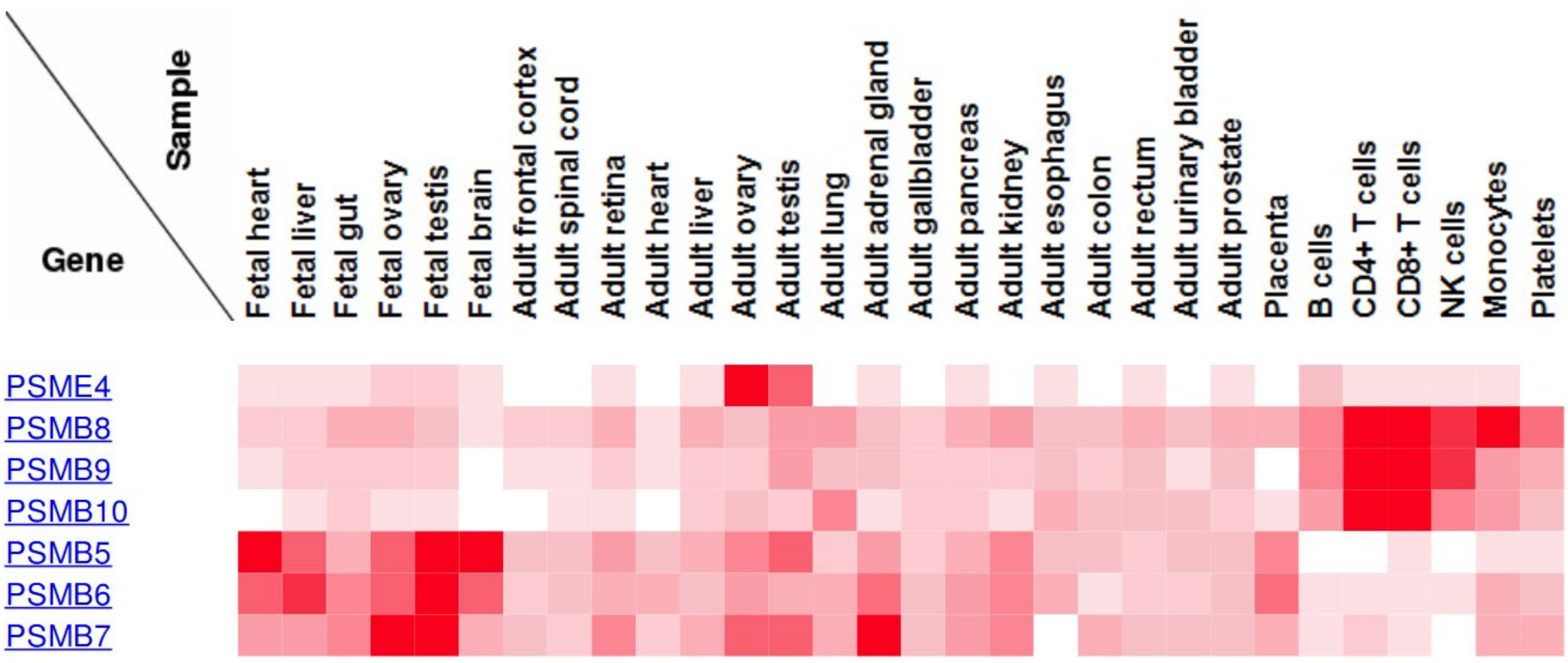

Supplementary Figure 8: Mapping of the protein expression of PA200 (PSME4) and the catalytic subunits of the i20S (β1i, β2i and β5i, i.e. PSMB8-10, respectively) and s20S (β1, β2 and β5, i.e. PSMB5-7, respectively). Data was retrieved from[32].

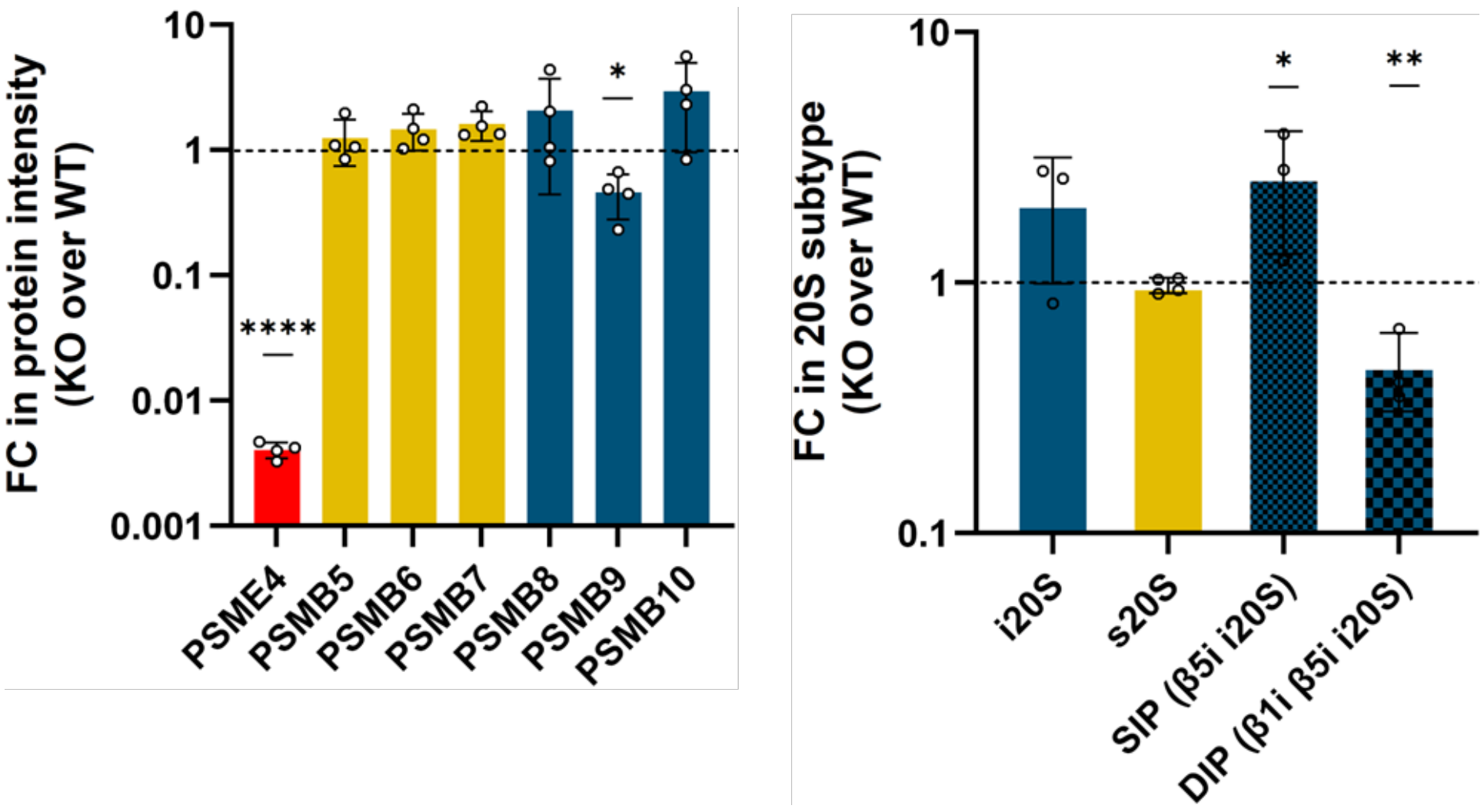

Supplementary Figure 9: Depletion of PA200 reduces i20S expression and changes the composition of 20S subtypes in lung cancer cells. Two different WT and PA200 KO clones of A549 and H1299 were used for proteasome immunopurification using the MCP21 antibody. The expression of PA200 and catalytic 20S subunits were quantified by LC MS/MS. For each protein, the log10-fold change of the intensities in PA200 KO over WT was calculated. In this data set, the stoichiometry of each 20S subtype was calculated (as in[38]) and for each 20S subtype, the fold change of the stoichiometry in the PA200 KO over WT was calculated. Bars show quantification of expression relative to average WT levels set to 1 (two-sided paired t-test). Data are shown as mean values, the standard deviations, and the individual values of each replicate (n=4 independent replicates). *: *p*-value<0.05, **: *p*-value<0.01. Source data are provided as a Source Data file.

Supplementary Table 1: Cryo-EM data collection, refinement and validation statistics

	Human 20S immunoproteasome in complex with two PA200 activators (EMD-58410) (PDB 31HA)	Human 20S immunoproteasome in complex with PA200 activator (EMD-58411) (PDB 31HB)
**Data collection and processing**		
Magnification	29000	29000
Voltage (kV)	300	300
Electron exposure (e–/Å^2)	60	60
Defocus range (μm)	1.5 - 2.5	1.5 - 2.5
Pixel size (Å)	0.95	0.95
Symmetry imposed	C1	C1
Initial particle images (no.)	607937	607937
Final particle images (no.)	187565	225565
Map resolution (Å)	2.89	2.85
FSC threshold	0.143	0.143
Map resolution range (Å)	2.0 – 3.0	2.0 – 3.0
**Refinement**		
Initial model used (PDB code)	6AVO, 6REY	6AVO, 6REY
Model resolution (Å)	2.89	2.85
FSC threshold	0.143	0.143
Model resolution range (Å)	2.0 – 3.0	2.0 – 3.0
Map sharpening *B* factor (Å^2)	-88.4	-90.4
Model composition		
Non-hydrogen atoms	73485	60373
Protein residues	9645	7935
Ligands	4	2
B factors (Å^2)		
Protein	95.54	103.46
Ligand	141.84	132.12
R.m.s. deviations		
Bond lengths (Å)	0.004	0.005
Bond angles (°)	1.013	1.019
Validation		
MolProbity score	1.63	1.63
Clashscore	4.69	4.38
Poor rotamers (%)	1.55	1.57
Ramachandran plot		
Favored (%)	96.34	96.08
Allowed (%)	3.55	3.59
Disallowed (%)	0.12	0.33

Supplementary Table 2: RMSD of the main catalytic residues between the catalytic cores (i20S and s20S) alone and bound to PA200.

	**i20S (6AVO) vs PA200-i20S-PA200**
**β1/β1i**	RMSD between 24 atom pairs is 0.984
**β2/β2i**	RMSD between 24 atom pairs is 0.573
**β5/β5i**	RMSD between 24 atom pairs is 0.722
	**s20S (7NAN) vs PA200-s20S-PA200 (6REY)**
**β1/β1i**	RMSD between 24 atom pairs is 0.821
**β2/β2i**	RMSD between 24 atom pairs is 0.362
**β5/β5i**	RMSD between 24 atom pairs is 0.426

Supplementary Table 3: Slope-derived rates from raw fluorescent data corresponding to Figure 4.

Figure 4A

Sample	molar ratio	LLVY	Chymotryptic-like _ slope-derived rates				LRR	Tryptic-like _ slope-derived rates				LLE	Caspase-like_ slope-derived rates			
		1	2	3	4	5	1	2	3	4	5	1	2	3	4	5
s20S+PA200	1:0	100,47	103,36	90,47	103,88	101,54	56,52	63,27	49,41	59,97	58,84	772,99	746,82	781,76	694,33	728,54
	1:2	277,59	269,42	248,99	248,72	208,06	117,24	107,82	109,94	112,88	112,11	1345,46	1371,14	1350,73	1306,38	1277,97
	1:4	373,11	356,96	356,28	314,99	250,86	100,31	111,25	98,36	104,36	105,79	1323,41	1248,55	1246,45	1252,49	1166,61
	1:8	392,22	400,57	416,32	387,91	481,81	140,8	149,7	126,1	143,01	142,76	2187,55	2054,87	2008,92	1991,68	1950,92
i20S+PA200	1:0	126,85	115,86	112,57	119,87	70,08	47,32	56,03	58,57	57,84	62,51	12,08	18,38	9,82	8,13	17,72
	1:2	438,35	398,14	416,40	440,20	336,17	130,6	145,13	143,55	149,53	151,95	43,49	45,83	51,07	38,67	44,95
	1:4	521,18	493,65	515,84	531,49	502,47	153,87	164,33	171,72	168,87	171,08	76,32	76,23	82,03	70,83	73,23
	1:8	636,75	629,83	611,75	578,57	742,42	197,37		211	213,1	187,7	116,12	115,73	125,4	114,28	110,15

Figure 4B

Sample	LLVY	Slope-derived rates			ANW	Slope-derived rates			PAL	Slope-derived rates			LRR	Tryptic-like _ slope-derived rates		
	1	2	3	4	1	2	3	4	1	2	3	4	1	2	3	4
i20S	20,36	18,29	17,67	17,03	33,28	33,96	31,31	33,61	55,59	57,30	57,00	56,52	39,57	38,17	39,62	31,18
i20S + PA200	145,25	126,34	124,06	135,94	163,09	148,61	149,03	170,23	154,42	181,63	158,18	160,80	80,83	86,01	85,91	78,72
i20S + IP6	19,76	16,59	14,92	15,00	24,46	25,77	26,25	30,14	50,80	55,36	56,22	58,64	40,90	42,05	44,71	40,65
i20S + PA200 + IP6	127,63	131,07	121,91	112,41	247,26	208,54	253,20	251,74	266,20	267,51	259,85	249,09	191,85	198,64	190,37	202,05

Figure 4C

Sample	LLVY	Slope-derived rates			ANW	Slope-derived rates			PAL	Slope-derived rates			LRR	Tryptic-like _ slope-derived rates		
	1	2	3	4	1	2	3	4	1	2	3	4	1	2	3	4
s20S	50,40	48,33	46,58	45,26	38,29	40,77	40,80	40,32	67,11	73,61	73,29	72,93	77,97	89,34	78,22	89,42
s20S + PA200	100,88	97,43	98,98	99,37	43,20	38,81	43,54	47,12	83,76	87,23	91,08	80,87	149,62	140,72	134,63	135,07
s20S + IP6	54,70	50,67	49,47	47,93	33,30	30,83	30,94	31,56	54,98	59,87	61,77	61,60	84,77	85,73	82,96	86,87
s20S + PA200 + IP6	87,35	86,41	82,81	88,53	49,32	45,67	46,27	46,76	85,50	88,95	84,36	90,23	201,58	177,46	200,20	182,80
