# Supplementary material for: PA200 modulates immunoproteasome structure and activity
Source: Nat Commun. 2026 Aug 11;17:8175. doi: 10.1038/s41467-026-75325-w (PMC13463089; doi:10.1038/s41467-026-75325-w)
Supplement: Supplementary file 2 [file 41467_2026_75325_MOESM2_ESM.pdf]

Description of Additional Supplementary Files

File name: Supplementary Movie 1

Description: Morph animation from the s20S-PA200 structure (yellow) to the i20S-PA200 structure (blue) showing that the bending of the i20S-PA200 results in a slight but significant decompaction of the whole complex.
