# Supplementary material for: PA200 modulates immunoproteasome structure and activity
Source: Nat Commun. 2026 Aug 11;17:8175. doi: 10.1038/s41467-026-75325-w (PMC13463089; doi:10.1038/s41467-026-75325-w)
Supplement: Supplementary file 4 [file 41467_2026_75325_MOESM5_ESM.pdf]

nature portfolio

Peer Review File

Uncovering the Role of Proteasome Activator PA200 in Regulating the Immunoproteasome

Corresponding Author: Professor Marie Pierre Bousquet

Parts of this Peer Review File have been redacted as indicated to remove third-party material. Unpublished, confidential data has been redacted as indicated.

This file contains all reviewer reports in order by version, followed by all author rebuttals in order by version.

Attachments originally included by the reviewers as part of their assessment can be found at the end of this file.

Version 0:

Reviewer comments:

Reviewer #1

(Remarks to the Author)
The study of Zivkovic and colleagues addresses an interesting topic such as the role that PA200 (PSME4) could have in the regulation of proteasome isoforms activity and expression. Some of the authors were directly involved in other recent papers on the same topic, which have been published in prestigious journals. I have some major concerns that are in part related to what was published in Javitt et al., Nat Cancer 2023 compared to the content of this manuscript, bearing in mind that several scientists were authors in both studies.
Major concerns:
1. I appreciate that the authors obtained a new cryo-EM structure of i20S bound to one or two PA200 molecules and that this structure shows singular characteristics such as the general bend of the i20S-PA200 not seen in any other structure of proteasome activators bound to 20S. I am not an expert in cry-EM but as a rule of thumb I would have expected the authors generating in parallel cryo-EM structures of also s20S-PA200 and ideally even PA28ab-i20S. Can they authors exclude that what they see is an artefact or something that previous studies did not grasp for other proteasome regulators?
2. The authors here claims that the genetic depletion of PA200 in A549 lead to a significant reduction of PSMB9. However, in Javitt et al. it is stated that, upon genetic depletion of PA200 in A549, 'none of the genes with altered expression had known associations with tumour-immune interaction' and 'we found only a mild impact on gene expression'.
3. The authors measured proteasome activity using standard short fluorogenic substrates, which could be used to test proteasome activity but might not represent the real degradation of substrates by proteasomes in vitro and in cellular systems. Based on what I see in Fig. 4, PA200 increases the degradation of all 3 substrates, with an effect more pronounced for i20S han s20S. However, in Javitt et al. it is stated that, 'abundance of PSME4 was positively correlated with the caspase- and chymotryptic-like cleavage activities of the proteasome and negatively correlated with tryptic-like cleavage (Fig. 3c,d and Extended Data). Also in Javitt et al., it is stated that 'We found that the caspase-like (β1) activity of the constitutive proteasome increased (Fig. 3g and Extended Data Fig. 7d), whereas the tryptic-like (β2) activity decreased, upon PSME4 supplementation (Fig. 3h and Extended Data Fig. 7e). By contrast, when examining the effect of PSME4 on immunoproteasome-associated activities, we found that all were inhibited (β1i, β2i and β5i). Honestly, I struggle to reconcile these results with what the authors propose in the submitted manuscript.
4. A way to clarify these discrepancies could be performing digestions assays of entire proteins using i20S and different proteasome activators, and measure the peptide product via MS and the protein degradation rate via SDS-gel.
5.
Minor comments:
1. I think there is a problem with the reference enumeration between lines 80 and 88. It jumps from 10 to 15 and then back to 13 and later 11,12.
2. I do not see the advantage of citing a review 21 years old (ref 1). Many updated reviews have been published since then.

Reviewer #2

(Remarks to the Author)

The authors present a combined structural and biochemical study of PA200 binding to 20S. Overall, the study is very detailed and thorough, the figures and text clear, and a clear case is made as to the key advance presented. My expertise is in mass photometry, so I will focus my comments on that (small aspect) of the manuscript, although having read the entire paper, it seems a novel and clear piece of work overall that deserves publication. I have two comments that the authors may want to address prior to publication.

Major
I am not sure I really understand how the KDs were calculated and it is not clear to me that it was done correctly form a thermodynamic perspective. I have attached a document for the authors to consider.

Minor
Abstract
The way it is written in the past tense - it really is not clear what was done in this work, and what was done by the authors/others in the past. A simple 'Here, …' would help the reader to differentiate.

Line 99 KDs - enhanced formation of PA200 - enhanced how? Cooperative enhanced or enhanced for i20s compared to s20s? Not clear

Reviewer #3

(Remarks to the Author)
Summary:
This study explores the structural and functional interplay between PA200 and the immunoproteasome (i20S). It reveals mechanisms that distinguish its regulation from that of the standard proteasome (s20S). Using cryo-EM, the authors show that binding of a single PA200 particle induces long-range conformational rearrangements in the i20S core, thereby facilitating subsequent engagement of a second PA200 and enhancing catalytic activity. Mass photometry supports increased occupancy and affinity of PA200 for i20S over s20S, while activity assays demonstrate more robust activation of i20S, especially in the presence of inositol hexakisphosphate (IP6). Proteomic and pull-down data reinforce the preferential binding of PA200 to i20S in tissues, and gene expression studies suggest a possible regulatory role for PA200 in shaping i20S composition.

Overall, this is a solid manuscript, but not a terribly exciting one. Some of the results are interesting, but I am not convinced the manuscript as a whole measure up to the level required for publication in Nat Comm. Perhaps a more specialized journal would be more appropriate.

Major Comments:

1. The structural data are the most interesting aspect of the manuscript. However, the analysis feels underdeveloped. The claim of a "bent" conformation in the singly- and doubly-capped i20S-PA200 complexes would benefit from more rigorous analysis. Currently, the structural figures do not convincingly convey this claim. Is this a true bend or a translational offset? The authors should attempt alternative alignment strategies and clearer visualization (e.g., angular displacement measurements, movies, or vector displacement plots) would help clarify this. The proposed allosteric effects, particularly on the opposite α-ring, are central to the manuscript and deserve a more thorough, quantitative description.

2. The mass photometry experiments suggest differential binding affinity of PA200 to i20S and s20S, but the quantitative conclusions are overstated. The fits are poorly constrained and clearly underestimate uncertainty. Mass photometry is best viewed as qualitative or semi-quantitative; definitive conclusions about dissociation constants require orthogonal validation, e.g., surface plasmon resonance or isothermal titration calorimetry. If the authors wish to report Kd values, confidence intervals (obtained properly) and goodness-of-fit metrics should be included in the main figures, not only the supplement.

3. Kinetic characterization of PA200 binding would benefit from a more complete kinetic characterization of PA200 binding. Time-course measurements or other kinetic approaches would allow for more precise comparisons between the i20S and s20S binding behavior, rather than relying on endpoint titrations and bar plots.

4. The activity assays provide useful functional insight, but the presentation is limited to relative values and bar plots. Raw fluorescence data or slope-derived rates should be shown in supplementary figures to allow assessment of assay quality. It would also be helpful to present basal activity levels for i20S and s20S side-by-side, especially in Figure 4B and 4C. This would better contextualize the extent of activation by PA200 and IP6.

5. The assignment of IP6 in the density maps is presented with little analytical support. Is this modeling based solely on fitting to positive density? If so, this should be made explicit, and the limitations acknowledged. Additionally, the rationale for using s20S basal activity as the reference in all comparisons is unclear. For clarity, activation effects should be expressed relative to the appropriate unbound control for each proteasome type.

6. The evidence suggesting that PA200 regulates i20S subunit expression is intriguing but not fully convincing. The error bars are large, and the mechanistic basis is unexplored. Additional transcriptomic or pathway analyses would help strengthen this section. At minimum, the authors should acknowledge the limitations of these findings and avoid

overinterpreting their significance.

Minor Comments:
1. Line 112: The term "bend" to describe the i20S-PA200 conformation is not clearly supported by the figures. Consider rewording or providing clearer visual evidence (e.g., morph animations or angle measurements). The figure need to be improved and polished.

2. Figure 1: Consider including a comparison of apo i20S and s20S structures to highlight differences prior to PA200 binding. RMSD comparisons could be informative.

3. Figure 2A: While peripheral helices show changes, alterations in the axial channel are less apparent. Quantitative pore measurements or cross-sectional diagrams would help clarify these structural changes.

4. Line 140: The discussion of active-site rearrangements is vague. A structural figure showing local RMSD of active site residues would help.

5. Figure 6A, D, E: Clarify the meaning of the different colored bars in the graphs. This is not currently evident from the figure legend.

6. Line 59: Replace “macromolecular machinery” with “macromolecular machine.”

7. Line 64: “Proteome” should read “proteasome.”

8. Line 163: Consider revising “further away” to “furthest away.”

9. Line 381: “Majority of tissues doesn’t” should read “the majority of tissues do not.”

10. Line 66: Remove “actually”. It is unnecessary.

11. Line 83: Consider replacing citations from MDPI journal with peer-reviewed primary literature published in reputable journals. Additionally, citing numerous studies while simultaneously referring to PA200 as “enigmatic” iscontradictory.

12. Line 194: “It was suggested” should be attributed or rephrased for clarity and specificity.

Reviewer #4

(Remarks to the Author)
I co-reviewed this manuscript with one of the reviewers who provided the listed reports. This is part of the Nature Communications initiative to facilitate training in peer review and to provide appropriate recognition for Early Career Researchers who co-review manuscripts.

Version 1:

Reviewer comments:

Reviewer #1

(Remarks to the Author)
Zivkovic and colleagues addressed some of the concerns that I proposed in my previous reviewing, for example by clarifying and presenting new evidence for my point 1 (cryo-EM comparison with s20S-PA200).
Unfortunately, I am not fully convinced by the reply to my points 2 and 3-4. I am sorry to keep referring to in Javitt et al., Nat Cancer 2023 but the prominent authorship of S. Meiners in both studies request an extra effort in generating a comprehensive picture that provide a shaped knowledge to the audience. Something that could be in textbooks in the future.
Re the two points:

2. Difference in the exp design can explain a different outcome and I can live with a discrepancy between exp. systems that is coupled to ‘in system PA200 one does not affect much, in system 2 does affect i20S expression’. The revised discussion addresses this, although it leave me with an unclear understanding if PA200 indeed affect i20S expression.

3-4. The authors have an explanation of why their results with using short fluorogenic peptides (SFPs) +/- PA200 generated contradicting results than Javitt et al., Nat Cancer 2023.
I agree that the exp performed with purified s/i20S + PA200 could allow a clear investigation of what 20S+PA200 does. However, the contradiction with the results that were published in Javitt et al., Nat Cancer 2023 requires more investigation. Although the authors performed an assay with bovine histones, they reported that only in the reply to reviewers. That experiment, which was indeed what I suggested, did not show any significant difference between s20S and i20S. The justification that acetylated histones are the only commercially available for this assay does not stand. There have been several papers that successfully used proteins for the degradation by s20S and i20S in the last two decades. I am confident that many of those proteins could then also be well degraded by 20S+PA200.

I think this experiment is pivotal otherwise I remain with a significant doubt and I am not convinced that the title of the manuscript is really supported by the data.
The reality is that the SFP exp do not confirm what was published by Javitt et al., Nat Cancer 2023. If the authors could perform a new set of exp using proteins and measuring the peptide products, they could also significantly help in the understanding the impact of PA200 on s20S vs i20S degradation. With this, the manuscript would be improved. Without, I do not understand what the changes identified by cryo-EM lead to.

Reviewer #2

(Remarks to the Author)
The authors have carefully considered my previous comments on the mass photometry (MP) experiments and have revised the relevant sections accordingly. I will address only to their response to my previous comments. I agree with their conclusion that the results support preferential binding of PA200 to i20S over s20S, and I also recognise that precise affinity determination is not the main focus of their paper. Nevertheless, I think that the authors should consider the points below regarding their quantification methodology and decide whether reporting numerical values is significant for their publication.
The data demonstrate higher PA200 occupancy on i20S, as the authors state. However, the reported fitted dissociation constants indicate lower k_D values for s20S than for i20S, which is inconsistent with the observed and stated preferential binding. This discrepancy may result from the fitting approach applied. If the numerical values are to be reported and are significant, the authors should clarify or revisit the fitting strategy to ensure consistency or explicitly discuss this issue.
Clarification of the MP equilibrium calculation. As I now understand the procedure, the samples were incubated at 120 nM 20S with 0 - 1.8 μM PA200 for 2 hours, then diluted six times immediately prior to the MP measurement. In my view, two distinct assumptions may lead to two valid approaches for calculating the interaction strength that will result in different values:
a. Rapid re-equilibration after dilution. If the authors assume that the system re-equilibrates rapidly following the dilution, then the equilibrium analysis should indeed use the post-dilution concentrations (20 nM), however, this assumption should be explicitly stated.
b. Negligible redistribution during measurement. If the measurement was performed immediately after dilution and the distribution is assumed (or shown) not to change during this short interval, then the incubation concentrations (120 nM 20S) should be used for the equilibrium calculation. The authors should clearly/justify which assumption applies.
Potential influence of PA200 oligomerisation. A plausible explanation for the early plateau in the binding curves is PA200 dimerisation. Depending on the dimerisation constant, this could considerably flatten the concentration increase of free monomeric PA200 and thereby influence the effective ligand concentration on the x-axis in Fig. 3C. Also, regardless of this cause, only the concentration of the free monomeric PA200 should be used for calculating binding constants, as this is the species relevant to the binding interaction.
Given these considerations, I believe the authors should decide the most appropriate representation of their data and, in particular, if numerical values are reported these should be referred to as apparent K_D.

Reviewer #3

(Remarks to the Author)
The revision has improved the manuscript in several ways: the biochemical assays are more carefully documented, over-interpretation of the PA200/i20S expression link has been curtailed, and some of the more ambitious claims based on mass photometry have been softened. At the same time, the structural “bend” is only somewhat better supported (the new included movie is not convincing), and the manuscript remains weakest is the mass photometry–based thermodynamic analysis. The authors have clarified concentrations, re-plotted against free ligand, and added confidence intervals and goodness of fit. All of these are welcome. They have also backed away from strong claims about cooperativity and precise Kd values.
Nonetheless, the fundamental issues are not resolved: simple equilibrium calculations and Hill fits yield incompatible Kd estimates for the same data; the unusually low maximal occupancies and the behaviour of singly-bound species are not convincingly explained. There is no orthogonal biophysical method to anchor the binding parameters. The authors explicitly state that they do not understand why the analytical and fitted Kd values disagree. That is not a satisfactory thermodynamic reconciliation, and it means the mass photometry data should, at best, be treated as semi-quantitative support for a higher fraction of doubly capped i20S–PA200 under the chosen conditions, rather than as a rigourous determination of binding affinities or cooperativity. Taken together, the limitations in the depth and robustness of the structural and thermodynamic analysis remain. I stand my original assessment that the manuscript does not clear the bar for Nature Communication.

Reviewer #4

(Remarks to the Author)
I co-reviewed this manuscript with one of the reviewers who provided the listed reports. This is part of the Nature Communications initiative to facilitate training in peer review and to provide appropriate recognition for Early Career Researchers who co-review manuscripts.

Version 2:

Reviewer comments:

Reviewer #1

(Remarks to the Author)
Zivkovic and colleagues made an appreciated attempt to address my two concerns of the previous revised manuscript. I am sorry to say that I still have concerns. I hope that they can help to improve the manuscript.

Concern 1. I followed the explanation of the authors on the question: does PA200 indeed affect i20S expression? The statement that it depends on the cell state and the level of expression of both PA200 and immunoproteasome, bearing in mind that the conclusion is derived from 2 cell types is questionable. I think that the emphasis in the title and abstract and many sentences on this topic in the manuscript should be turned down. If in two cell background the authors see different things, either they increase the cell systems investigated or the reduce the emphasis of the claims.

Concern 2. The authors did not perform the experiment that I suggested but another one similar, which also has the potential of providing more information than the experiment that I proposed. Indeed, they performed a digestion of the entire cell lysate after heat denaturation using s20S, i20S +/- PA200. And performed DIA peptidomics on the samples after 1 h of processing by 20S-PA200. The idea is good. N=5 is a good exp. design. The presentation of the data significantly is less appreciated. For example:
a. The authors do not show how many peptides are present in absence of proteasome (background). This does not let me appreciate the statistical power of the analysis and have an evaluation of the biological meaning of the output.
b. The authors do not do a comparison of many other basic features of the peptide products (peptide length distribution, protein origin, feature of the protein origin) and they rather focus only on the C-termini grouping.
c. Some controls are just reported but not shown (e.g., 'Proteasome substrate-based activity assays with the LLVY fluorogenic peptide were used to verify that the endogenous proteasome activity was completely abolished by the heating step.'. Not shown.
d. I read: 'A large overlap of at least 1000 proteasome derived peptides was identified in at least 3 replicates across the different replicates (n = 4 or 5, Figure S6A-B)'. I do not understand what this means. Are 1000 peptides shared among all 4 conditions? How many peptides are identified per condition? The authors could decide a strategy to define a peptide as 'identified' (e.g., identified in 2 biol. Replicates) and then do a deeper analysis of their features (see above).
e. I understand that 21 peptides are identified in all biological replicates of s20S + PA200 compared to s20S (fig. S6C). Is this part of the 921 peptides identified in all replicates of s20S+PA200? If so, it means that the authors are building an analysis of the difference in proteasome activity based on a 2% of peptides? How is this biologically relevant? Similarly, for i20S.
f. In Fig. 5A, C and Fig. S6B, I do not understand what is shown. Are they all peptides that have been identified? Is the analysis quantitative (as I would expect from the volcano plot and the DIA MS)? What is the relation of the peptides identified only in a condition and the quantitative analysis shown in the volcano plot? Are the T-like, CT-like and caspase-like activities in agreement between these two groups of peptides (those uniquely identified and those over-represented)?
g. In Fig. 5A,C and Fig. S6B, are the C-terminus groups different between those over-represented (top right; already analysed) and those under-represented (top left; not analysed)? Because I would expect an opposite effect on the right and the left of each volcano plot, according to the author hypothesis. This analysis could strengthen the conclusion of the authors that, as explained above, might be based on 2-6% of the peptides. This is quite a small portion of all peptides identified.

Minor:
I think Fig. 5C caption is missing

Reviewer #2

(Remarks to the Author)
The authors have done an excellent job, I have no further comments.

Reviewer #3

(Remarks to the Author)
The authors have satisfactorily addressed my comments. I believe the manuscript is ready for publication.

Reviewer #4

(Remarks to the Author)
I co-reviewed this manuscript with one of the reviewers who provided the listed reports. This is part of the Nature Communications initiative to facilitate training in peer review and to provide appropriate recognition for Early Career Researchers who co-review manuscripts.

Version 3:

Reviewer comments:

Reviewer #1

(Remarks to the Author)
Zivkovic and colleagues addressed all my criticisms and I think the manuscript is significantly improved.

Open Access This Peer Review File is licensed under a Creative Commons Attribution 4.0 International License, which permits use, sharing, adaptation, distribution and reproduction in any medium or format, as long as you give appropriate credit to the original author(s) and the source, provide a link to the Creative Commons license, and indicate if changes were made.
In cases where reviewers are anonymous, credit should be given to 'Anonymous Referee' and the source.
The images or other third party material in this Peer Review File are included in the article's Creative Commons license, unless indicated otherwise in a credit line to the material. If material is not included in the article's Creative Commons license and your intended use is not permitted by statutory regulation or exceeds the permitted use, you will need to obtain permission directly from the copyright holder.
To view a copy of this license, visit https://creativecommons.org/licenses/by/4.0/

Reviewer #1 (Remarks to the Author)

The study of Zivkovic and colleagues addresses an interesting topic such as the role that PA200 (PSME4) could have in the regulation of proteasome isoforms activity and expression. Some of the authors were directly involved in other recent papers on the same topic, which have been published in prestigious journals. I have some major concerns that are in part related to what was published in Javitt et al., Nat Cancer 2023 compared to the content of this manuscript, bearing in mind that several scientists were authors in both studies.

Major concerns:
1. I appreciate that the authors obtained a new cryo-EM structure of i20S bound to one or two PA200 molecules and that this structure shows singular characteristics such as the general bend of the i20S-PA200 not seen in any other structure of proteasome activators bound to 20S. I am not an expert in cry-EM but as a rule of thumb I would have expected the authors generating in parallel cryo-EM structures of also s20S-PA200 and ideally even PA28ab-i20S. Can they authors exclude that what they see is an artefact or something that previous studies did not grasp for other proteasome regulators?

R1: We thank the reviewer for this very helpful comment. Indeed, we were able to obtain cryo-EM structures from singly- and doubly-capped s20S-PA200 complexes with a 3.7 Å resolution but did not work on cryo-EM structures with PA28αβ, as this was not the focus of our study.
To rule out the possibility that the bend we observe in i20S-PA200 is an artefact, we solved an in-house cryo-EM structure of s20S-PA200 processed similar to i20S–PA200 complexes and using the same pipeline. We did not deposit this s20S-PA200 model because it is essentially indistinguishable from the previously published s20S-PA200 structure (6REY.pdb). We compared the publicly available PA200-s20S-PA200 (6REY.pdb) to our in-house s20S-PA200 and found that the structures superimpose very well, with low RMSD. We included this comparison into our newly revised Figure 1 (Figure 1B, dashed orange line). Importantly, no global bend is detected (Figure S2). When we compared our i20S-PA200 and s20S-PA200 complexes, however, we observed the bend that is the subject of this manuscript. Together, these results indicate that the observed tilting in the i20S-PA200 structure is not an artefact, but an i20S-specific feature of PA200 engagement. We also tested whether this bending was observed upon binding of other 20S-activator complexes to the i20S, e.g. such as the previously published cryo EM structure of the bovine i20S doubly capped with two human PA28αβ (DOI: 10.1038/s41467-021-21028-3, 7DRW.pdb / EMD-30828). Even though the catalytic cores of these two complexes belong to two different species (human and bovine) and despite the fact that the main chain of the bovine i20S could only be traced at 4.1 Å, we compared the PA28αβ-i20S-PA28αβ vs. PA200-i20S-PA200 structures, and saw a very similar RMSD increase across all α- and β-ring (revised Figure 1B,

dashed yellow line) as when comparing the PA200-s20S-PA200 with our PA200-i20S-PA200, confirming our finding that the bending of the structure is a specific feature of PA200 bound to i20S. Indeed, if the binding of PA28αβ was inducing such binding, the RMSD values with our structure would be close to zero. We provide the revised Figure 1B for the convenience of the reviewer below. The text for the results part has been changed accordingly to: **"Here, we obtained high resolution structures for both singly- and doubly-capped i20S-PA200 complexes (2.85 Å and 2.89 Å resolution, respectively). Although the doubly-capped structure of the i20S-PA200 complex seems similar to s20S-PA20020-23 (Figure 1A, see Figure S1 for details on 3D classification), the structure shows a remarkable "bend" of the entire complex (Figure 1B-C) which has not been observed in proteasome structures before. The bend propagates from the proximal PA200 molecule, which was superimposed with the corresponding PA200 in recently solved s20S-PA200 structure (6REY.pdb) with minimal Root Mean Square Deviation (RMSD~1.7 Å; Figure 1B, black dashed line). From that region, the structure of i20S-PA200 begins to "bend", as indicated by the gradual increase in calculated RMSDs to its maximal value in the distal PA200 (RMSD ~6.8 Å; Figure 1B, black dashed line). To rule out that the unusual bending behavior upon binding to PA200 to the i20S is an artefact, we solved the cryo-EM structure of the s20S-PA200 complex. When comparing it to the deposited 6REY.pdb structure, we found an RMSD of ~0.2 Å for both the distal and proximal PA200 molecules (Figure 1B, orange dashed line and Figure S2). Moreover, when aligning the proximal α-rings of the s20S (7NAN.pdb) and i20S (6AVO.pdb), we observed an RMSD of ~1.6 Å between the two distal α-rings (Figure 1B, blue line and Figure S3) showing an only minor deviation of the two apo 20S complexes regarding α-rings. Thus, in the i20S-PA200 complex the entire axes of the complex bends when compared to s20S-PA200, resulting in a major shift of the opposite unbound α-ring (Figure 1C). Importantly, the same structural shifting/tilting behavior was evident in singly-capped i20S-PA200 structures (Figure 1B, black solid line, Figure 2A and S4A-C), implying that binding of the first PA200 molecule is sufficient to induce this long-range allosteric change. Of note, we observed similar increase in RMSD along the catalytic core when comparing our PA200-i20S-PA200 structure with the PA28αβ-i20S-PA28αβ (7DRW) (Figure 1B, dashed yellow line), confirming that this structural bending is specific to the i20S-PA200 complexes."**

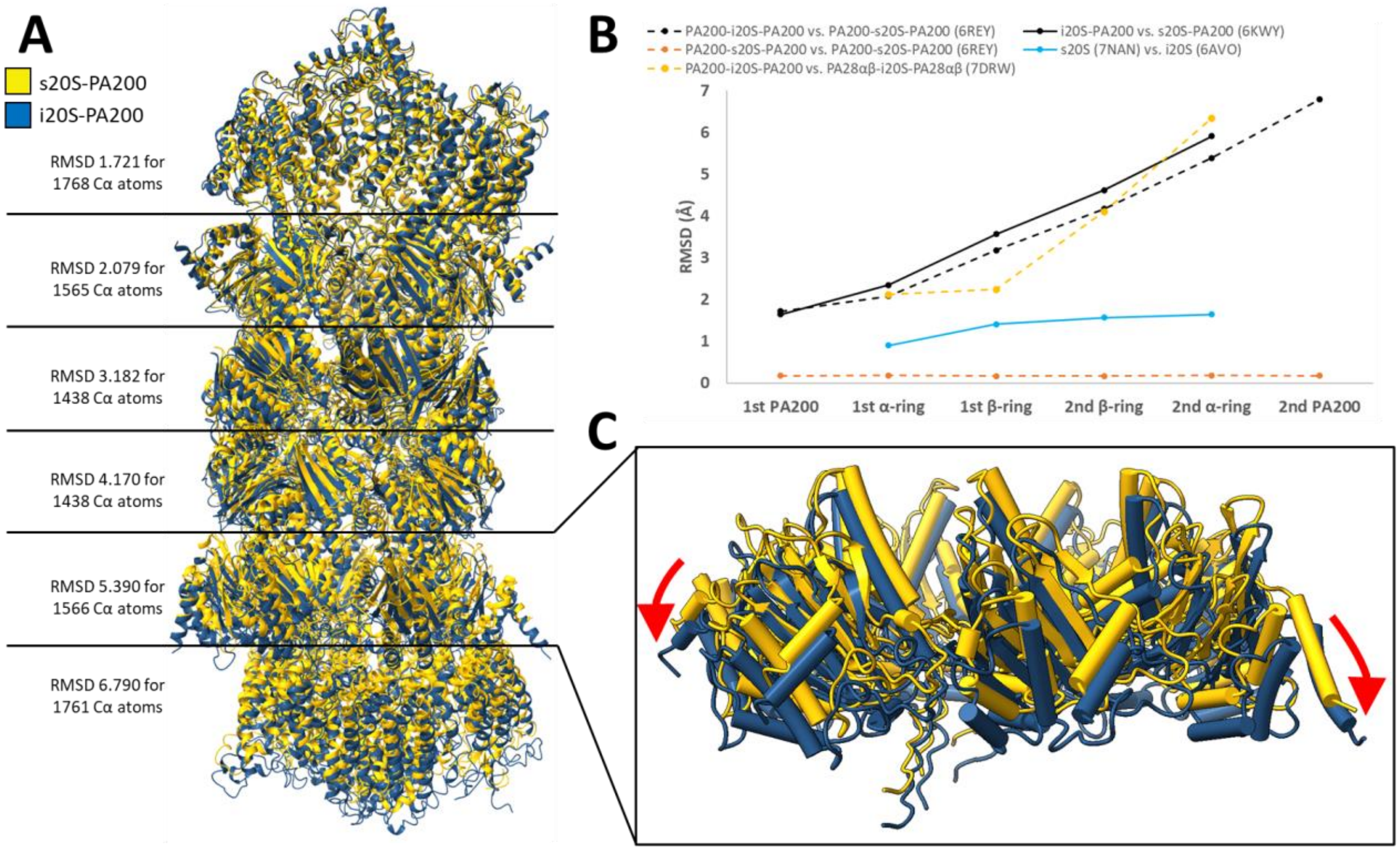

Revised Figure 1: Cryo-EM analysis of the i20S-PA200 proteasome complex. The structure of doubly-capped i20S shares overall similarity to the s20S-PA200 structures reported before. (**A**) Overlay of the i20S-PA200 (blue) and s20S-PA200 (yellow, PDB: 6REY). The structures were aligned by the PA200 molecule at the top. The overall axis of the complex is bent. Root Mean Square Deviations (RMSD, in Å) between both structures are indicated for the two PA200 molecules and for each one of the α and β ring. (**B**) Comparisons of the i20S-PA200 and s20S-PA200 structures (black lines) show a progressive increase of the RMSD from the top (first binding event) to the bottom of the barrel, reflecting the long-range allosteric bending of the barrel. Comparison of PA200-i20S-PA200 vs. PA28αβ-i20S-PA28αβ (7DRW.pdb) show similar RMDS increase (dashed yellow line), confirming that such bending is not occuring upon PA28αβ binding. As a control, we solved the structure of the PA200-s20S-PA200 complex (see Figure S2) and did not observe such bending when compared to the published one (6REY.pdb) (orange line). Similarly, comparison of the s20S (7NAN.pdb) vs. i20S (6AVO.pdb) structures (blue line) show only marginal deviations (see Figure S3). Solid and dashed lines correspond to singly- and doubly-capped complexes, respectively. (**C**) The α subunits furthest away from the aligned PA200 molecule show a substantial displacement (up to 5.4 Å).

While a reconstruction of human i20S bound to human PA28αβ would be highly interesting, it is, however, beyond the scope of the current study. We are happy to provide the in-house s20S–PA200 map/model to the editors or reviewers upon request. We have also extensively modified the main text to better describe our data.

Finally, we now provide a morph animation from the s20S-PA200 structure (yellow) to the i20S-PA200 structure (blue, see supplementary Video 1) showing, as stated in the text that: **“the bending of the i20S-PA200 results in a slight but significant decompaction of the whole complex”**

R2. The authors here claim that the genetic depletion of PA200 in A549 lead to a significant reduction of PSMB9. However, in Javitt et al. it is stated that, upon genetic depletion of PA200 in A549, ‘none of the genes with altered expression had known associations with tumour-immune interaction’ and ‘we found only a mild impact on gene expression’.

R2: We thank the reviewer for this important comment, how to reconcile our data with the data from the Javitt study. This indeed seems contradictory at first sight but there are important experimental differences between the Javitt and our study. In our experiments, we used A549 cells with a genetic deletion of PA200, while the Javitt study generated A549 cells with PA200 knock down using short hairpin RNA. As seen in the Javitt’s Extended Figure 6c (Figure 1A below for the Reviewer #1), this silencing experiment resulted in only partial depletion of PA200 with residual PA200 being present mainly in the cytoplasmic fraction. The main difference between the two studies is thus the complete knockout versus partial depletion of PA200 in the A549 cells. We would also like to highlight that we found PSMB9 reduced at the protein level in two different lung cancer cell lines using two different methods, i.e. Western blotting and proteomics analysis (Figure 7A-B and Suppl. Figure S5). At the RNA level, however, we did not observe significant downregulation of i20S genes in our PA200 KO cells (Figures for the Reviewer 1B-D). These additional data indicate that the effect we observed for PA200 KO cells is mediated by posttranscriptional regulation. In our revised manuscript, we have now omitted our data on the siRNA mediated knockdown of A549 cells (Suppl. Figure S6B) to consistently focus on sustained deletion of PA200. We have also modified our conclusion and also added a sentence in our revised discussion to clarify the differences between our data and the Javitt study: “**While the Javitt study did not observe any obvious regulation of the i20S subunits upon partial knockdown of PA200 in A549 cells, we would like to stress that we here used two different cell lines (A549 and H1299) and several genetically deleted PA200 clones to confirm reduced protein levels of PSMB9 by Western blotting and proteomics analysis. Hence, our data uncover a potential novel link between PA200 regulation and cellular composition of the proteasome that has not been reported before. Regulation of the i20S by PA200 may have major implications for antigenic repertoire generation. As such, reduced protein levels of β1i in PA200 deficient cells might contribute to an altered MHC-I antigenic diversity[Javitt et al. 2023]. Indeed, the replacement of a constitutive subunit by its immunosubunit counterpart, and vice versa, can result both in generation and destruction of specific antigenic epitopes[Guillaume et al. 2010; Basler et al. 2012]. Transcriptional regulation of i20S catalytic subunit expression and alteration of MHC I antigen presentation has been also demonstrated for PA28γ[Yao et al. 2019].**”

[editorial note: third party material redacted]

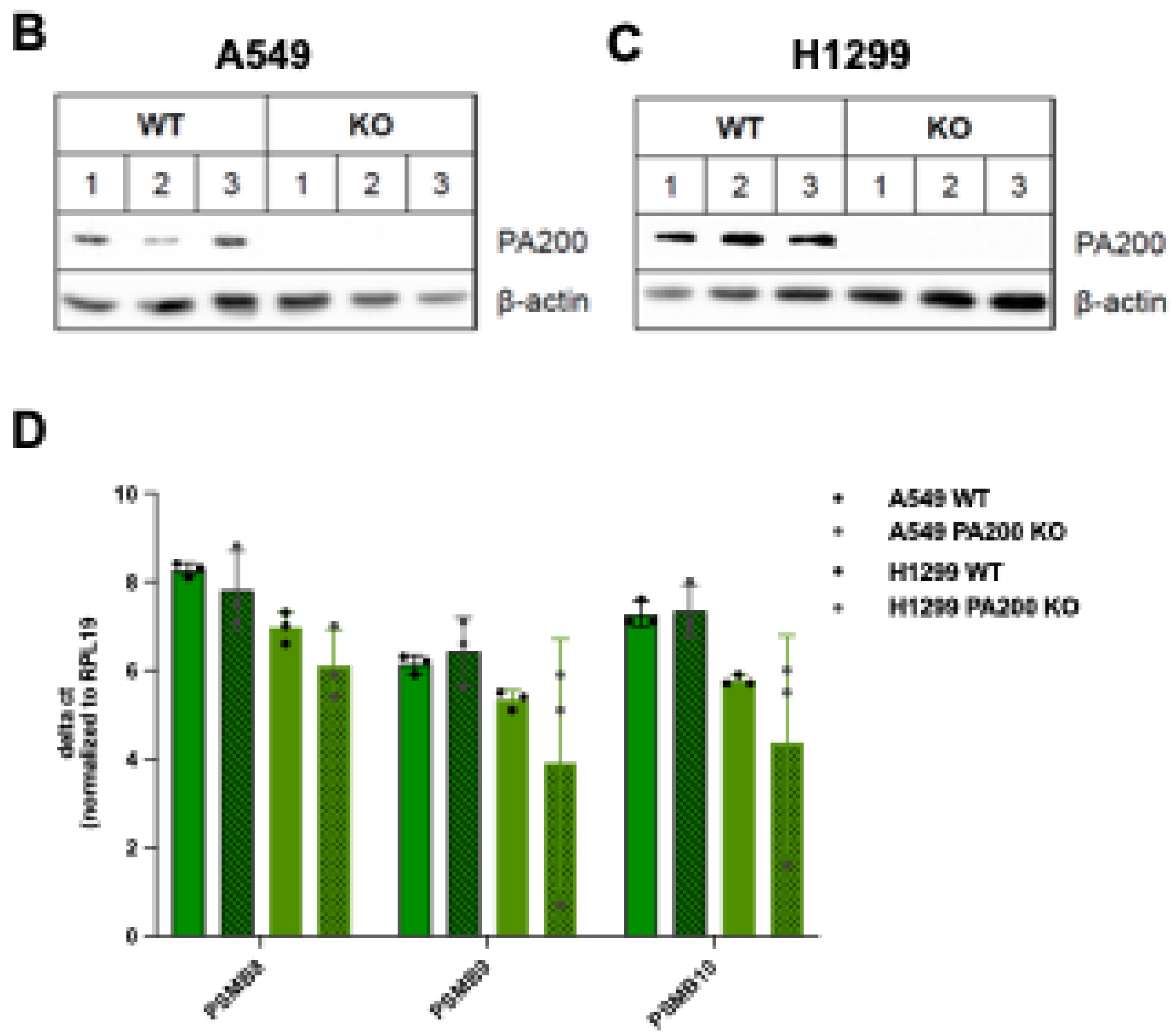

Figure 1 for the reviewer: (**A)** Extended Figure 6C from Javitt et al., 2023 study with caption: "Proteasome complexes were immunoprecipitated (IP) with PSMA1 from A549 cells (with PSME4 depletion or control) that were either treated with TNFα and IFNγ or left untreated and blotted for the indicated proteasome subunits. This experiment was repeated twice with similar results.". (**B**) Confirmation of PA200 deletion in A549 and (**C**) H1299 cells in three distinct WT and PA200 deleted clones, respectively, using Western blot analysis. (**D**) RTqPCR analysis of immunoproteasome genes PSMB8-10 in the same A549 and H1299 WT and PA200-deleted clones. Data are shown as delta ct values of the target genes relative to the housekeeper gene RPL19.

R3. The authors measured proteasome activity using standard short fluorogenic substrates, which could be used to test proteasome activity but might not represent the real degradation of substrates by proteasomes in vitro and in cellular systems. Based on what I see in Fig. 4, PA200 increases the degradation of all 3 substrates, with an effect more pronounced for i20S than s20S. However, in Javitt et al. it is stated that, 'abundance of PSME4 was positively correlated with the caspase- and

chymotryptic-like cleavage activities of the proteasome and negatively correlated with tryptic-like cleavage (Fig. 3c,d and Extended Data). Also, in Javitt et al., it is stated that 'We found that the caspase-like (β1) activity of the constitutive proteasome increased (Fig. 3g and Extended Data Fig. 7d), whereas the tryptic-like (β2) activity decreased, upon PSME4 supplementation (Fig. 3h and Extended Data Fig. 7e). By contrast, when examining the effect of PSME4 on immunoproteasome-associated activities, we found that all were inhibited (β1i, β2i and β5i). Honestly, I struggle to reconcile these results with what the authors propose in the submitted manuscript.

R3: We appreciate the reviewer's careful observation and fully agree with this important comment. Again, we would like to emphasize the experimental differences in the two studies. In their experiment depicted in Figure 4 and extended data sets 6 and 7, Javitt et al. stimulated the cells plus/minus a TNFα/IFNγ cocktail and obtained native protein lysates which were then supplemented with recombinant PA200. There is no PA200 supplemented control for non-stimulated A549 cells. They then measured activity in the extracts using different fluorogenic substrates. The main experimental differences can be summarized as follows:

1. Using fluorogenic peptide assay on cell lysates, as done in the Javitt et al., study has its limitations, due to the existence of peptidases that can create high background noise for some peptides used in the proteasome activity assay. Fluorigenic peptide assay will give a more precise activity under controlled conditions when used with purified enzymatic complexes and this is how we used it in the work we are currently submitting for your consideration.
2. Beyond this, a cellular lysate is not the native environment for proteasome complexes due to the lack of compartmentalization e.g. into nucleus and cytoplasm. As demonstrated in the Javitt et al. study and also by others, PA200 is found mainly in the nuclear fractions (Extended data Figure 6b, Javitt et al. provided as Figure 2A for reviewer #1). In a protein lysate this compartmentalization is, however, lost.
3. Finally, addition of PA200 to cell extracts will most likely induce a shift in proteasome complex composition. This is indeed seen in the size exclusion chromatography data set (Extended Figure 6h, Javitt et al. provided as Figure 2B for reviewer #1): addition of PA200 resulted in formation of hybrid proteasomes containing PA28αβ and PA200 and less 26S and 30S proteasome complexes. This would explain the observation of diminished proteasome activity upon addition of purified PA200 to the lysates in almost all assayed samples (except for one measurement). In our study, we performed in vitro experiments using isolated i20S and s20s and recombinantly expressed PA200 where we carefully controlled the assembly of singly and doubly capped complexes by mass-photometry (Figure 3A-C). Under these controlled conditions, we observed preferential activation of the i20S activities by PA200 (Figure 4).

[editorial note: third party material redacted]

Figure 2 for the reviewer: (**A)** Extended Figure 6B from Javitt et al., 2023 with caption: "Cytosolic and nuclear cellular fractions of A549 were stained with Coomassie (a) and immunoblotted for PSME4 (b). GAPDH and Histone H3 were used as cytosolic and nuclear markers respectively.". (**B**) Extended Figure 6H from Javitt et al., 2023 with caption: "Lysates were separated by size using Superose6 gel filtration column and blotted against different proteasome subunits as indicated."

We do not claim that the observations in the Javitt et al. article are incorrect, but only that there are experimental discrepancies which we mention in the points above. We thus feel that we cannot make direct comparisons between the two articles. To clarify the discrepancies, we added a paragraph to the revised manuscript in the discussion section: **"The discrepancies between our data on the activation of PA200 versus the observed inhibition of inflammation-induced activation of the i20S as observed by Javitt et al. can be explained by the different experimental conditions. In our study, we performed *in vitro* experiments using isolated i20S and s20S and recombinantly expressed PA200 where we carefully controlled the assembly of singly and doubly capped complexes by mass-photometry. Under these controlled conditions, we observed preferential activation of the i20S activities by PA200. In contrast, Javitt et al.**

stimulated A549 lung cancer cells plus/minus an inflammatory TNFα/IFNγ cocktail and obtained native protein lysates which were then supplemented with recombinant PA200. Different proteasomal activities were determined in the protein lysate using fluorogenic substrates. The addition of recombinant PA200 to cell lysates might cause a shift in the overall composition of proteasome complexes as seen for example observed as with increased binding of PSME1 binding (Extended data Figure 6h[Javitt et al. 2023]), thereby adding to a complex and not well controlled effect of PA200 on proteasome activity in the cell extracts."

4. A way to clarify these discrepancies could be performing digestions assays of entire proteins using i20S and different proteasome activators, and measure the peptide product via MS and the protein degradation rate via SDS-gel.

R4: The authors thank the reviewer for suggesting this experiment. We performed *in vitro* digestion assays using acetylated histones, which — to date — remain the only substrates known to be selectively degraded by the PA200-activated proteasome. We used a PA200:20S molar ratio of 8:1 and followed the experimental conditions described in Figure 6D of the study by Qian et al. (DOI: 10.1016/j.cell.2013.04.032).

Our results confirm that PA200-activated 20S proteasomes degrade acetylated histones more efficiently than non-acetylated histones (panel A, Figure 3 for the reviewer), and that free 20S has a poorer activity (panel B, Figure 3 for the reviewer). When analyzing three replicates of acetylated histones degradation assays (panel C, Figure 3 for the reviewer), we also observe a slight but non-significant 1.6-fold increased degradation rate with PA200-activated i20S compared with PA200-activated s20S (panel D, Figure 3 for the reviewer). These findings demonstrate for the first time that both 20S subtypes (standard and immunoproteasome) can degrade acetylated histones when bound to PA200, but that the immunoproteasome is intrinsically not more efficient.

Although acetylated histones from calf thymus are unlikely to represent the optimal physiological substrate of human PA200-activated i20S, they remain the only established substrates commercially available. Identifying the physiological targets of the PA200–i20S proteasome will therefore be an important next step that is beyond the scope of our present manuscript. Our study lays the groundwork for future investigations exploring the biological and pathological contexts in which PA200-mediated activation of the immunoproteasome may play a functional role.

This last sentence has been added in the conclusion of the revised manuscript.

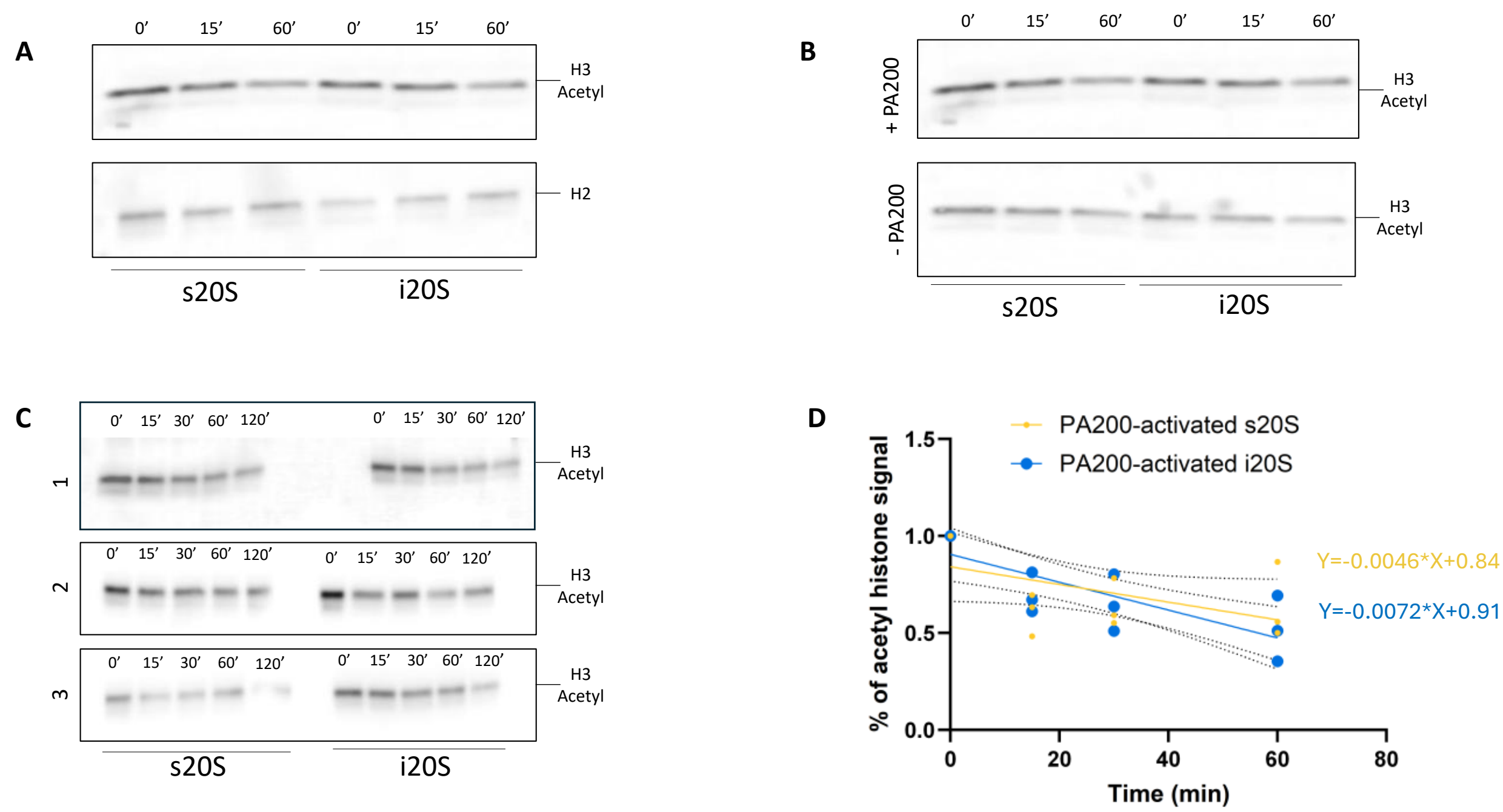

Figure 3 for the Reviewer: A, B, C: The two proteasome subtypes, s20S and i20S, were incubated with recombinantly expressed PA200 (molar ratio PA200:20S = 8:1) and a mix of bovine histones. The activity was assayed by collecting the solution at different time points and stopping the reaction by SDS denaturation. The degradation of acetylated H3 histones was then analyzed by immunoblotting (anti-Pan acetyl H3 and anti H2). **D**: Band intensities corresponding to H3 acetylated on each Western blot were quantified by densitometry and normalized by 0 min time point intensity. Those intensities were plotted and a simple linear regression fit model was applied. The two slopes show a ratio of 1.6 in favor of PA200-activated i20S but are not significantly different at a 95% confidence level (p=0.38).

Minor comments:

5. I think there is a problem with the reference enumeration between lines 80 and 88. It jumps from 10 to 15 and then back to 13 and later 11,12.

R5: The authors thank the reviewer for pointing out this mistake. The reference numbers are now correctly listed.

6. I do not see the advantage of citing a review 21 years old (ref 1). Many updated reviews have been published since then.

R6: We decided to keep the 21 years old review to acknowledge the pioneer work of this group, and as advised by the reviewer, we have also added a more recent review that emphasizes the interest of targeting the proteasomes and MHC I machineries in various diseases (DOI: 10.3390/cancers15235632).

Reviewer #3 (Remarks to the Author)

Summary:

This study explores the structural and functional interplay between PA200 and the immunoproteasome (i20S). It reveals mechanisms that distinguish its regulation from that of the standard proteasome (s20S). Using cryo-EM, the authors show that binding of a single PA200 particle induces long-range conformational rearrangements in the i20S core, thereby facilitating subsequent engagement of a second PA200 and enhancing catalytic activity. Mass photometry supports increased occupancy and affinity of PA200 for i20S over s20S, while activity assays demonstrate more robust activation of i20S, especially in the presence of inositol hexakisphosphate (IP6). Proteomic and pull-down data reinforce the preferential binding of PA200 to i20S in tissues, and gene expression studies suggest a possible regulatory role for PA200 in shaping i20S composition.

Overall, this is a solid manuscript, but not a terribly exciting one. Some of the results are interesting, but I am not convinced the manuscript as a whole measure up to the level required for publication in Nat Comm. Perhaps a more specialized journal would be more appropriate.

Major Comments:

7. The structural data are the most interesting aspect of the manuscript. However, the analysis feels underdeveloped. The claim of a "bent" conformation in the singly- and doubly-capped i20S-PA200 complexes would benefit from more rigorous analysis. Currently, the structural figures do not convincingly convey this claim. Is this a true bend or a translational offset? The authors should attempt alternative alignment strategies and clearer visualization (e.g., angular displacement measurements, movies, or vector displacement plots) would help clarify this. The proposed allosteric effects, particularly on the opposite α-ring, are central to the manuscript and deserve a more thorough, quantitative description.

R7: We are grateful for the reviewer to raise this important point. According to his/her suggestion, we have now modified Figure 1 and included RMSD measurements as a graph depicting the comparative displacement for the different structures (revised Figure 1B). This way, the structural bent becomes clearly evident when comparing i20S/PA200 to s20S/PA200 structures. In addition, we now provide a morph animation from the s20S-PA200 structure (yellow) to the i20S-PA200 structure (blue, see supplementary Video 1) showing, as stated in the text that: **"the bending of the i20S-PA200 results in a slight but significant decompaction of the whole complex".** We have also added a comparison to the available structure of i20S-PA28αβ as outlined in response R1 and revised Figure 1B to prove that this bending is specific for the binding of PA200 to the i20S and does not occur with another proteasome activator. The text for the results part has been changed

accordingly to: **"Here, we obtained high resolution structures for both singly- and doubly-capped i20S-PA200 complexes (2.85 Å and 2.89 Å resolution, respectively). Although the doubly-capped structure of the i20S-PA200 complex seems similar to s20S-PA20020-23 (Figure 1A, see Figure S1 for details on 3D classification), the structure shows a remarkable "bend" of the entire complex (Figure 1B-C) which has not been observed in proteasome structures before. The bend propagates from the proximal PA200 molecule, which was superimposed with the corresponding PA200 in recently solved s20S-PA200 structure (6REY.pdb) with minimal Root Mean Square Deviation (RMSD~1.7 Å; Figure 1B, black dashed line). From that region, the structure of i20S-PA200 begins to "bend", as indicated by the gradual increase in calculated RMSDs to its maximal value in the distal PA200 (RMSD ~6.8 Å; Figure 1B, black dashed line). To rule out that the unusual bending behavior upon binding to PA200 to the i20S is an artefact, we solved the cryo-EM structure of the s20S-PA200 complex. When comparing it to the deposited 6REY.pdb structure, we found an RMSD of ~0.2 Å for both the distal and proximal PA200 molecules (Figure 1B, orange dashed line and Figure S2). Moreover, when aligning the proximal α-rings of the s20S (7NAN.pdb) and i20S (6AVO.pdb), we observed an RMSD of ~1.6 Å between the two distal α-rings (Figure 1B, blue line and Figure S3) showing an only minor deviation of the two apo 20S complexes regarding α-rings. Thus, in the i20S-PA200 complex the entire axes of the complex bends when compared to s20S-PA200, resulting in a major shift of the opposite unbound α-ring (Figure 1C). Importantly, the same structural shifting/tilting behavior was evident in singly-capped i20S-PA200 structures (Figure 1B, black solid line, Figure 2A and S4A-C), implying that binding of the first PA200 molecule is sufficient to induce this long-range allosteric change. Of note, we observed similar increase in RMSD along the catalytic core when comparing our PA200-i20S-PA200 structure with the PA28αβ-i20S-PA28αβ (7DRW) (Figure 1B, dashed yellow line), confirming that this structural bending is specific to the i20S-PA200 complexes."**

8. The mass photometry experiments suggest differential binding affinity of PA200 to i20S and s20S, but the quantitative conclusions are overstated. The fits are poorly constrained and clearly underestimate uncertainty. Mass photometry is best viewed as qualitative or semi-quantitative; definitive conclusions about dissociation constants require orthogonal validation, e.g., surface plasmon resonance or isothermal titration calorimetry. If the authors wish to report Kd values, confidence intervals (obtained properly) and goodness-of-fit metrics should be included in the main figures, not only the supplement.

R8: We thank the reviewer for pointing this out and agree that the description of the mass photometry measurement and Kd calculations was slightly overlooked in the first submitted version. As also requested by reviewer #2 (comment #27), we have now added the fraction of total

binding as a function of free PA200, full details about the fitting, including goodness-of-fit and confidence intervals in our revised Figure 3. The Materials & Methods part has been changed accordingly. Concerning the use of more established techniques to estimate Kds, such as SPR or ITC, we opted in this work for mass photometry, because, despite its inherent higher variability, it has the huge advantage of working at nM concentrations. Indeed, the amount of 20S proteasome that can be immunopurified from HEK cells is hardly compatible with ITC or SPR methods.

Mass photometry presents other key advantages over these established techniques, including the possibility to detect and quantify single molecule binding events (including intermediate microstate species), also allowing Kd calculations while working in the nanomolar range. Measurements are performed in solution with minimal protein consumption, without fluorescent labeling or immobilization, which helps preserve the native structure and dynamics of the complex (DOI: 10.1126/science.aar5839). Finally, studies have shown strong agreement between Kd measured by mass photometry and those obtained by established methods such as ITC or SPR, validating its reliability (DOIs: 10.1038/s41467-020-15553-2; 10.1002/anie.202000462).

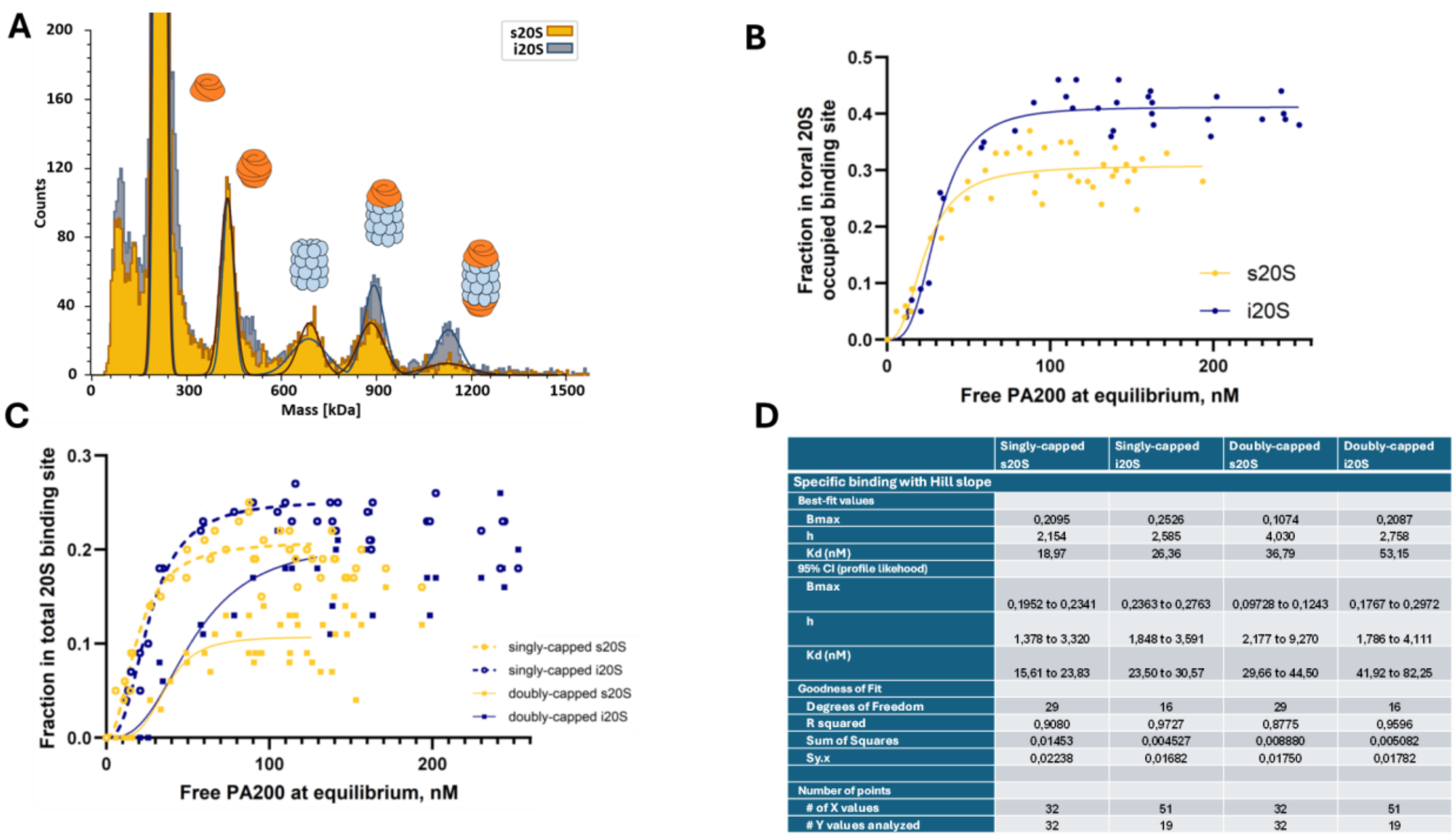

	Singly-capped s20S	Singly-capped i20S	Doubly-capped s20S	Doubly-capped i20S
Specific binding with Hill slope				
Best-fit values				
Bmax	0,2095	0,2526	0,1074	0,2087
h	2,154	2,585	4,030	2,758
Kd (nM)	18,97	26,36	36,79	53,15
95% CI (profile likehood)				
Bmax	0,1952 to 0,2341	0,2363 to 0,2763	0,09728 to 0,1243	0,1767 to 0,2972
h	1,378 to 3,320	1,848 to 3,591	2,177 to 9,270	1,786 to 4,111
Kd (nM)	15,61 to 23,83	23,50 to 30,57	29,66 to 44,50	41,92 to 82,25
Goodness of Fit				
Degrees of Freedom	29	16	29	16
R squared	0,9080	0,9727	0,8775	0,9596
Sum of Squares	0,01453	0,004527	0,008880	0,005082
Sy.x	0,02238	0,01682	0,01750	0,01782
Number of points				
# of X values	32	51	32	51
# Y values analyzed	32	19	32	19

Revised Figure 3: i20S-PA200 particles are preferentially formed compared to s20S-PA200 complexes. (A) Mass photometry allowed us to semi-quantify the relative abundances of free 20S (720 kDa), singly-capped 20S (930 kDa), and doubly-capped 20S (1,140 kDa). The PA200:20S molar ratio we used was 8. **(B)** Titrations of PA200 to either s20S and i20S showed a higher total occupancy of the i20S. The total 20S occupied binding sites were calculated by summing the fractions of singly- and doubly-capped 20S and as detailed in the Materials and Methods section. (C) i20S-PA200 doubly-capped particles are more abundant compared to s20S-PA200 complexes. Titrations of PA200 to either s20S and i20S showed that the higher occupancy of the i20S is mainly due to a higher population of doubly-capped complexes. **(D)** The experimental data points were fitted with the Hill curve at 95% confidence interval. The curve fit takes into account the whole dataset obtained for free PA200 concentrations up 130 nM (up to the plateau). The goodness-of-fit metrics is also included.

9. Kinetic characterization of PA200 binding would benefit from a more complete kinetic characterization of PA200 binding. Time-course measurements or other kinetic approaches would allow for more precise comparisons between the i20S and s20S binding behavior, rather than relying on endpoint titrations and bar plots.

R9: Classical biophysical approaches to perform such kinetic characterization of PA200 (ITC, SPR) are precluded in this case due to the amount of 20S proteasome that we can immune-purify from human cell lines. This is why we initially opted for mass photometry, even though it is a more recent and less established methodology. Of note, mass photometry has been successfully used for measuring Kd values and comparing them with ITC (DOI: 10.1002/anie.202000462).

10. The activity assays provide useful functional insight, but the presentation is limited to relative values and bar plots. Raw fluorescence data or slope-derived rates should be shown in supplementary figures to allow assessment of assay quality.

R10: We thank reviewer #3 for pointing this out and agree that activity assays deserve a more careful description. We now provide more detailed information in the revised manuscript (Materials and Methods part) and supplementary information (Table S2).
First, the raw fluorescent data were used to calculate the slope-derived rates as more precisely described in the revised version of the Materials and Methods: **"The kinetic assays were performed at 37 °C in a CLARIOstar Plus spectrofluorometer (BMG Labtech) over 60 min with one reading every 5 min, at 360 nm for excitation and 460 nm for emission. The gain of the fluorimeter was adjusted for each series of experiments to obtain the highest signal while avoiding saturation. The slope of the kinetic assay (increase in fluorescence intensity over time) was used to measure proteasome activity, after 15-minute temperature equilibration time. For each series of experiments, slope calculations were performed using a minimum of 5 timepoints, and always the same kinetic times; a threshold coefficient of determination (r2) corresponding to data linearization was set at 0.98. All the slope-derived rates corresponding to Figure 4 are shown in supplementary Table 1"**
You can find below an example of raw data and slope-derived rates calculations corresponding to Figure 4A/Chymotrypsin-like experiment, for the PA200:20S molar ratio of 0:1:

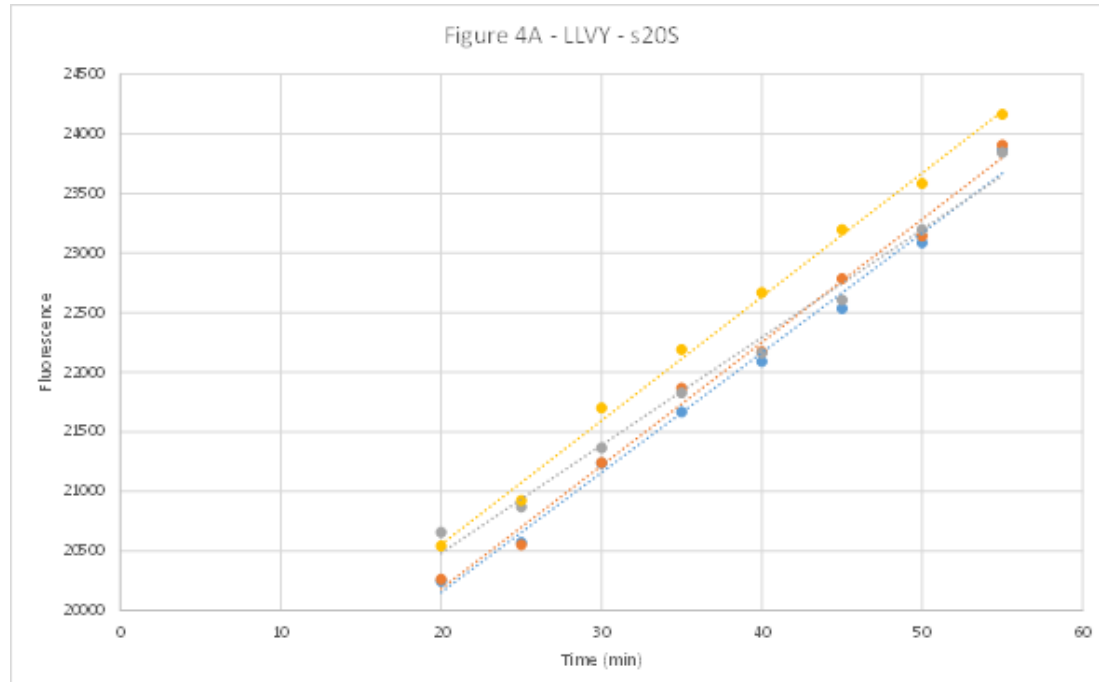

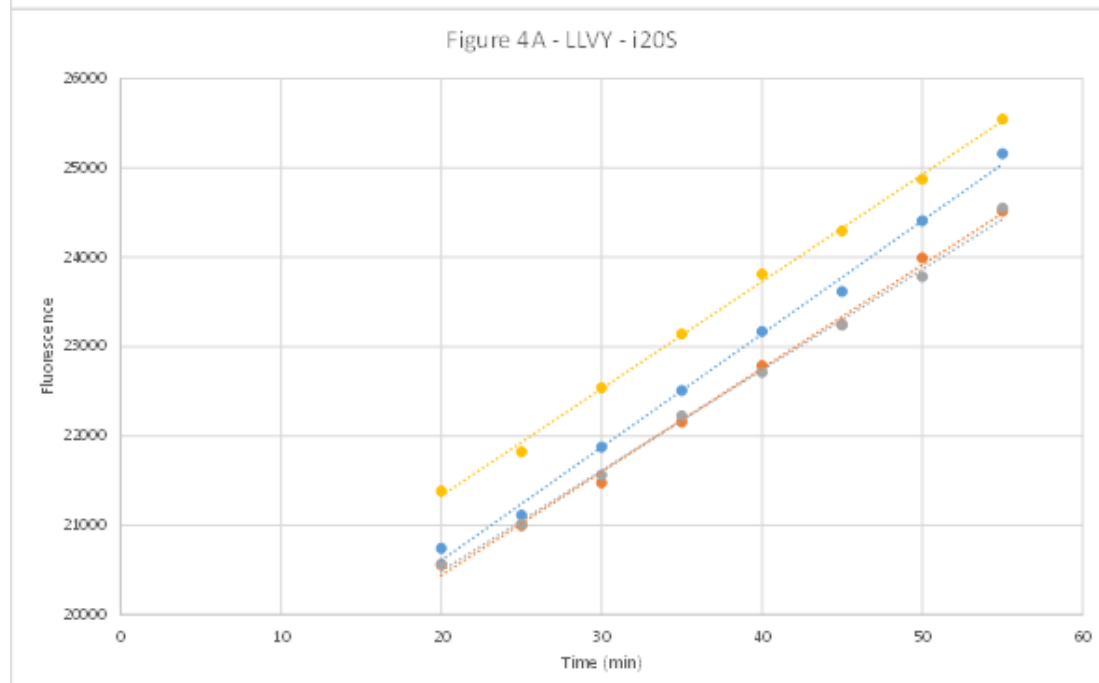

Well	Content	Raw Data (350-15/440-20)	Raw Data (350-15/440-20)	Raw Data (350-15/440-20)	Raw Data (350-15/440-20)	Raw Data (350-15/440-20)	Raw Data (350-15/440-20)	Raw Data (350-15/440-20)	Raw Data (350-15/440-20)	Raw Data (350-15/440-20)	Raw Data (350-15/440-20)	Raw Data (350-15/440-20)	Raw Data (350-15/440-20)	sample	R2	slope	normalized slope (s20S)
	Time, min	0	5	10	15	20	25	30	35	40	45	50	55				
A21	Sample X57	19173	18938	18905	19474	20247	20572	21237	21667	22090	22536	23088	23861	s20S +	0,996	100,47	1,01
A22	Sample X57	19537	19269	19157	19551	20264	20552	21241	21867	22172	22786	23145	23908	PA200 (1:0)	0,996	103,36	1,04
A23	Sample X57	19715	19457	19556	19836	20657	20871	21366	21828	22163	22607	23196	23845		0,994	90,47	0,91
A24	Sample X57	19627	19354	19536	20073	20543	20925	21700	22191	22670	23197	23585	24166		0,998	103,88	1,04
G21	Sample X63	19284	19179	19470	20082	20744	21116	21876	22509	23170	23617	24409	25162	i20S +	0,998	126,85	1,27
G22	Sample X63	19539	19400	19676	19959	20557	20996	21477	22159	22788	23249	23994	24518	PA200 (1:0)	0,998	115,86	1,16
G23	Sample X63	19467	19396	19578	19987	20566	21014	21562	22225	22713	23243	23783	24552		0,999	112,57	1,13
G24	Sample X63	19694	19571	20068	20428	21385	21823	22541	23144	23813	24296	24876	25549		0,999	119,87	1,20

All the slopes obtained in Figure 4 (A, B, C) are now provided in the new supplementary Table S1. The mean R^2 value (coefficients of determination derived from all the linear fitting of the data) is equal to 0.990 +/- 0.017 (n=247). A minimal R^2 threshold of 0.98 has been used (as explained in the revised paragraph above in the Materials and Methods).

It would also be helpful to present basal activity levels for i20S and s20S side-by-side, especially in Figure 4B and 4C. This would better contextualize the extent of activation by PA200 and IP6.

Figures 4B and 4C show two different experimental series (corresponding to i20S or s20S activation by PA200 and IP6) that were not obtained with the same gains (fluorimeter parameters adapted to the signal, as explained in the Materials and Methods), and the data cannot be directly compared.
However, the side-by-side basal activity levels of i20S and s20S, activated or not with different molar ratios of PA200, can already be obtained from Figure 4A (yellow vs. blue bars) since these values were obtained with the same fluorimeter parameters (same gain). In this panel A, all the

slope values were normalized with the one of the s20S (mean of 5 replicates). You can find below the raw values of the corresponding slopes (now published in the new supplementary Table 2).

Sample	molar ratio	LLVY	Chymotryptic-like _ slope-derived rates				LRR	Tryptic-like _ slope-derived rates				LLE	Caspase-like _ slope-derived rates			
		1	2	3	4	5	1	2	3	4	5	1	2	3	4	5
s20S+PA200	1:0	100,47	103,36	90,47	103,88	101,54	56,52	63,27	49,41	59,97	58,84	772,99	746,82	781,76	694,33	728,54
	1:2	277,59	269,42	248,99	248,72	208,06	117,24	107,82	109,94	112,88	112,11	1345,46	1371,14	1350,73	1306,38	1277,97
	1:4	373,11	356,96	356,28	314,99	250,86	100,31	111,25	98,36	104,36	105,79	1323,41	1248,55	1246,45	1252,49	1166,61
	1:8	392,22	400,57	416,32	387,91	481,81	140,8	149,7	126,1	143,01	142,76	2187,55	2054,87	2008,92	1991,68	1950,92
i20S+PA200	1:0	126,85	115,86	112,57	119,87	70,08	47,32	56,03	58,57	57,84	62,51	12,08	18,38	9,82	8,13	17,72
	1:2	438,35	398,14	416,40	440,20	336,17	130,6	145,13	143,55	149,53	151,95	43,49	45,83	51,07	38,67	44,95
	1:4	521,18	493,65	515,84	531,49	502,47	153,87	164,33	171,72	168,87	171,08	76,32	76,23	82,03	70,83	73,23
	1:8	636,75	629,83	611,75	578,57	742,42	197,37		211	213,1	187,7	116,12	115,73	125,4	114,28	110,15

11. The assignment of IP6 in the density maps is presented with little analytical support. Is this modeling based solely on fitting to positive density? If so, this should be made explicit, and the limitations acknowledged.

R11: The assignment of IP4 and IP6 was reported previously in Rego et al. paper (DOI: 10.1016/j.molcel.2019.07.014 Fig 6 and S5), and is also confirmed by both CryoEM and MS in the Guan et al. paper (DOI: 10.1371/journal.pbio.3000654) where the authors show electron microscopy with excellent resolution on Fig 5D (at S7 D-E) and detailed mass spectrometry on Figure 6. In addition, the chemical environment of PA200 channels determines its affinity to -COOH groups. In the light of our and other's published data, we consider the assignment of IP6 in our structures as fully validated, which was further confirmed by our *in-vitro* assays with fluorogenic peptides. We have clarified this and cited the respective papers in the results section **"Of note, we identified extra density in the two positively charged grooves of PA200 that were previously identified as inositol hexakisphosphates (IP6, phytic acid) and (5,6)-bisdiphosphoinositol tetrakisphosphate (5,6[PP]2-IP4) in the s20S-PA200 complexes[20-23]. Based on these previous results, we assigned these densities to two molecules of IP6."** and in the conclusion **"The two teams who solved the s20S-PA200 structures and identified IP6 and 5,6[PP]2-IP4 in the positively charged pockets hypothesized that they could be involved in regulating the activity of PA200, and more precisely in the degradation of acetylated histones[20,21]."**

Additionally, the rationale for using s20S basal activity as the reference in all comparisons is unclear. For clarity, activation effects should be expressed relative to the appropriate unbound control for each proteasome type.

As the reviewer rightly pointed out, in the case of Figures 4B and 4C, normalization should be based on the catalytic activity of the appropriate non-activated 20S proteasome (i20S for panel B and s20S for panel C). This is actually what we did, but we agree that it was not clearly explained in the manuscript. This is now more clearly explained in the revised legend of Figure 4, as follows: **"The activity measured with the non-activated i20S (panel B) or s20S (panel C) was used as**

the reference activity.”. This important point was also added in the Materials and Methods: **“Activation effects were expressed relative to the appropriate unbound control for each proteasome type: s20S in Figure 4A and 4C, and i20S in Figure 4B”.**

12. The evidence suggesting that PA200 regulates i20S subunit expression is intriguing but not fully convincing. The error bars are large, and the mechanistic basis is unexplored. Additional transcriptomic or pathway analyses would help strengthen this section. At minimum, the authors should acknowledge the limitations of these findings and avoid overinterpreting their significance.

R12: We thank the reviewer for their comment and agree that the evidence is not backed up by any mechanistic insight. As already outlined in our response to reviewer #1, we would like to emphasize that we observed downregulation of PSMB9 on the protein level using two different methods, i.e. Western blotting and proteomics analysis. We also analyzed RNA expression to investigate transcriptional regulation in the two PA200-deleted cancer cell lines, but observed no differences compared to the respective WT controls (Figure 1D for reviewer #1, see R2). This observation was confirmed in transcriptome data sets which we performed to understand the cellular effects of PA200 deletion in lung cancer cell lines. These data are part of another study that is currently in preparation for submission. The transcriptome data did not provide any evidence for a conserved transcriptional regulation of the i20S by PA200, they rather revealed a striking cell-specific effect of the loss of PA200 depending on the cell line.
According to the reviewer’s suggestion, we have thus modified and toned down our statement on the regulation of PSMB9 by PA200. We have also omitted our supplementary data on the siRNA-mediated silencing of PA200 in A549 cells as this partial depletion of PA200 represents a different experimental outline (acute and only partial PA200 depletion) and might rather confuse the reader. In the discussion, we now state: **“Hence, our data uncover a potential link between PA200 regulation and cellular composition of the proteasome. Regulation of the i20S by PA200 may have implications for antigenic repertoire generation.”**

Minor Comments:
13. Line 112: The term "bend" to describe the i20S-PA200 conformation is not clearly supported by the figures. Consider rewording or providing clearer visual evidence (e.g., morph animations or angle measurements). The figure needs to be improved and polished.

R13: As requested by the reviewer, we now provide a morph animation from the s20S-PA200 structure (yellow) to the i20S-PA200 structure (blue, see supplementary Video 1) showing, as stated in the text that: **“the bending of the i20S-PA200 results in a slight but significant decompaction of the whole complex”**. We also modified Figure 1 to add a comparative RMSD analysis between our structures and other published ones (Revised Figure 1B), which shows that

the bending of the structure is a unique feature of the i20S-PA200 complex. We modified the text of the revised results part accordingly: **"Here, we obtained high resolution structures for both singly- and doubly-capped i20S-PA200 complexes (2.85 Å and 2.89 Å resolution, respectively). Although the doubly-capped structure of the i20S-PA200 complex seems similar to s20S-PA20020-23 (Figure 1A, see Figure S1 for details on 3D classification), the structure shows a remarkable "bend" of the entire complex (Figure 1B-C) which has not been observed in proteasome structures before. The bend propagates from the proximal PA200 molecule, which was superimposed with the corresponding PA200 in recently solved s20S-PA200 structure (6REY.pdb) with minimal Root Mean Square Deviation (RMSD~1.7 Å; Figure 1B, black dashed line). From that region, the structure of i20S-PA200 begins to "bend", as indicated by the gradual increase in calculated RMSDs to its maximal value in the distal PA200 (RMSD ~6.8 Å; Figure 1B, black dashed line). To rule out that the unusual bending behavior upon binding to PA200 to the i20S is an artefact, we solved the cryo-EM structure of the s20S-PA200 complex. When comparing it to the deposited 6REY.pdb structure, we found an RMSD of ~0.2 Å for both the distal and proximal PA200 molecules (Figure 1B, orange dashed line and Figure S2). Moreover, when aligning the proximal α-rings of the s20S (7NAN.pdb) and i20S (6AVO.pdb), we observed an RMSD of ~1.6 Å between the two distal α-rings (Figure 1B, blue line and Figure S3) showing an only minor deviation of the two apo 20S complexes regarding α-rings. Thus, in the i20S-PA200 complex the entire axes of the complex bends when compared to s20S-PA200, resulting in a major shift of the opposite unbound α-ring (Figure 1C). Importantly, the same structural shifting/tilting behavior was evident in singly-capped i20S-PA200 structures (Figure 1B, black solid line, Figure 2A and S4A-C), implying that binding of the first PA200 molecule is sufficient to induce this long-range allosteric change. Of note, we observed similar increase in RMSD along the catalytic core when comparing our PA200-i20S-PA200 structure with the PA28αβ-i20S-PA28αβ (7DRW) (Figure 1B, dashed yellow line), confirming that this structural bending is specific to the i20S-PA200 complexes."**

14. Figure 1: Consider including a comparison of apo i20S and s20S structures to highlight differences prior to PA200 binding. RMSD comparisons could be informative.

R14: As suggested by the reviewer, we have now added a comparison of the apo s20S and i20S structures, using PDB files 7NAN and 6AVO, respectively. As expected, the differences observed between both structures are only marginal, compared to what is observed on the i20S-PA200 complex. These results were included in Figure S3 and Figure 1B (blue line) and the manuscript was modified accordingly (see text above).

15. Figure 2A: While peripheral helices show changes, alterations in the axial channel are less

apparent. Quantitative pore measurements or cross-sectional diagrams would help clarify these structural changes.

R15: We thank the reviewer for this important suggestion. Accordingly, we have measured the α-ring's diameters (proximal & distal) and the aperture of the pore (proximal & distal) for all the structures analyzed (i.e. s20S, i20S, s20S-PA200, PA200-s20S-PA200, i20S-PA200, PA200-i20S-PA200, i20S-PA28αβ and PA28αβ-i20S-PA28αβ) and have now included this data as new panels of revised Figure 2 (Figure 2C and D), respectively.

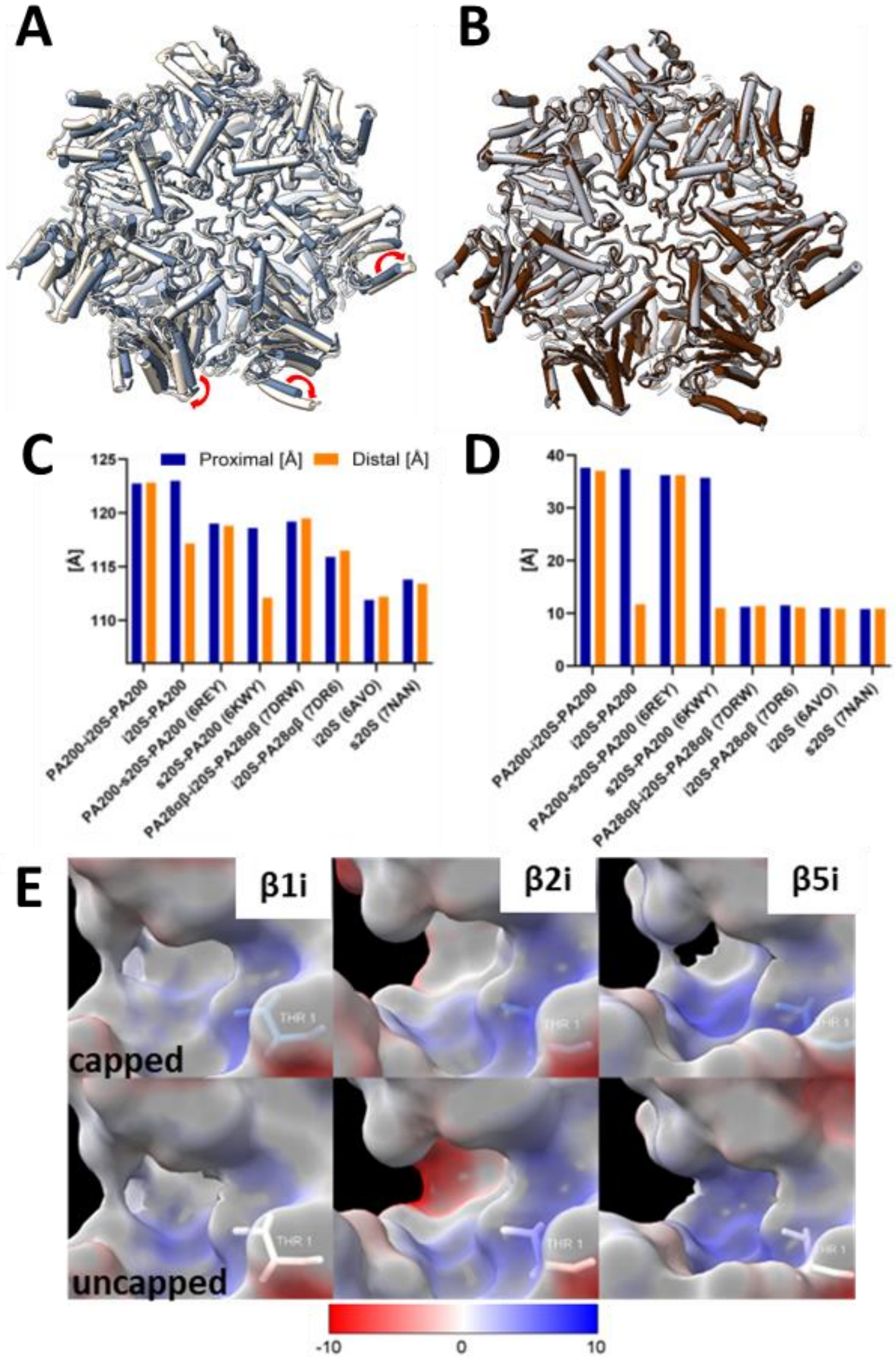

Revised Figure 2. Binding of PA200 induces channel opening of i20S, allosterically alters the catalytic site and widens the opposite unbound α-ring. Superimposition of the (**A**) unbound side of the singly-capped i20S-PA200 (white) with uncapped i20S (blue, PDB ID: 6AVO) and (**B**) unbound side of the singly-capped s20S-PA200 (grey, PDB ID: 6KWY) with the uncapped s20S (brown, PDB ID: 6RGQ), (**C**) Measure of the proximal (blue) and distal

(orange) α-ring diameters in different complexes show that PA200 binding to the i20S induces the largest α-ring widening. (**D**) Measure of the proximal and distal α-ring gate diameters shows that its apparent size is larger upon binding of PA200 compared to the apo and PA28αβ bound form. (**E**) Comparison of active sites of the capped (top) and uncapped (bottom) i20S colored by electrostatics (positive in blue and negative in red).

Altogether, and as stated in the revised version of the manuscript, these results show that:

1) **"PA200 binding to the i20S induces the largest α-ring widening (Figure 2A-C). Interestingly, this widening was allosterically initiated by the first binding event and further completed by the second PA200 binding event.**

2) **"In contrast, the same comparison of the apo s20S and s20S bound to a single PA200 regulator shows almost perfect overlay and no widening of the distal α-ring (Figure 2~~B-~~C)"**

We believe that this analysis clearly shows the widening of the second α-ring upon binding of the first PA200 regulator to the i20S but not s20S.

3) "**The i20S-PA200 complex shows a degree of channel opening comparable to that of the s20S-PA200 (35-37 Å; Figure 2D), which, as expected, is significantly higher than that of the apo s20S and i20S complexes, but remarkably larger than that of the only partially open s20S-PA28αβ[(Zhao et al. 2022)] and i20S-PA28αβ[(Chen et al. 2021)] complexes (9-11 Å) (Figure 2D). This suggests that PA200 opens the gate of the s20S and i20S particles to a wider extent than PA28αβ. However, since the N-termini of the α-ring are missing in the i20S-PA200 and s20S-PA200 structures, this apparent wider opening of the gate is probably rather reflecting a more dynamic state of the gate, that precludes its proper density assignment. Importantly, no differences were observed in that regard when comparing the i20S-PA200 vs. s20S-PA200 structures."**

16. Line 140: The discussion of active-site rearrangements is vague. A structural figure showing local RMSD of active site residues would help.

R16: We thank the reviewer for their suggestion and have accordingly measured the local RMSD of active site residues (see Table 1 for the reviewer). The changes we observed were similar (although slightly more pronounced) than the ones found upon binding of PA200 to the s20S. Since this is not the major point of the article, we decided to mention that binding of PA200 to the i20S was inducing allosteric changes similarly to the binding of PA200 to s20S (DOI: 10.1016/j.molcel.2019.07.014, 7NAN). We have accordingly modified our statement on active site remodeling of PA200/i20S complexes: "**The subtle structural rearrangements within active**

sites of all three catalytic subunits (β1i, β2i, β5i) are in line with those observed upon binding of PA200 to the s20S[(Toste Rêgo and da Fonseca 2019)] (Supplementary Table 1)."

	**i20S (6AVO) vs PA200-i20S-PA200**
**β1/β1i**	RMSD between 24 atom pairs is 0.984
**β2/β2i**	RMSD between 24 atom pairs is 0.573
**β5/β5i**	RMSD between 24 atom pairs is 0.722
	**s20S (7NAN) vs PA200-s20S-PA200 (6REY)**
**β1/β1i**	RMSD between 24 atom pairs is 0.821
**β2/β2i**	RMSD between 24 atom pairs is 0.362
**β5/β5i**	RMSD between 24 atom pairs is 0.426

Supplementary Table 1 shows the RMSD of the main catalytic residues between the catalytic cores (i20S and s20S) alone and bound to PA200

17. Figure 6A, D, E: Clarify the meaning of the different colored bars in the graphs. This is not currently evident from the figure legend.

R17: The legend has been modified to clarify this as follow: "**The red, blue and yellow colored bars correspond to PSME4/PA200, standard 20S catalytic subunits (PSMB5-7), and immuno 20S catalytic subunits (PSMB8-10), respectively.**" Moreover, the label for PA200/PSME4 has been homogenised to "PSME4" in all the panels in Figure 6.

18. Line 59: Replace "macromolecular machinery" with "macromolecular machine."

R18: The text has been modified accordingly.

19. Line 64: "Proteome" should read "proteasome."

R19: The text has been modified accordingly.

20. Line 163: Consider revising "further away" to "furthest away."

R20: The text has been modified accordingly.

21. Line 381: "Majority of tissues doesn't" should read "the majority of tissues do not."

R21: The text has been modified accordingly.

22. Line 66: Remove “actually”. It is unnecessary.

R22: The text has been modified accordingly.

23. Line 83: Consider replacing citations from MDPI journal with peer-reviewed primary literature published in reputable journals. Additionally, citing numerous studies while simultaneously referring to PA200 as “enigmatic” is contradictory.

R23: We thank the reviewer for this comment and have rephrased the sentence and also added the original citations to support the contradictory publications on PA200 activity. We would like to mention that the MDPI review we are referring to has been compiled by our lab. It is the only comprehensive review on PA200 so far and was rigorously peer-reviewed.

24. Line 194: “It was suggested” should be attributed or rephrased for clarity and specificity.

R24: We agree that this sentence lacked clarity and modified it as follows: **“The two teams who solved the s20S-PA200 structures and identified IP6 and 5,6[PP]2-IP4 in the positively charged pockets hypothesized that they could be involved in regulating the activity of PA200, and more precisely in the degradation of acetylated histones[20-21].”**

Reviewer #2 (Remarks to the Author):

The authors present a combined structural and biochemical study of PA200 binding to 20S. Overall, the study is very detailed and thorough, the figures and text clear, and a clear case is made as to the key advance presented. My expertise is in mass photometry, so I will focus my comments on that (small aspect) of the manuscript, although having read the entire paper, it seems a novel and clear piece of work overall that deserves publication. I have two comments that the authors may want to address prior to publication.

Major

25: I am not sure I really understand how the KDs were calculated and it is not clear to me that it was done correctly form a thermodynamic perspective. I have attached a document for the authors to consider regarding the calculation of the dissociation constants, presented in Figures 3 and S4:
It is not clear from the text how the authors performed the experiment, specifically what the absolute concentrations of the proteins were, rather than just the ratios. It is important to state it because, considering only the ratio is not enough for the calculation of the Kd.

The authors indicate that they used 0.28uM of S20. Was this the final concentration of the incubation or was it further diluted when PA200 was added? If this is the final incubated concentration and the PA200 was added at x8 concentration (as indicated in Figure 3), it is not possible that the Kd was around 20 nM and we can still see free S20.

R25: We thank the reviewer for this important comment. The 0.28 µM concentration that is mentioned in the article relates to the s20S stock concentration: the final concentration (after 6-fold dilution of the intermediate PA200-20S mix) was 20 nM. We apologize if this was not clear and modified the text (Material and Methods part) accordingly, as follows: **"Recombinantly expressed human PA200 was then added to the tubes to reach PA200:20S molar ratios ranging from 0 to 15, with a fixed 20S concentration of 120 nM. Tubes were incubated for 2 h on ice. The complexes were diluted six times immediately before measurement on the Mass photometer (Refeyn Ltd) to reach a final 20S proteasome concentration of 20 nM."**

26: A simple calculation gives: $[S20free]/[20Sonebound]=KD1/[PA200free]$
In figure 3 the authors indicate that the experiment was done at x8 concentration of PA200, and the $[S20free]/[20Sonebound]$ ratio seems to be 1 or 0.5 (for the cases of s20S or i20S, respectively). To get this ratio, the concentration $[PA200free]$ must be equal to $KD1$ or $2KD1$ which means between 20 and 40 nM. This implies that the concentration of s20S/i20S during incubation was only about 4–8 nM, very low for quantification with MP, specifically if a 6-fold dilution was done before the measurement.
A similar calculation can be performed for the second dissociation constant.

R26: We completely agree with the reviewer calculations. The determination of the Kd values were performed as shown in Figure 3, using a curve fit that takes into account the whole data set obtained for free PA200 concentrations up to 130 nM (plateau). Figure 3A corresponds to a PA200:20S molar ratio of 8, i.e. an initial PA200 concentration of 160 nM. At equilibrium, PA200 then distributes into free PA200, 20S-PA200 and 20S-(PA200)2. At this molar ratio of 8, the free PA200 concentration is around 140-150 nM and is above the free PA200 concentrations used to calculate the Kds (0-130 nM). For a reason that we cannot explain, the Kd obtained with the Hill curve fit are quite different from the ones we obtain using the calculations mentioned by Reviewer #2 (DOI: 10.1002/cpz1.962), which are around 80-100 nM for Kd1 and 200-225 nM for Kd2. The Hill curve fit takes into account the variability of the data set and since the goodness of fit seems acceptable (R^2 above 0.88 for the four Kds determined, see new Figure 3D), we therefore prefer to rely on these values.

27. Another issue concerns the choice of the x-axis in the calculation of the Hill plot. Usually the x-axis represents the measured/assumed concentration of the free ligand in equilibrium not the

initial concentration or the ratio of the initial concentrations as plotted here (as we understand). Again, it is unclear how the Kd can be inferred without the information of the absolute concentration at equilibrium on the x-axis.

R27: We have indeed used the concentration at equilibrium for the Kd calculations and not the molar ratios that were used just for representation of the data in Figure 3. We have now modified Figure 3 and replaced the molar ratio with the free PA200 concentration on the x axis (revised Figure 3B, C).

28.It is also not clear why the occupancy reaches a plateau at values of 0.4, one would expect saturation at much higher value.

[editorial note: unpublished, confidential material redacted]

29. The authors performed MP measurements that enable them to quantify the individual stoichiometries of the interactions. They have a direct measurement of the relative abundance of the free, one-bound and two-bound molecular states. Therefore, given the knowledge of the protein concentrations they should be able to directly calculate the dissociation constants and check for cooperativity. Hill plot is useful if one does not have the breakdown into different stoichiometries.

R29: We agree with the reviewer that, unlike SPR or ITC, mass photometry allows to reach microstates Kds by measuring the relative abundances of both singly and doubly bound 20S. We did perform Kd calculations this way (using values up to the saturation plateau, i.e. free PA200 concentration below 130 nM). This is now clearly shown in the revised version of Figure 3 (panel C). However, due to the variability of the measurement inherent to mass photometry, we obtained a substantial variability in the Kd1 and Kd2. Furthermore, the Kd2 values were surprisingly higher than Kd1 values (in contradiction with a potential positive cooperativity). We thus prefer to use a fitting of the data to obtain these Kds. In both cases, we did not observe Kd2 values lower than the Kd1, suggesting that there is no positive cooperativity.
So, despite the high Hill coefficients measured for the doubly bound 20S depicted in Figure 3D, we prefer not to suggest any positive cooperativity. The main difference we observed relates to the max occupancy (Bmax), as mentioned previously.
We are conscious that the way we interpreted the data suggested a higher affinity of PA200 for the i20S vs. s20S, but we think that the main difference resides in the Bmax levels of the singly and especially the doubly bound complexes. We have thus restrained from mentioning higher affinity or positive cooperativity and modified the text (discussion part) as follows: **"We solved cryoEM structures of the i20S singly or doubly-capped by PA200, and observed that binding of the first PA200 molecule induces major structural rearrangements in i20S — an effect not seen with the s20S. This likely explains the two-fold higher formation of the doubly capped i20S–PA200 complex at equilibrium, as well as the stronger activation of i20S by PA200 relative to s20S."**

30. In Figure S4, it is not clear what the y-axis represents, in terms of occupancy I would expect a nonmonotonic occupancy fraction for the one-bound (initially increase and then decrease)

[editorial note: unpublished, confidential material redacted]

Minor

31: Abstract: The way it is written in the past tense - it really is not clear what was done in this work, and what was done by the authors/others in the past. A simple ‘Here, …’ would help the reader to differentiate.

R31: The text of the abstract has been modified accordingly.

32. Line 99 KDs - enhanced formation of PA200 - enhanced how? Cooperative enhanced or enhanced for i20s compared to s20s? Not clear

R32: We cannot clearly conclude on cooperative enhancement of binding and activities, but what we observe for sure is an enhancement of PA200 occupancy and activation of i20S compared to s20S. That is why we revised the text as follows (lines 97-100): **“We solved cryoEM structures of the i20S, singly or doubly-capped by PA200, and observed that binding of the first PA200 molecule induces major structural rearrangements in the i20S — an effect not seen with s20S. This likely explains the two times higher ratio of the doubly capped i20S-PA200 complex at equilibrium, as well as the stronger activation of i20S by PA200 relative to s20S.”**

Reviewer's Comments:

Reviewer #1 (Remarks to the Author):

Zivkovic and colleagues addressed some of the concerns that I proposed in my previous reviewing, for example by clarifying and presenting new evidence for my point 1 (cryo-EM comparison with s20S-PA200).

Unfortunately, I am not fully convinced by the reply to my points 2 and 3-4. I am sorry to keep referring to in Javitt et al., Nat Cancer 2023 but the prominent authorship of S. Meiners in both studies request an extra effort in generating a comprehensive picture that provide a shaped knowledge to the audience. Something that could be in textbooks in the future. Re the two points:

Difference in the exp design can explain a different outcome and I can live with a discrepancy between exp. systems that is coupled to 'in system PA200 one does not affect much, in system 2 does affect i20S expression'. The revised discussion addresses this, although it leave me with an unclear understanding if PA200 indeed affect i20S expression.

We thank the reviewer for their comment and agree that the data were not fully clear. While we would love to be more precise here and provide text-book knowledge, we admit that the cellular function of PA200 is not fully understood. Our labs have spent almost 10 years working on PA200 and we are still not able to fully understand PA200 function in human cells. Two major caveats have to be considered:

First, regulation of i20S by PA200 depends on the expression level of the i20S subunits PSMB8-10 in cells co-expressing PA200. As shown in Figure S7 of our manuscript, expression of PA200 and PSMB8-10 does not primarily overlap. In immune cells, where the i20S is constitutively expressed, PA200 (PSME4) expression is very low on the protein level and is only mildly induced upon immune cell activation (Figure 1 for the Reviewer). These data suggest that the regulation of the i20S by PA200 might only take place in settings where both genes are co-expressed, as also discussed in our manuscript.

Second, the function of PA200 is highly cell- and context-dependent. We performed a comprehensive analysis of the effects of genetic PA200 deletion in two different lung cancer cell lines, H1299 and A549. In our manuscript, we only showed regulation of the immunoproteasome as we are currently publishing the other data set with a particular focus on the role of PA200 in lung cancer. In this study, we chose the lung cancer cell lines A549 and H1299 because of their different origin and mutational background (A549 primary lung adenocarcinoma with KRAS activating mutation, H1299 lung metastatic lymph node with loss of TP53). CrispRCas9-mediated deletion of PA200 in several clones revealed a striking divergence of the molecular effects in these cell lines with an entirely different transcriptomic response (Figure 2A-B for the Reviewer). These cell-specific effects might be explained by PA200-dependent regulation of histone degradation where changes in chromatin remodeling would confer cell- and context-specific effects.

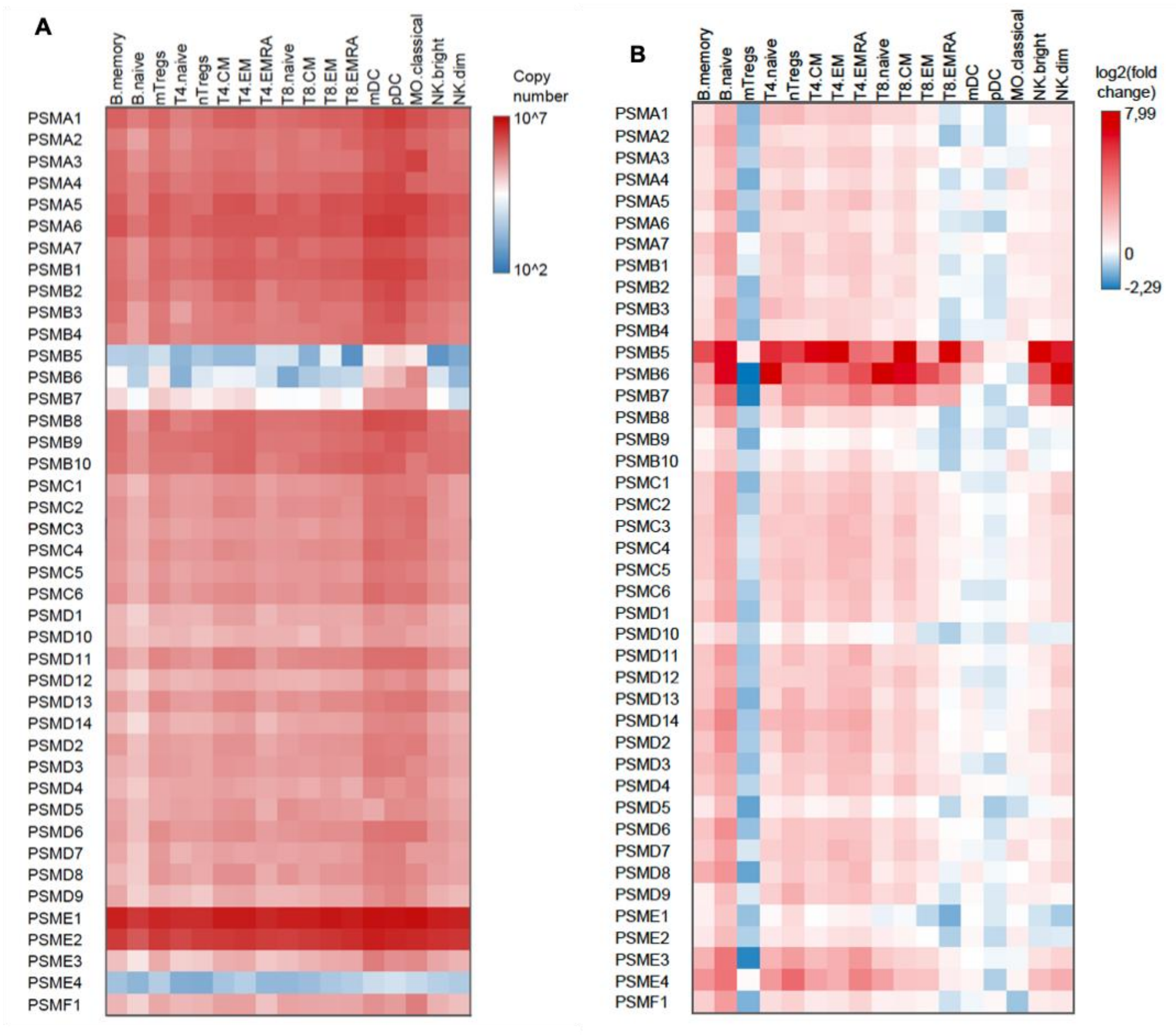

Figure 1 for the Reviewer: *Data extracted from the public database Immprot[1] where the authors used high-resolution mass spectrometry-based proteomics to characterize 28 primary human hematopoietic cell populations in steady and activated states at a depth of > 10,000 proteins in total. (**A**) Protein copy numbers of the different proteasome subunits (PSMA-F) at steady state. (**B**) Protein copy numbers (given as the relative log2 fold change in activated primary human hematopoietic cell populations versus steady state situation). Mean values are shown. PSMB5-7; standard proteasome, PSMB8-10: immunoproteasome. ([1] Rieckmann, J. C. et al. Social network architecture of human immune cells unveiled by quantitative proteomics. Nat. Immunol. 18, 583–593 (2017). http://www.immprot.org/).*

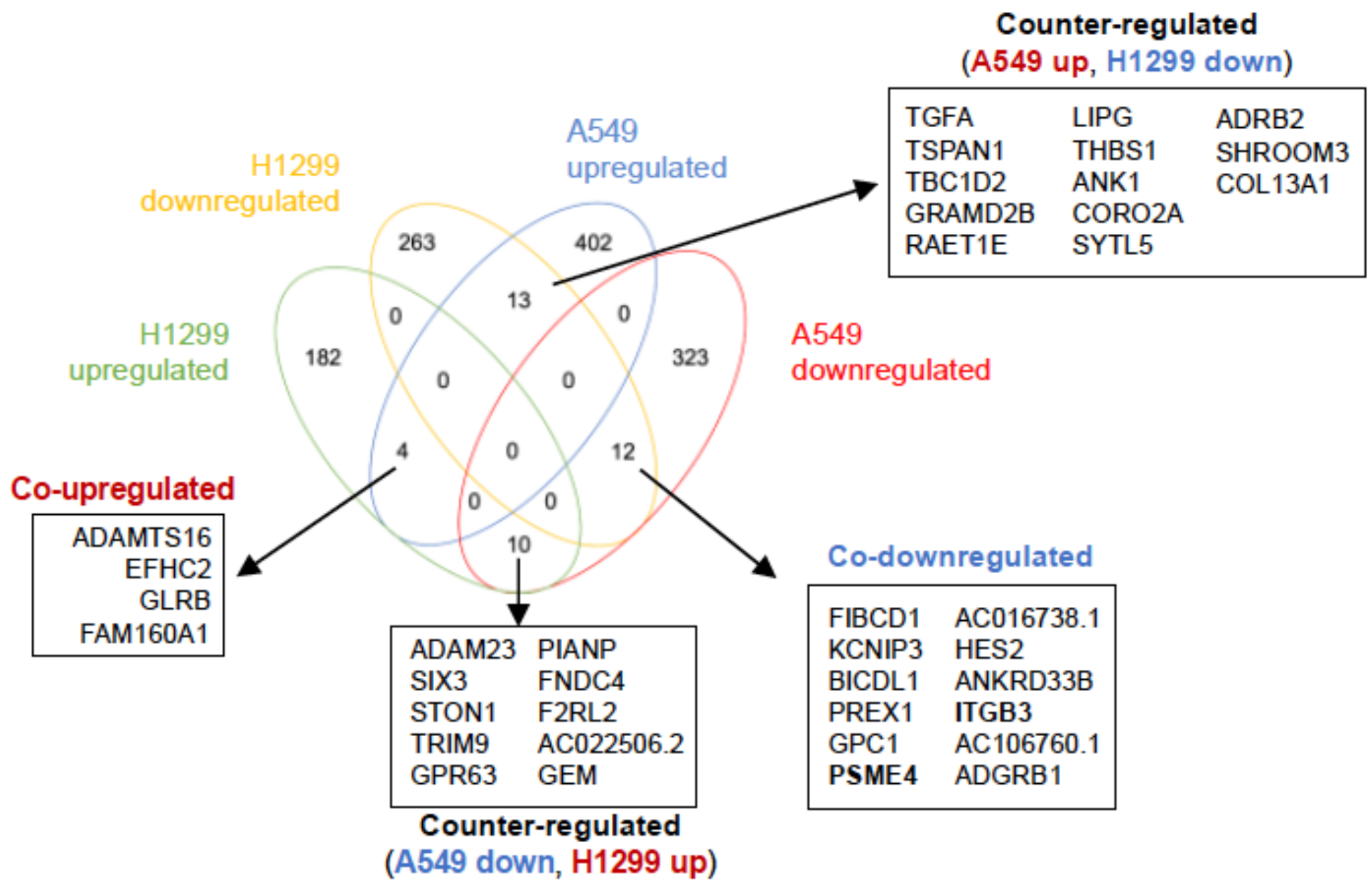

Figure 2 for the Reviewer: *The effects of PA200 deficiency in A549 and H1299 on transcriptional regulation were analyzed by RNA sequencing. We compared three WT and three KO clones of A549 and H1299 cells, respectively, for their transcriptional regulation. The results were filtered, and differentially expressed genes (DEGs) ($|FC| \geq 1$, $p \leq 0.05$, $FDR \leq 0.05$) between WT and KO cells were plotted in a Venn diagram.*

Taken together, while we observed downregulation of i20S subunits upon genetic deletion of PA200 in two cancer cell lines on the protein and proteasome interaction level, we cannot rule out that this is a cell-type and context-dependent effect which also depends on the co-expression of PA200 and the i20S in the cell. Accordingly, we rephrased our discussion in the revised manuscript which now reads: ***"Moreover, our recent analysis on PA200 in the two lung cancer cell lines A549 and H1299 revealed a striking disparity of cellular responses to PA200 deletion in these two cell lines with differential proteasome remodeling and transcriptional regulation[26]. These data suggest that PA200-mediated cellular effects are determined by the cellular state possibly due to PA200 functioning on the level of chromatin remodeling. The cellular interaction of PA200 with i20S will thus not only depend on the co-expression of PA200 and i20S but also on the cellular context."***

3-4. The authors have an explanation of why their results with using short fluorogenic peptides (SFPs) +/- PA200 generated contradicting results than Javitt et al., Nat Cancer 2023. I agree that the exp performed with purified s/i20S + PA200 could allow a clear investigation of what 20S+PA200 does. However, the contradiction with the results that were published in Javitt et al., Nat Cancer 2023 requires more investigation. Although the authors performed an assay with bovine histones, they reported that only in the reply to reviewers. That experiment, which was indeed what I suggested, did not show any significant difference between s20S and i20S. The justification that acetylated histones are the only commercially available for this assay does not stand. There have been several papers that successfully used proteins for the degradation by s20S and i20S in the last two decades. I am confident that many of those proteins could then also be well degraded by 20S+PA200. I think this experiment is pivotal otherwise I remain with a significant doubt and I am not convinced that the title of the manuscript is really supported by the data. The reality is that the SFP exp do not confirm what was published by Javitt et al., Nat Cancer 2023. If the authors could perform a new set of exp using proteins and measuring the peptide products, they could also significantly help in the understanding the impact of PA200 on s20S vs i20S degradation. With this, the manuscript would be improved. Without, I do not understand what the changes identified by cryo-EM lead to.

We thank the reviewer for this comment and have accordingly performed the experiments suggested by the reviewer. Specifically, we digested heat-denatured HEK cell protein lysates by addition of purified s20S or i20S proteasomes, with or without activation by recombinant PA200. Briefly, cell lysates were heated at 95 °C for 10 min to denature proteins, thereby promoting protein unfolding and facilitating degradation by PA200-activated proteasomes. Importantly, this heating step completely abolished endogenous proteasome activity (data not shown), thus preventing uncontrolled protein degradation by endogenous proteasomes.

Purified s20S or i20S (2 µg) and PA200 (6 µg) were then added to 100 µg of protein lysate at a controlled PA200:20S molar ratio of 8:1. The mixture was incubated for 3 h at 37 °C, and the

reaction was stopped by the addition of 5 µM bortezomib. The control condition consisted of protein lysates without the addition of either proteasomes or PA200 and addition of 5 µM bortezomib after 3 h at 37 °C.

Peptides were subsequently separated from intact proteins by ultrafiltration using 3 kDa cutoff filter devices, and 50 ng of each peptide mixture was analyzed by LC–MS/MS. Quantitative comparison using the control condition for unspecific peptide background subtraction allowed the identification of a total of 1,090 proteasome-derived peptides that were significantly enriched. A large overlap of at least 1,000 proteasome-derived peptides was identified in at least 3 replicates across the different replicates (n = 4 or 5, Supplementary Fig. S6 - revised manuscript and displayed below).

Analysis of the C-terminal amino acid distribution of these peptides revealed that the immunoproteasome displays the expected lower caspase-like activity and higher chymotrypsin- and trypsin-like activities compared to the s20S, regardless of PA200 activation. This observation is fully consistent with the well-established cleavage specificity of the immunoproteasome and thus validates our experimental approach (revised Supplementary Fig. S6).

Strikingly, the activation effect of PA200 was more pronounced for the i20S than for the s20S. Specifically, 66 versus 25 additional proteasomal peptides were generated by PA200-activated i20S compared with s20S (Figure 5A - revised manuscript and displayed below), and most of these peptides were specific to the activated i20S (Figure 5B).

Finally, a more detailed analysis of the C-terminal residues of peptides produced by the i20S revealed a marked shift in amino acid composition upon PA200 activation. Whereas unactivated i20S predominantly generated peptides with trypsin- and chymotrypsin-like C-termini (49% and 26%, respectively) and few caspase-like termini (6%), PA200 activation resulted in reduced tryptic-like peptides (27%) and increased caspase-like peptides (13%). Notably, 26% of peptides uniquely produced by PA200-activated i20S ended with an aspartate (D) or glutamate (E) (i.e. caspase-like peptides), while only 18% terminated with lysine (K) or arginine (R) (i.e. tryptic-like peptides) (Figure 5C - revised manuscript).

These data are in full agreement with the results reported by Merbl and colleagues, who showed that PA200 abundance is positively correlated with caspase-like specificity and negatively

correlated with trypsin-like cleavage. Our findings further reinforce their observation that PA200 negatively regulates the production of peptides capable of anchoring in the MHC-I binding groove. We have included these data into the revised version which now reads:

- *for the results section: paragraph entitled "**PA200 alters peptide generation by the immunoproteasome"** added between paragraphs "PA200 binds and activates the i20S more efficiently than the s20S" and "Enriched binding of i20S to PA200 in tissues": **To assess the impact of PA200 on proteasomal peptide generation, heat-denatured HEK cell lysates were digested with purified s20S or i20S proteasomes, in the presence or absence of recombinant PA200 at a defined PA200:20S molar ratio of 8:1. Heat-denatured lysates without added proteasomes served as the respective controls to exclude non-proteasomal peptides. Heating efficiently abolished endogenous proteasome activity (data not shown) and reactions were analyzed by LC–MS/MS after peptide isolation. Quantitative analysis using the control condition for unspecific peptide background subtraction allowed the identification of a total of 1,090 proteasome-derived enriched peptides. A large overlap of at least 1000 proteasome-derived peptides was identified in at least 3 replicates across the different replicates (n = 4 or 5, Figure S6A-B). C-terminal residue analysis of these proteasome-derived peptides confirmed the expected cleavage specificity of the immunoproteasome compared with s20S, characterized by reduced D/E and increased K/R and F/Y/W/L residues, corresponding to caspase-, trypsin-, and chymotrypsin-like activities, respectively, thereby validating our experimental approach (Figure S6B). Notably, PA200 activation had a stronger effect on i20S than on s20S, leading to the generation of a larger number of additional peptides (66 vs 25), most of which were specific to PA200-activated i20S (Figure 5A–B) and overlapping across replicates (54 of the 66 peptides and 21 of the 25 peptides were reproducibly detected in at least three replicates for the PA200-activated i20S and s20S, respectively, Figure S6C). Moreover, PA200 activation of i20S induced a marked shift in cleavage specificity indicative of decreased trypsin-like and increased caspase-like activities (Figure***

5C). In particular, 26% of peptides uniquely generated by PA200-activated i20S ended with D or E, whereas only 18% terminated with K or R (Figure 5A, right panel).

These results are consistent with previous reports[12,22] linking PA200 abundance to increased caspase-like and reduced trypsin-like proteasomal activity, and further support a role for PA200 in limiting the production of MHC-I–binding peptides.", *and associated figures (Figure 5 and Supplementary Fig. S6).*

- *for the discussion section "***We further examined the effect of PA200 on both the quantity and C-terminal composition of proteasome-generated peptides using in vitro digestion of heat-denatured proteins. These analyses not only confirmed a markedly stronger impact of PA200 on the i20S compared with the s20S, but also revealed a pronounced shift in cleavage specificity toward enhanced caspase-like activity. Notably, PA200-activated i20S generated a substantially higher number of additional peptides, many of which were uniquely associated with PA200 activation and exhibited a strong enrichment in acidic C-terminal residues. Together with previous reports linking PA200 abundance to increased caspase-like and reduced trypsin-like activity[22], these findings support a model in which PA200 limits the production of optimal MHC-I–binding peptides.***".

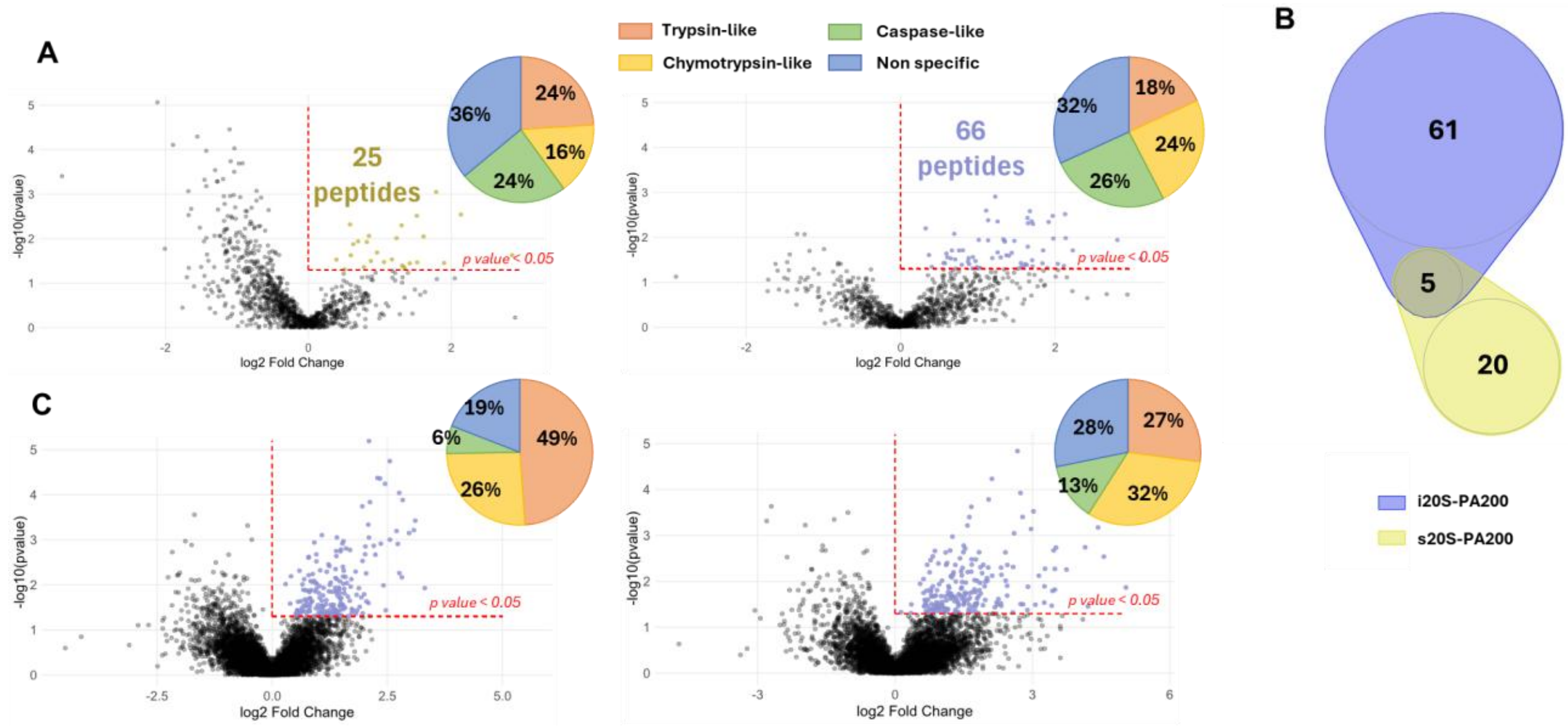

Figure 5 (revised manuscript): PA200 remodels peptides produced by the immunoproteasome. *Volcano plots highlighting the impact of PA200 on the C-terminal cleavage specificity and number of peptides generated by the immuno- (right panel) and standard- (left panel)-proteasomes (A). Venn diagram showing that proteasomal peptides generated by PA200-activated i20S are numerous and specific (B). C-terminal residue analysis showing how the i20S-derived peptides (highlighted in blue on the volcano plots) display a shift in the cleavage specificity without (left panel) or with (right panel) PA200 activation (C). The dashed red lines indicate the thresholds applied (FC and p value; a two-sided t-test was used, n = 4 or 5).*

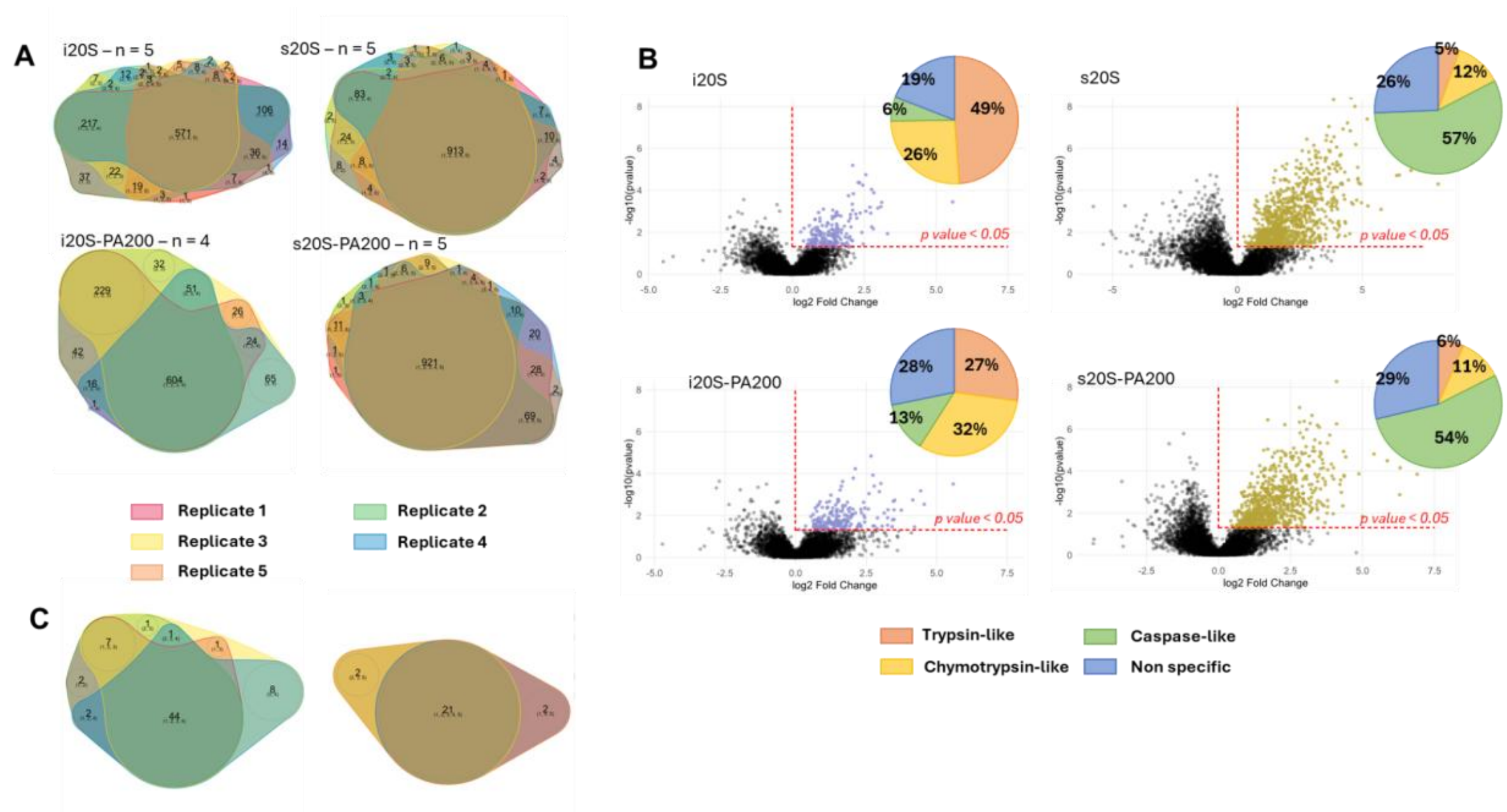

Supplementary Figure 6 (revised manuscript): *Venn diagram highlighting the overlap in the identification of proteasomal-peptides derived from free i20S and s20S, and PA200-activated i20S, and s20S (A). Volcano plots*

highlighting the peptides specifically generated by the different forms of proteasome (i20S, s20S, i20S-PA200, s20S-PA200) compared to control and the C-terminal residue analysis of these specific proteasomal peptides (B). The dashed red lines indicate the thresholds applied (FC and p value; a two-sided t-test was used, n = 4 or 5).

Reviewer #2 (Remarks to the Author)

The authors have carefully considered my previous comments on the mass photometry (MP) experiments and have revised the relevant sections accordingly. I will address only to their response to my previous comments. I agree with their conclusion that the results support preferential binding of PA200 to i20S over s20S, and I also recognise that precise affinity determination is not the main focus of their paper. Nevertheless, I think that the authors should consider the points below regarding their quantification methodology and decide whether reporting numerical values is significant for their publication.

The data demonstrate higher PA200 occupancy on i20S, as the authors state. However, the reported fitted dissociation constants indicate lower k_D values for s20S than for i20S, which is inconsistent with the observed and stated preferential binding. This discrepancy may result from the fitting approach applied. If the numerical values are to be reported and are significant, the authors should clarify or revisit the fitting strategy to ensure consistency or explicitly discuss this issue. Clarification of the MP equilibrium calculation. As I now understand the procedure, the samples were incubated at 120 nM 20S with 0 - 1.8 μM PA200 for 2 hours, then diluted six times immediately prior to the MP measurement. In my view, two distinct assumptions may lead to two valid approaches for calculating the interaction strength that will result in different values:

a. Rapid re-equilibration after dilution. If the authors assume that the system re-equilibrates rapidly following the dilution, then the equilibrium analysis should indeed use the post-dilution concentrations (20 nM), however, this assumption should be explicitly stated.

b. Negligible redistribution during measurement. If the measurement was performed immediately after dilution and the distribution is assumed (or shown) not to change during this short interval, then the incubation concentrations (120 nM 20S) should be used for the equilibrium calculation. The authors should clearly/justify which assumption applies.

Potential influence of PA200 oligomerisation. A plausible explanation for the early plateau in the binding curves is PA200 dimerisation. Depending on the dimerisation constant, this could considerably flatten the concentration increase of free monomeric PA200 and thereby influence the effective ligand concentration on the x-axis in Fig. 3C. Also, regardless of this cause, only the concentration of the free monomeric PA200 should be used for calculating binding constants, as this is the species relevant to the binding interaction.

Given these considerations, I believe the authors should decide the most appropriate representation of their data and, in particular, if numerical values are reported these should be referred to as apparent K_D.

We thank the reviewer for these insightful and constructive comments, with which we fully agree. We have carefully re-evaluated our mass photometry (MP) quantification strategy in light of these points and have revised the manuscript accordingly.

*First, regarding the effect of dilution on the equilibrium calculation, we now explicitly state our assumption that the system rapidly re-equilibrates after dilution. Based on the experimental timeline, we consider that equilibration occurs within the few minutes required to acquire the MP measurements. Consequently, all equilibrium calculations were performed using the post-dilution concentrations (20 nM 20S). This clarification has now been included in the revised manuscript, as requested by reviewer #2. In the results section: "**Using the relative proportions of free, singly- and doubly-capped 20S measured at equilibrium by mass photometry upon titration with increasing amount of PA200…**" and in the Methods section "**The free PA200 monomer concentration at equilibrium was calculated by using the initial PA200 concentration multiplied by the fraction of free PA200 monomer. Affinity calculations were performed using the method described in Kofinova et al. 2024[58] and, assuming a rapid re-equilibration after dilution, apparent KD values were obtained using final concentrations after dilution of the different species involved**."*

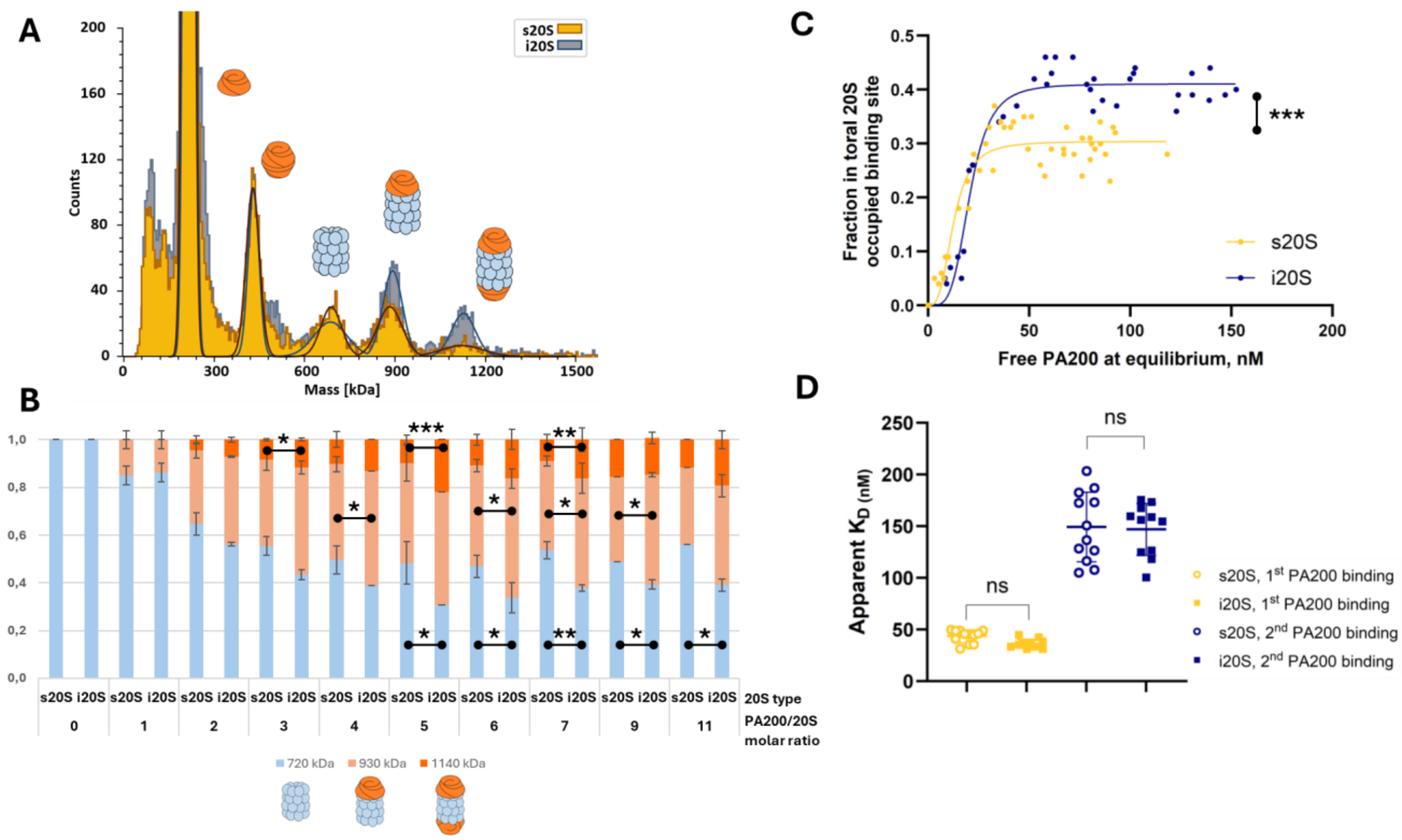

Figure 3 (revised manuscript): *i20S-PA200 particles show a higher occupancy compared to s20S-PA200 complexes. (A-B) Mass photometry allowed us to semi-quantify the relative abundances of free 20S (720 kDa), singly-capped 20S (930 kDa), and doubly-capped 20S (1,140 kDa). The PA200:20S molar ratio used in the graph shown in (A) was 8 but the range of molar ratios tested extends from 0 to 11 (B). (C) Titrations of PA200 to either s20S and i20S showed a higher total occupancy of the i20S vs. s20S. The total 20S occupied binding sites were calculated by summing the fractions of singly- and doubly-capped 20S, as detailed in the Materials and Methods section. (D) Apparent KD values obtained from mass photometry titration points (n ≥11) for the 1st and 2nd PA200 binding events show no differences in affinities for s20S and i20S.*

Second, we have taken into account only the concentration of free monomeric PA200 in our analysis. We agree with the reviewer that potential PA200 oligomerisation could contribute to the early plateau observed in the binding curves and affect the effective PA200 concentration. Since only monomeric PA200 is relevant for binding to 20S, we corrected our calculations accordingly. To further refine our analysis, we consulted with Dr. Jiří Kratochvíl (Kukura lab), an expert in MP-based affinity determination (doi: 10.1038/s41596-025-01255-4). Based on his recommendations, we revised our approach and calculated dissociation constants using individual titration points rather than relying solely on global fitting. We selected only titration points yielding consistent K_D *values and averaged them (n ≥ 11 for each KD).*

While this approach yielded K_D values that were overall comparable to those obtained from fitting—namely, similar affinities for s20S and i20S for both the first and second PA200 binding events—it resulted in higher K_D values for the second binding event (approximately ~150 nM using the titration-point method versus ~40 nM using fitting) in both cases. Importantly, given the remaining uncertainty in the exact concentration of free PA200, we fully agree with the reviewer that these values should be reported as apparent K_D values. The revised data are now presented in Figure 3D, and the term "apparent K_D" is consistently used throughout the manuscript.

Even though the K_D values can only be approximated, the revised analysis allows us to conclude that PA200 binds s20S and i20S with similar apparent affinities. At the same time, our MP data robustly demonstrate a significantly higher PA200 occupancy on i20S compared to s20S across most PA200/20S molar ratios tested (0–11; Figure 3B). This difference is reflected in distinct plateau values of 41 ± 2% for i20S and 30 ± 2% for s20S ($p < 0.001$; Figure 3C).

We further note that the plateaus do not reach 100%, which may result from partial dissociation induced by dilution prior to MP measurement.

[editorial note: unpublished, confidential material redacted]

With that in hand, we strongly believe that the occupancy differences observed between PA200-i20S and PA200-s20S are real and reflect the conformational

bending observed in our cryo-EM structure, leading to the observed differences in enzymatic activities.

[editorial note: unpublished, confidential material redacted]

Taken together, these results support the notion that PA200–20S interactions are intrinsically more dynamic. We further propose that the structural bending induced by the first PA200 binding event to i20S may favor the formation of a more stable and functionally activated complex, which is fully consistent with the higher proteolytic activities observed for i20S in our assays (Figure 4).

Reviewer #3 (Remarks to the Author)

The revision has improved the manuscript in several ways: the biochemical assays are more carefully documented, over-interpretation of the PA200/i20S expression link has been curtailed, and some of the more ambitious claims based on mass photometry have been softened. At the same time, the structural "bend" is only somewhat better supported (the new included movie is not convincing), and the manuscript remains weakest is the mass photometry–based thermodynamic analysis. The authors have clarified concentrations, re-plotted against free ligand, and added confidence intervals and goodness of fit. All of these are welcome. They have also backed away from strong claims about cooperativity and precise Kd values. Nonetheless, the fundamental issues are not resolved: simple equilibrium calculations and Hill fits yield incompatible Kd estimates for the same data; the unusually low maximal occupancies and the behaviour of singly-bound species are not convincingly explained. There is no orthogonal biophysical method to anchor the binding

parameters. The authors explicitly state that they do not understand why the analytical and fitted Kd values disagree. That is not a satisfactory thermodynamic reconciliation, and it means the mass photometry data should, at best, be treated as semi-quantitative support for a higher fraction of doubly capped i20S–PA200 under the chosen conditions, rather than as a rigourous determination of binding affinities or cooperativity. Taken together, the limitations in the depth and robustness of the structural and thermodynamic analysis remain. I stand my original assessment that the manuscript does not clear the bar for Nature Communication.

We agree with the reviewer that the bending and decompaction of the i20S-PA200 complex was not optimally visualized with our first morph animation from the s20S-PA200 structure (yellow) to the i20S-PA200 structure (blue, supplementary Video 1). We have now generated a new movie, based on the two same structures, but with slightly different orientation, slower timeframes and including two additional panels zooming on the proximal and distal PA200 molecules (see new supplementary Video 1). We think it now clearly shows both the bending and decompaction that the i20S complex undergoes upon binding of PA200 and that is not seen at all with the s20S proteasome. We hope the Reviewer will now find it satisfactory.

Concerning the mass photometry data analysis, we have carefully considered the remarks from Reviewers 2 and 3 and have substantially revised both our analysis and our interpretation of the results to address their concerns (see our response to Reviewer 2). Following advice from members of the Kukura lab (the developers of mass photometry), we are confident that our revised analysis now reveals significant results, while remaining cautious not to overinterpret them.

Reviewer #4 (Remarks to the Author)

I co-reviewed this manuscript with one of the reviewers who provided the listed reports. This is part of the Nature Communications initiative to facilitate training in peer review and to provide appropriate recognition for Early Career Researchers who co-review manuscripts.

REVIEWER COMMENTS

Reviewer #1 (Remarks to the Author):

Zivkovic and colleagues made an appreciated attempt to address my two concerns of the previous revised manuscript. I am sorry to say that I still have concerns. I hope that they can help to improve the manuscript.

Concern 1. I followed the explanation of the authors on the question: does PA200 indeed affect i20S expression? The statement that it depends on the cell state and the level of expression of both PA200 and immunoproteasome, bearing in mind that the conclusion is derived from 2 cell types is questionable. I think that the emphasis in the title and abstract and many sentences on this topic in the manuscript should be turned down. If in two cell background the authors see different things, either they increase the cell systems investigated or the reduce the emphasis of the claims.

R1: We thank the reviewer for his comment and have accordingly toned down our statement on the regulation of the i20S by PA200. Specifically, we have changed the title of the study to describe the more general concept of immunoproteasome regulation by PA200, softened and partly omitted the detailed discussion of the expression data in the discussion part.

Concern 2. The authors did not perform the experiment that I suggested but another one similar, which also has the potential of providing more information than the experiment that I proposed. Indeed, they performed a digestion of the entire cell lysate after heat denaturation using s20S, i20S +/- PA200. And performed DIA peptidomics on the samples after 1 h of processing by 20S-PA200. The idea is good. N=5 is a good exp. design. The presentation of the data significantly is less appreciated.

For example:

a. The authors do not show how many peptides are present in absence of proteasome (background). This does not let me appreciate the statistical power of the analysis and have an evaluation of the biological meaning of the output.

R2: This information was indeed missing and we thank the reviewer for helping us improve our manuscript. We realize that there was an inversion in the legend which might have affected the understanding of this figure by the reviewer. We apologize for this and have now corrected the legend of Figure S6. The volcano plots displayed in Figure S6A correspond to quantitative comparisons of peptides identified in the different conditions (i20S, s20S, i20S-PA200, s20S-PA200) compared to the control (no 20S added, only heated protein substrates were incubated). Each dot corresponds to a unique peptide sequence. In total, 17,615 peptide sequences were identified (all conditions gathered - validation at 1% FDR). Out of these, 1,090 peptides are statistically enriched in at least one condition compared to the control, which corresponds to 6.2 % of the total peptide sequences identified. This information was added in the revised manuscript, as requested by the reviewer: "Quantitative analysis upon unspecific peptide background subtraction under control conditions allowed the identification of a total of 1,090 proteasome-derived enriched peptides out of 17,615 peptides identified in total (all conditions and replicates) (Figure S6A)." *We also modified Figure S6 and its legend accordingly, to better explain our analysis and results.*

b. The authors do not do a comparison of many other basic features of the peptide products (peptide length distribution, protein origin, feature of the protein origin) and they rather focus only on the C-termini grouping.

R3: The biological impact of our results relates mainly to the activation of the i20S by PA200 and how this affects the peptide repertoire. Our analysis is in line with the study by Javitt et al. showing that PA200 can regulate proteasome activity in tumors, control antigen presentation and tumor immunity. We agree with the reviewer that peptide length is an important criterion, in addition to the nature of its C-terminal amino acid, for defining the optimal "antigenic format" of a peptide. We have therefore added an analysis of the length distribution of peptides generated by the different proteasomes and compared it to the length of cellular peptides identified as unspecific based on the control condition (see point c / R4 below for more details on this experiment). These results were included in Figure S6, panel C in the revised manuscript and are shown below. Our analysis confirms previously published results showing that proteasome-derived peptides have a median length of 8-10 amino acids, and this is observed whatever the subtype (s20S or i20S) and

whether the 20S is free or bound to PA200. We also confirmed that these proteasomal peptides exhibit a significantly shorter length than non-proteasomal peptides (detected as non-variant or under-represented in the volcano plots of Figure S6A). Concerning the protein origin, we are hesitant to present these data for two main reasons: 1- the proteins from the different cell compartments were mixed upon cell lysis, 2- the protein lysate was heated and proteins were unstructured in order to facilitate cleavage by PA200. For these reasons, the proteins degraded might not represent true endogenous substrates of PA200-activated 20S proteasomes. Therefore, we believe that any detailed analysis on the protein origins would be too risky at this stage.

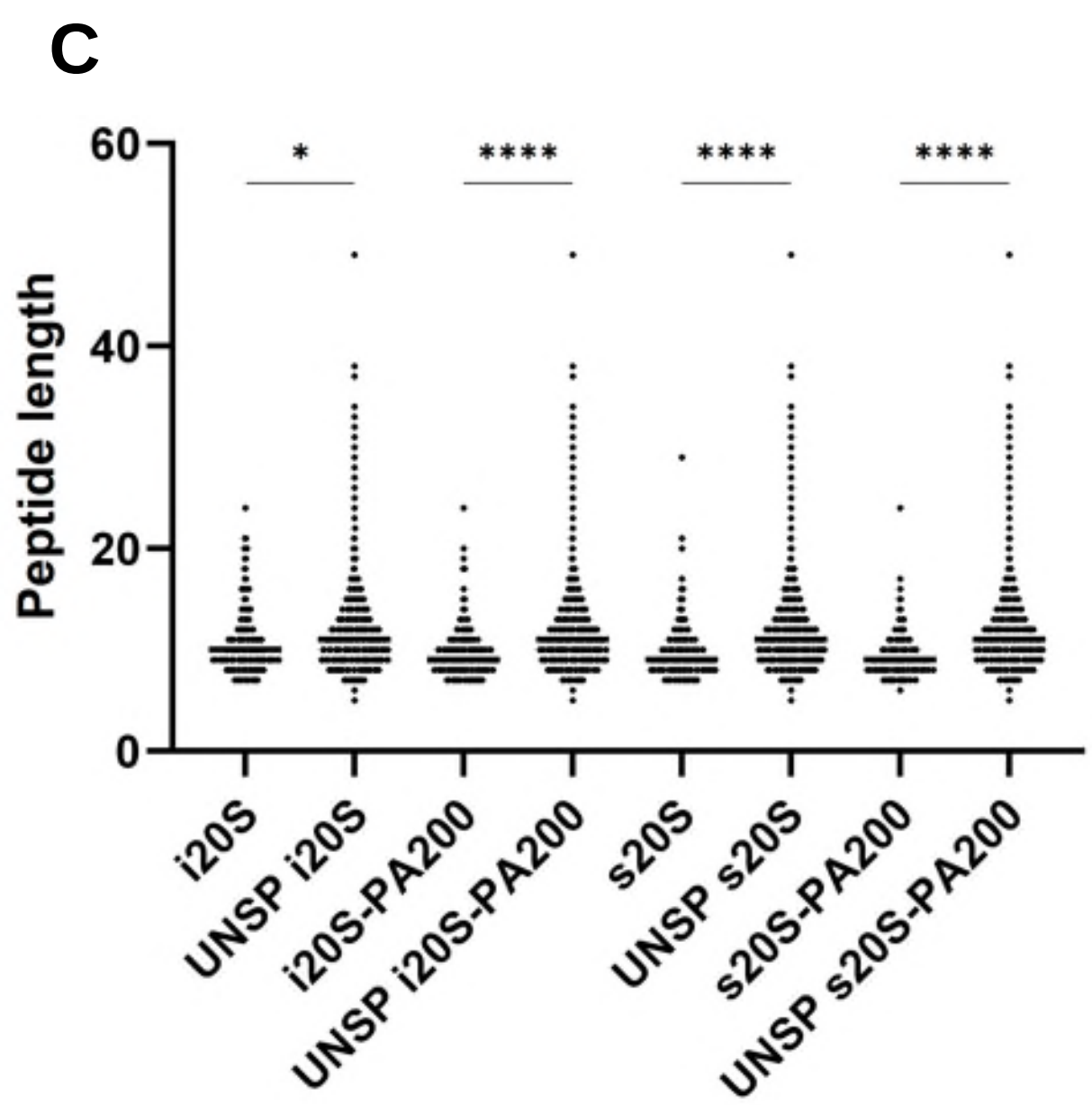

Revised Figure S6C: *Analysis of the length distributions of the peptides identified as proteasome-derived peptides of i20S, i20S-PA200, s20S, s20S-PA200, or as unspecific peptides (detected as under-represented or non-variant in the different pairwise comparisons with the control in panel A). A one-way ANOVA test was used for the pair-wise comparisons.*

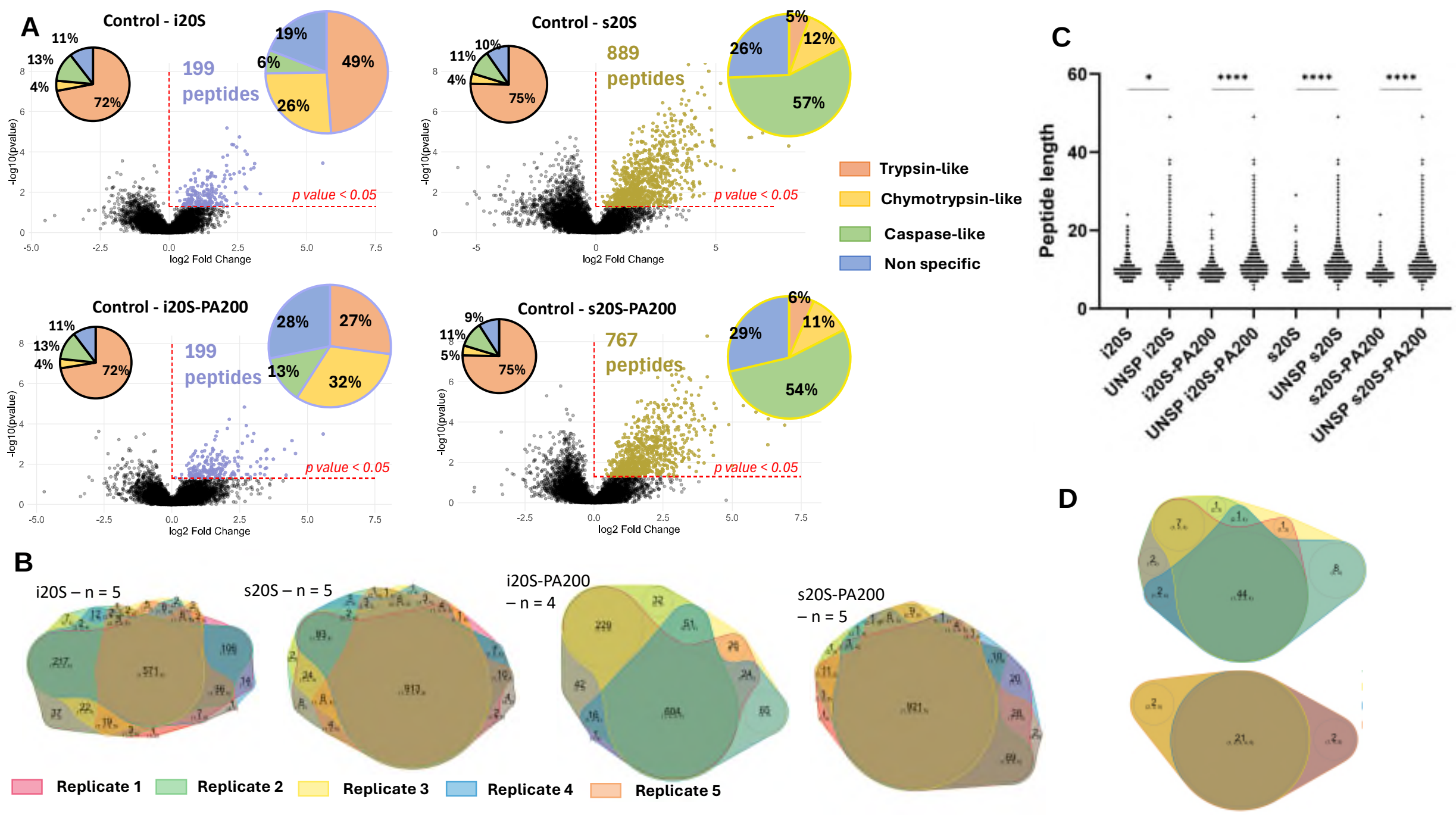

***Revised** Figure S6: **(A)** Volcano plots highlighting the peptides specifically generated by the different forms of proteasome (i20S and i20S-PA200 displayed as blue dots, s20S and s20S-PA200 displayed as yellow dots) compared to non-proteasomal peptides under control conditions (non-variant or under-represented peptides as black dots), and pie charts showing the C-terminal residue analyses of these specific proteasomal peptides (pie charts on the right of each volcano plot) compared to unspecific peptides (pie charts on the left of each volcano plot). The dashed red lines indicate the thresholds applied (fold change (FC = condition with proteasome / condition without proteasome, i.e., control) and p value; a two-sided t-test was used, n = 4 or 5). **(B)** Venn diagram highlighting the identification overlap across replicates of proteasomal peptides derived from free i20S (n = 5), s20S (n = 5), PA200-activated i20S (n = 4), and PA200-activated s20S (n = 5). **(C)** Analysis of the length distributions of the peptides identified as proteasome-derived peptides of i20S, i20S-PA200, s20S, s20S-PA200, or as unspecific peptides (detected as under-represented or non-variant in the different pairwise comparisons with the control in panel A). A one-way ANOVA test was used for the pair-wise comparisons. **(D)** Venn diagrams showing the identification overlap across replicates of the peptides specifically generated by the PA200-mediated activation of i20S (top) and s20S (bottom).*

c. Some controls are just reported but not shown (e.g., 'Proteasome substrate-based activity assays with the LLVY fluorogenic peptide were used to verify that the endogenous proteasome activity was completely abolished by the heating step.'. Not shown.

R4: We have now added the values obtained for the controls in the activity assay as Figure S7, as requested by the reviewer. We also have replaced "data not shown" by "99.6 ± 0.2 % of the activity lost after 10 min of heating – Figure S7)." in the revised manuscript.

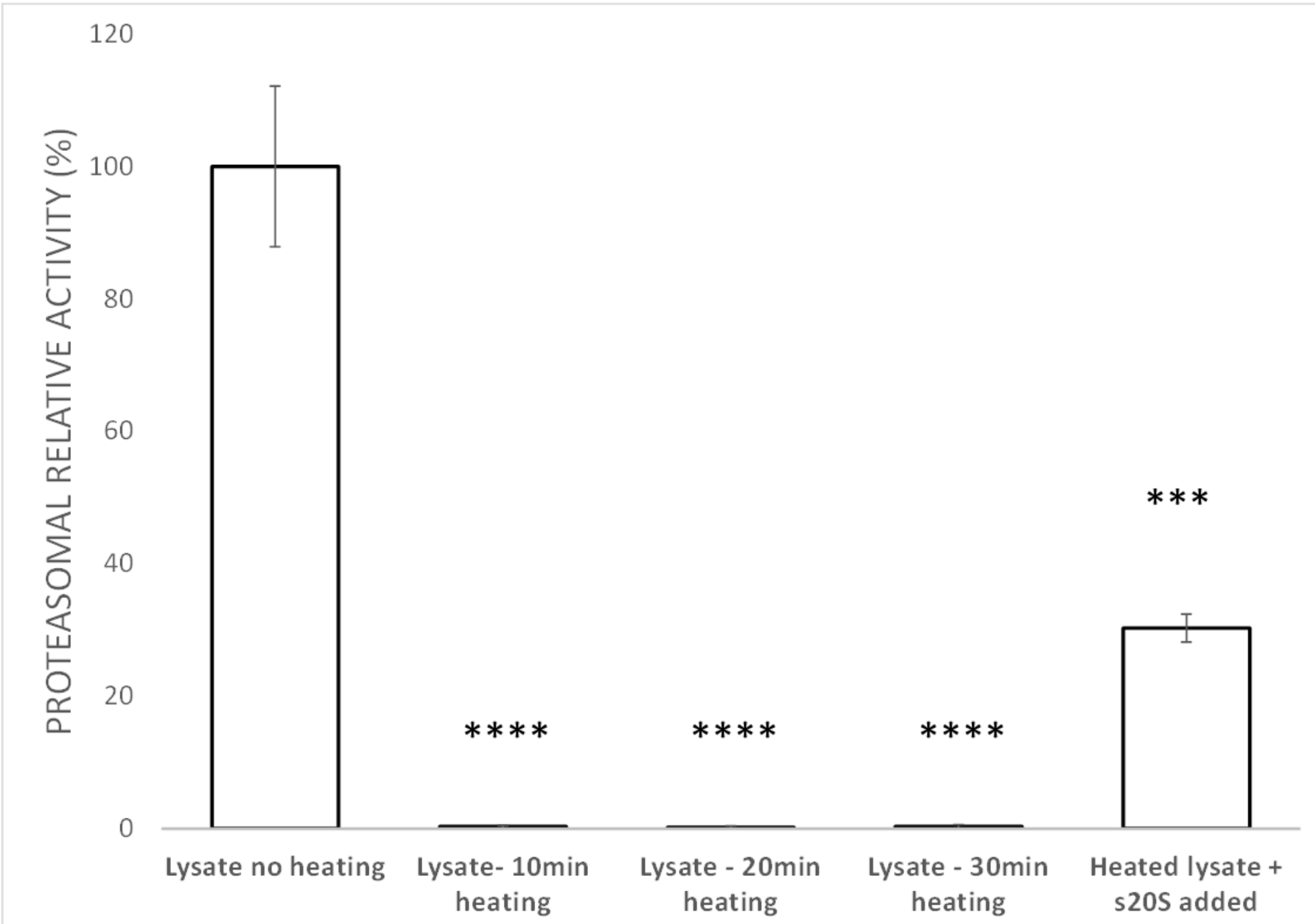

Revised Figure S7: *Chymotrypsin-like proteasome activity was measured using the LLVY fluorogenic peptide in HEK-EBNA cell lysates, either untreated or subjected to heat treatment at 95 °C for 10, 20, or 30 minutes. As a positive control, 5 µg of purified proteasome was added to the 5 minutes-heated lysate. The untreated lysate was used as a reference to calculate the percentage of residual activity under each condition. Statistical significance was assessed using a one-way ANOVA, with pairwise comparisons between each condition and the reference (***: $p < 0.001$; ****: $p < 0.0001$).*

d. I read: 'A large overlap of at least 1000 proteasome derived peptides was identified in at least 3 replicates across the different replicates (n = 4 or 5, Figure S6A-B)'. I do not understand what this means. Are 1000 peptides shared among all 4 conditions? How many peptides are identified per condition? The authors could decide a strategy to define a peptide as 'identified' (e.g., identified in 2 biol. Replicates) and then do a deeper analysis of their features (see above).

R5: The bioinformatic processing of the proteomic analysis involves a search into the database using all conditions and replicates. 17,615 peptides were identified in total, based on a FDR threshold below 1%, as indicated in the Materials and Methods section. However, to define if a peptide is a proteasome-derived peptide, we performed a pairwise quantitative comparison between each of the 4 "conditions" (degradation in presence of proteasome) and the control condition without the addition of purified proteasome, using at least 4 biological replicates (see volcano plots displayed in Figure S6A). Indeed, a peptide can be created de novo by a new proteasome cleavage, but can also be significantly increased thanks to proteasome activity, so this readout is more sensitive than the more basic comparison of peptides identified in each condition and control.

The 4 pairwise quantitative comparisons (condition vs control) of the 17,615 identified peptides required the quantification of each peptide in at least 3 replicates to calculate p-values and enabled to identify 199, 889, 199, and 767 over-represented peptides (i.e. proteasome-derived peptides) in the i20S, s20S, i20S-PA200, and s20S-PA200 conditions, respectively. The number of over-represented peptides in each condition has now been added in the revised version of Figure S6A). These over-represented peptides represent 18 to 81% of the total number of proteasomal-derived peptide sequences. These peptides (over-represented = proteasome-derived, and under-represented = unspecific peptides) were then used for the length (see R3 above and revised Figure S6C) and C-terminal residue analyses (see revised Figure S6A). Our strategy is now more clearly explained in the revised manuscript, as follows:

"Quantitative analysis using the control condition for unspecific peptide background subtraction allowed the identification of a total of 1,090 proteasome-derived peptides out of 17,615 peptides identified in total (all conditions and replicates) (Figure S6A). A large overlap of at least 1,000 proteasome-derived peptides was identified in at least 3 replicates across the different replicates (n = 4 or 5, Figure S6B) and among these, 18 to 81% were significantly enriched in each individual condition (i.e. i20S, s20S, i20S-PA200, s20S-PA200) when compared to the control (i.e., without proteasome) (Figure S6A)."

e. I understand that 21 peptides are identified in all biological replicates of s20S + PA200 compared

to s20S (fig. S6C). Is this part of the 921 peptides identified in all replicates of s20S+PA200? If so, it means that the authors are building an analysis of the difference in proteasome activity based on a 2% of peptides? How is this biologically relevant? Similarly, for i20S.

R6: The volcano plot on Figure 5A (left panel) shows that, among the 1,090-proteasome derived peptides identified in total (thanks to the results obtained from Figure S6A), only 25 peptides are statistically increased in the s20S-PA200 condition compared to the 20S alone condition, meaning that these peptides have been produced upon s20S activation by PA200. However, and quite convincingly, most (21) of these 25 significant peptides have been identified in all 5 biological replicates of the 's20S+PA200' condition (Fig. S6D, lower panel), showing that these data are robust. This indeed represents only 2.3% of the total proteasomal-derived peptides identified in total (i.e. in the 4 conditions with proteasome added- i20S, s20S, i20S-PA200, and s20S-PA200- when compared to the control condition -no 20S added). We agree with the reviewer that the number of peptides used for the C-terminal cleavage specificity analysis in this condition is low and have therefore erased this analysis in the revised version of Figure 5A (left panel).

However, the cleavage specificity analyses displayed in Figure S6A and Figure 5C were done using a higher number of peptides (i.e., 199, 889, 199, 767 peptides for i20S, s20S, i20S-PA200, and s20S-PA200, respectively), representing 18 to 81% of the total number of proteasome-derived peptides identified. We have added this sentence to the beginning of the section describing peptide length and C-terminal residues: "Despite the relatively low number of peptides included in this analysis, the peptide length distribution…".

These analyses clearly show:

1- *a reduced frequency of D/E but increased frequency of K/R and F/Y/W/L residues on the C-termini of generated peptides, corresponding to caspase-, trypsin-, and chymotrypsin-like activities when analyzing s20S and i20S proteasome-derived peptides, respectively.*
2- *that proteasome-derived peptides (over-represented peptides) generated in the 4 conditions (i20S, s20S, i20S-PA200, and s20S-PA200) exhibit very different C-terminal residue profiles when compared to unspecific i.e. under-represented peptides (controls – see new Figure S6A).*

This has been added in the revised manuscript "Moreover, proteasome-derived peptides exhibited a very different C-terminal residue profile compared to unspecific peptides (Figure S6A)."

3- *PA200 activation of i20S induced a marked shift in cleavage specificity compared to free i20S, indicative of decreased trypsin-like and increased caspase-like activities (Figure 5C and 5A, right panel):* "Moreover, PA200 activation of i20S induced a marked shift in cleavage specificity, indicative of decreased trypsin-like and increased caspase-like activities (Figure 5C)".

These findings strongly validate our experimental approach.

f. In Fig. 5A, C and Fig. S6B, I do not understand what is shown. Are they all peptides that have been identified? Is the analysis quantitative (as I would expect from the volcano plot and the DIA MS)? What is the relation of the peptides identified only in a condition and the quantitative analysis shown in the volcano plot? Are the T-like, CT-like and caspase-like activities in agreement between these two groups of peptides (those uniquely identified and those over-represented)?

R7: The volcano plots displayed in Figure S6A (and Figure 5C) display the pairwise comparisons of each of the four conditions (i.e. i20S, s20S, i20S-PA200, and s20S-PA200) compared to controls which did not contain any proteasome. This is indeed a quantitative analysis that focuses on the peptides produced by the respective 20S-containing complexes. All the 17,615 peptides are displayed (each peptide is represented by a dot). The under-represented peptides (black dots) are the unspecific peptides, while the over-represented peptides (colored dots) are the proteasome-derived peptides generated by the respective 20S complex.

Only the 1,090 proteasome-derived enriched peptides out of the 17,615 peptides identified in total (all conditions and replicates) have been used for the quantitative comparison displayed in Figure 5A, in order to focus our analysis on the effect of PA200 on the s20S vs. i20S activity.

The legends of these figures have now been revised to make this analysis approach clearer.

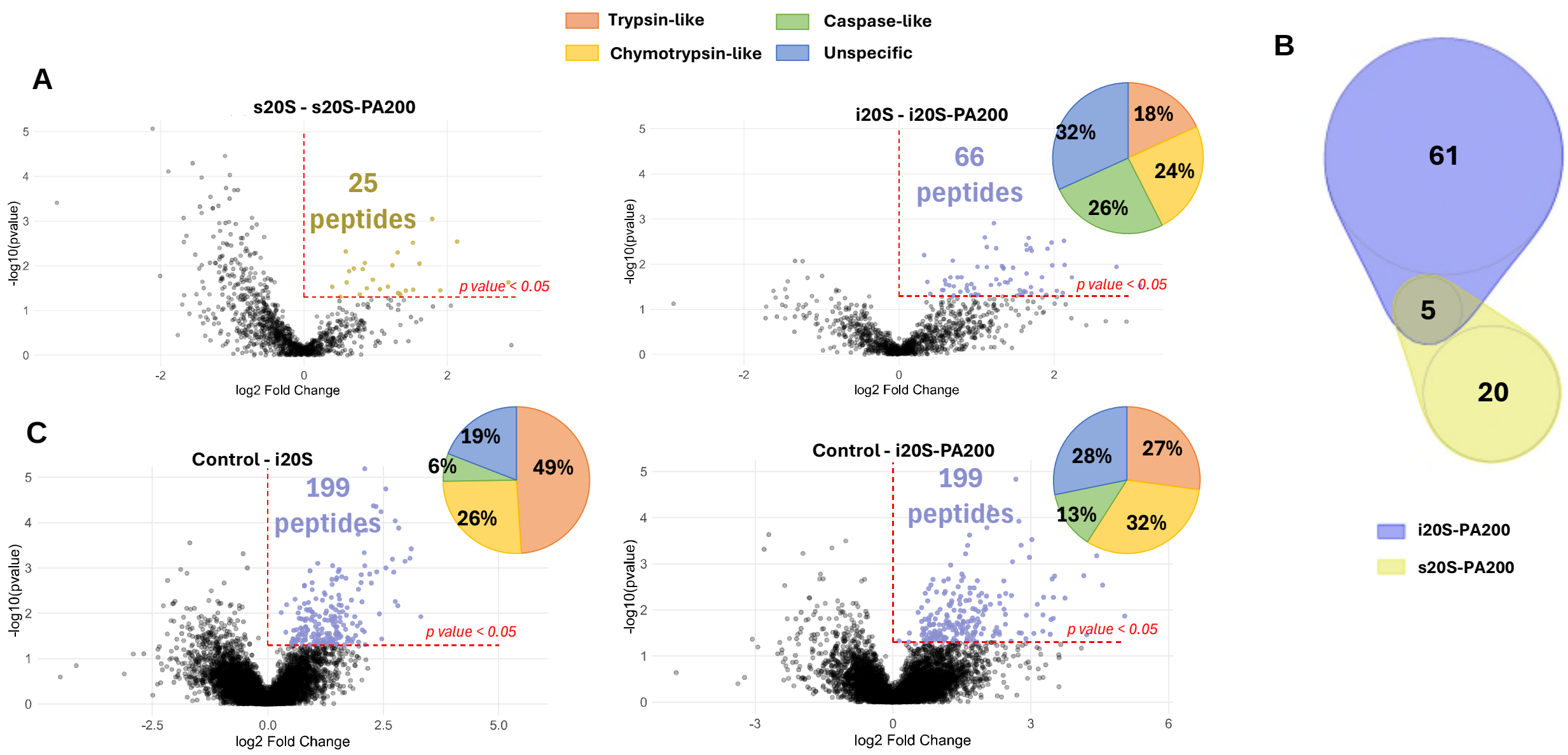

***Revised** Figure 5: **PA200 remodels peptide cleavage by the immunoproteasome. (A)** Volcano plots highlighting the impact of PA200 on the C-terminal cleavage specificity and number of proteasome-derived peptides generated by the s20S (left panel) and i20S (right panel). For this analysis, we used the 1,090 proteasome-derived peptides out of the 17,615 peptides identified in total (all conditions and replicates). **(B)** Venn diagram showing that 61 peptides are specific for PA200-activated i20S proteasomes (blue) compared to 20 peptides generated by PA200-activated s20S (yellow). **(C)** C-terminal residue analysis of proteasome-derived peptides (highlighted in blue in the volcano plots) generated by free i20S (left) and PA200-activated i20S (right), showing the shift in cleavage specificity upon PA200 activation. Black dots represent under-represented or non-variant peptides, i.e., unspecific peptides, and blue dots represent over-represented peptides, i.e. proteasome-derived peptides. The dashed red lines indicate the thresholds applied (FC and p value; a two-sided t-test was used, n = 4 or 5).*

g. In Fig. 5A,C and Fig. S6B, are the C-terminus groups different between those over-represented (top right; already analysed) and those under-represented (top left; not analysed)? Because I would expect an opposite effect on the right and the left of each volcano plot, according to the author hypothesis. This analysis could strengthen the conclusion of the authors that, as explained above, might be based on 2-6% of the peptides. This is quite a small portion of all peptides identified.

R8. This is indeed a very relevant proposition that strengthens our conclusions. In accordance with the reviewer's suggestion, we have now performed this analysis and added it as pie charts in the volcano plots displayed in the new Figure S6A (see above). Indeed, we observe opposite effects when comparing the C-termini of under-represented (i.e., unspecific peptides) and over-represented peptides (i.e. proteasome-derived peptides), strengthening our experimental

approach. A sentence has been added in the result part: “Moreover, proteasome-derived peptides exhibited a very different C-terminal residue profile compared to unspecific peptides (Figure S6A)”.

Minor:
I think Fig. 5C caption is missing
A new version of Figure 5 is now included with no caption missing.

Reviewer #2 (Remarks to the Author):

The authors have done an excellent job, I have no further comments.

Reviewer #3 (Remarks to the Author):

The authors have satisfactorily addressed my comments. I believe the manuscript is ready for publication.

Reviewer #4 (Remarks to the Author):

I co-reviewed this manuscript with one of the reviewers who provided the listed reports. This is part of the Nature Communications initiative to facilitate training in peer review and to provide appropriate recognition for Early Career Researchers who co-review manuscripts.

Regarding the calculation of the dissociation constants, presented in Figures 3 and S4:

1. It is not clear from the text how the authors performed the experiment, specifically what the absolute concentrations of the proteins were, rather than just the ratios. It is important to state it because, considering only the ratio is not enough for the calculation of the Kd.

   The authors indicate that they used 0.28uM of S20. Was this the final concentration of the incubation or was it further diluted when PA200 was added? If this is the final incubated concentration and the PA200 was added at x8 concentration (as indicated in Figure 3), it is not possible that the Kd was around 20 nM and we can still see free S20.

   A simple calculation gives:

   $$\frac{[S20_{free}]}{[20S_{onebound}]} = \frac{K_{D_1}}{[PA200_{free}]}$$

   In figure 3 the authors indicate that the experiment was done at x8 concentration of PA200, and the $\frac{[S20_{free}]}{[20S_{onebound}]}$ ratio seems to be 1 or 0.5 (for the cases of s20S or i20S, respectively). To get this ratio, the concentration $[PA200_{free}]$ must be equal to K_{D_1}or $2K_{D_1}$which means between 20 and 40 nM. This implies that the concentration of s20S/i20S during incubation was only about 4–8 nM, very low for quantification with MP, specifically if a 6-fold dilution was done before the measurement.

   A similar calculation can be performed for the second dissociation constant.

2. Another issue concerns the choice of the x-axis in the calculation of the Hill plot. Usually the x-axis represents the measured/assumed concentration of the free ligand in equilibrium not the initial concentration or the ratio of the initial concentrations as plotted here (as we understand). Again, it is unclear how the Kd can be inferred without the information of the absolute concentration at equilibrium on the x-axis. It is also not clear why the occupancy reaches a plateau at values of 0.4, one would expect saturation at much higher value.

3. The authors performed MP measurements that enable them to quantify the individual stoichiometries of the interactions. They have a direct measurement of the relative abundance of the free, one-bound and two-bound molecular states. Therefore, given the knowledge of the protein concentrations they should be able to directly calculate the dissociation constants and check for cooperativity. Hill plot is useful if one does not have the breakdown into different stoichiometries.
4. In Figure S4, it is not clear what the y-axis represents, in terms of occupancy I would expect a nonmonotonic occupancy fraction for the one-bound (initially increase and then decrease)
